# Predominant synthesis of giant myofibrillar proteins in striated muscles of the long-tailed ground squirrel *Urocitellus undulatus* during interbout arousal

**DOI:** 10.1038/s41598-020-72127-y

**Published:** 2020-09-16

**Authors:** Svetlana Popova, Anna Ulanova, Yulia Gritsyna, Nikolay Salmov, Vadim Rogachevsky, Gulnara Mikhailova, Alexander Bobylev, Liya Bobyleva, Yana Yutskevich, Oleg Morenkov, Nadezda Zakharova, Ivan Vikhlyantsev

**Affiliations:** 1 Laboratory of the Structure and Functions of Muscle Proteins, Institute of Theoretical and Experimental Biophysics, Russian Academy of Sciences, Pushchino, Moscow Region 142290 Russia; 2 Laboratory of Signal Perception Mechanisms, Institute of Cell Biophysics, FRC PSCBR, Russian Academy of Sciences, Pushchino, Moscow Region 142290 Russia; 3 Kuban State University, Krasnodar, Krasnodar Krai 350040 Russia; 4 Laboratory of Cell Culture and Tissue Engineering, Institute of Cell Biophysics, FRC PSCBR, Russian Academy of Sciences, Pushchino, Moscow Region 142290 Russia; 5 Laboratory of Natural and Artificial Hypobiosis Mechanisms, Institute of Cell Biophysics, FRC PSCBR, Russian Academy of Sciences, Pushchino, Moscow Region 142290 Russia

**Keywords:** Electron microscopy, Cytoskeletal proteins, Histology, Biochemical assays, Proteases, Proteolysis

## Abstract

Molecular mechanisms underlying muscle-mass retention during hibernation have been extensively discussed in recent years. This work tested the assumption that protein synthesis hyperactivation during interbout arousal of the long-tailed ground squirrel *Urocitellus undulatus* should be accompanied by increased calpain-1 activity in striated muscles. Calpain-1 is known to be autolysed and activated in parallel. Western blotting detected increased amounts of autolysed calpain-1 fragments in the heart (1.54-fold, *p* < 0.05) and m. longissimus dorsi (1.8-fold, *p* < 0.01) of ground squirrels during interbout arousal. The total protein synthesis rate determined by SUnSET declined 3.67-fold in the heart (*p* < 0.01) and 2.96-fold in m. longissimus dorsi (*p* < 0.01) during interbout arousal. The synthesis rates of calpain-1 substrates nebulin and titin in muscles did not differ during interbout arousal from those in active summer animals. A recovery of the volume of m. longissimus dorsi muscle fibres, a trend towards a heart-muscle mass increase and a restoration of the normal titin content (reduced in the muscles during hibernation) were observed. The results indicate that hyperactivation of calpain-1 in striated muscles of long-tailed ground squirrels during interbout arousal is accompanied by predominant synthesis of giant sarcomeric cytoskeleton proteins. These changes may contribute to muscle mass retention during hibernation.

## Introduction

The state of torpor—a reduced physiological activity peculiar of various animal kingdom representatives, birds and mammals including—is widespread in nature^1^. Mammalian torpor, hibernation, is an ability of some warm-blooded animals to adapt to unfavourable environmental conditions (low temperature and starvation) by reducing the activity of all physiological systems^1,2^. Long-tailed ground squirrels *Urocitellus undulatus* are obligate hibernators^3,4^. In these animals, hibernation lasts for 5–8 months and consists of bouts up to 30 days long followed by short arousals for periods of several (up to 24) hours. During the bout, the body temperature of the animal goes down to 2–4 °C; heartbeat rate diminishes from 110–420 to 3–5 bpm; oxidative metabolism decreases more than 100-fold; the frequency of respiratory movements sharply reduces and blood pressure decreases^3,4^.

During hibernation, the skeletal muscles of the animals are known to undergo atrophic changes^5^. Herewith, significant intra- and inter-species differences in the atrophy of various skeletal muscles have been revealed^5–7^. Most research deals with hind-limb muscles, e.g., such as m. soleus and m. gastrocnemius. Much less attention has been paid to body muscles, in particular, longissimus dorsi—one of the most powerful and long muscles of the spine, which subdivides into three parts: m. longissimus capitis, m. longissimus thoracis and m. longissimus lumborum^8,9^. Interest of investigators in this muscle has risen recently^10,11^. The function of the muscle is to unflex the spinal column, bend it to one side and maintain the body in a vertical position, when the animal is in an active state. During torpor, this muscle, unlike the muscles of the limbs, may contribute to maintaining the curled posture, which is so typical of deep hibernators. The grounds for this assertion can be the data on a considerable fast-to-slow muscle fibre shift occurring in m. longissimus dorsi during hibernation^12^. For this reason, research into this muscle of the long-tailed ground squirrel may contribute to a better understanding of the molecular mechanisms of muscle-system adaptation in hibernators.

Muscle mass and protein content are reduced during hibernation^5^, but the reductions are less significant than those observed in muscles of non-hibernating animals in traditional disuse models^13,14^. In particular, in non-hibernating mammals, prolonged disuse of skeletal muscles under conditions of real or simulated microgravity leads to a substantial loss of myofibrillar proteins such as titin and nebulin^13,14^, a damage of the highly ordered sarcomeric structure, as well as a decrease of the contractile ability of these muscles^15–17^. In atrophied striated muscles of hibernating animals similar changes have not been recorded^5,18–22^. The diaphragm and heart of hibernators are more resistant to atrophy than the skeletal muscles^5,23^. In the golden hamster (*Mesocricetus auratus*)^24^ and golden-mantled ground squirrel (*Callospermophilus lateralis*), however, the diaphragm and heart are observed to be hypertrophied during hibernation^25–27^.

Molecular mechanisms lying at the core of muscle mass retention in hibernators have been broadly discussed recently^23,28–38^. Various mechanisms are suggested to be responsible both for the decrease of atrophy development during torpor and for the activation of protein synthesis and muscle mass retention during arousals. It has been shown that inhibition of skeletal muscle atrophy during torpor in thirteen-lined ground squirrels (*Ictidomys tridecemlineatus*) may occur through downregulation of MyoG and inactivation of Foxo4^32^. These proteins regulate the transcription of various genes including the E3 ubiquitin ligases MAFbx and MuRF1, which are known to be activated in skeletal muscles under atrophy-inducing conditions^39^. Proteomic changes in two skeletal muscles of Daurian ground squirrels (*Spermophilus dauricus*) in pre-hibernation, hibernation and post-hibernation states have been recently analysed by iTRAQ-based quantitative analysis^33,38^. The total proteolysis rates of soleus in the hibernation and post-hibernation groups^33^ and the total proteolysis rate of extensor digitorum longus in the hibernation group^38^ are significantly suppressed as compared with those in the pre-hibernation group. It has been suggested that partial suppression of myofibrillar proteolysis and myofibrillar remodelling may be the basis of preventing the development of skeletal muscle atrophy in Daurian ground squirrels during hibernation^33^.

The role of protein synthesis activation during arousals, which may contribute to the decrease of the total muscle atrophy in hibernators during the hibernation period, has also been discussed^28,29,40^. The mTOR signalling pathway is critical to this process^28,29^. In particular, it has been tested over six stages of the torpor–arousal cycle of hibernation in thirteen-lined ground squirrels and found to be suppressed in skeletal but not cardiac muscle during torpor^29^. Periods of enhanced mTOR signalling are observed both in the skeletal and cardiac muscle: in the skeletal muscle, during the entrance into torpor and the arousal from torpor; in the cardiac muscle, during the arousal. Those results indicate that new protein synthesis is required in these two periods of hibernation^29^. The authors^29^ believe that the observed changes may lie at the core of physiological consequences for these striated muscles over the hibernation cycles: the tendency to atrophy in skeletal muscle and to hypertrophy in cardiac muscle.

The role of the Ca^2+^-calcineurin-NFAT signalling pathway in the decrease of the development of skeletal muscle atrophy and cardiac muscle hypertrophy in thirteen-lined ground squirrels during hibernation has been discussed^30,31^. An important role in the activation of this signalling cascade is known to be played by calpain-dependent activation of calcineurin^41^.

Calpains are nonlysosomal calcium-activated cysteine proteases^42,43^. There are 16 known components of the calpain system in mammals including 15 calpain proteases and the inhibitor calpastatin^44,45^. Striated muscles in mammals contain substantial amounts of two calpains: µ-calpain (calpain-1) activated at micromolar concentrations of Ca^2+^ and m-calpain (calpain-2) whose activation occurs at millimolar concentrations of Ca^2+^^44^. In the presence of Ca^2+^, calpain-1 (MW, 80 kDa) is autolysed, which leads to the formation of two fragments of 78 and 76 kDa^46,47^. In vitro data show that the autolysis and activation of calpain-1 occur in parallel^46^.

It is known that some proteins of thick and thin filaments in sarcomeres of striated muscles are proteolysed by calpains, in particular, calpain-1^44,48,49^. Giant muscle proteins titin and nebulin are also substrates of calpain proteases. It is hypothesised that myofibrillar protein turnover is triggered by calpain-dependent proteolysis of titin, nebulin and other proteins, whose fragments are then degraded to amino acids via the ubiquitin–proteasome pathway^45,49^.

The contents of such ligases as MAFbx and MuRF1, which are part of the ubiquitin proteasome system, increase in the cardiac muscle of thirteen-lined ground squirrels during the early arousal^50^. The content of MAFbx in the heart remains elevated during interbout arousals^50^. Considering these data^50^, as well as those on the activation of the mTOR complex in striated muscles of thirteen-lined ground squirrels during the early arousal^29^, we assumed that increased autolysis of calpain-1, which leads to enhanced activity of this enzyme, should take place in striated muscles during the interbout arousal. We also assumed increased syntheses of titin, nebulin and other proteins during the interbout arousal.

In this research, we studied seasonal changes in the autolysis of calpain-1 as well as in the contents of calpain-1, calpastatin, titin and nebulin in skeletal muscle m. longissimus dorsi (the activity of which is inhibited during hibernation) and in cardiac muscle (which continues to function) of long-tailed ground squirrels *Urocitellus undulatus*. Changes in total protein synthesis and also in the syntheses of nebulin and titin in muscles during interbout arousals were examined. Additionally, seasonal changes in the content of heat shock protein 90 (Hsp 90) were studied, as it had been shown to play a role in the regulation of calpain-1 activity^51^.

## Results

### Analysis of atrophic changes in striated muscles of long-tailed ground squirrels

**Figure 1:**
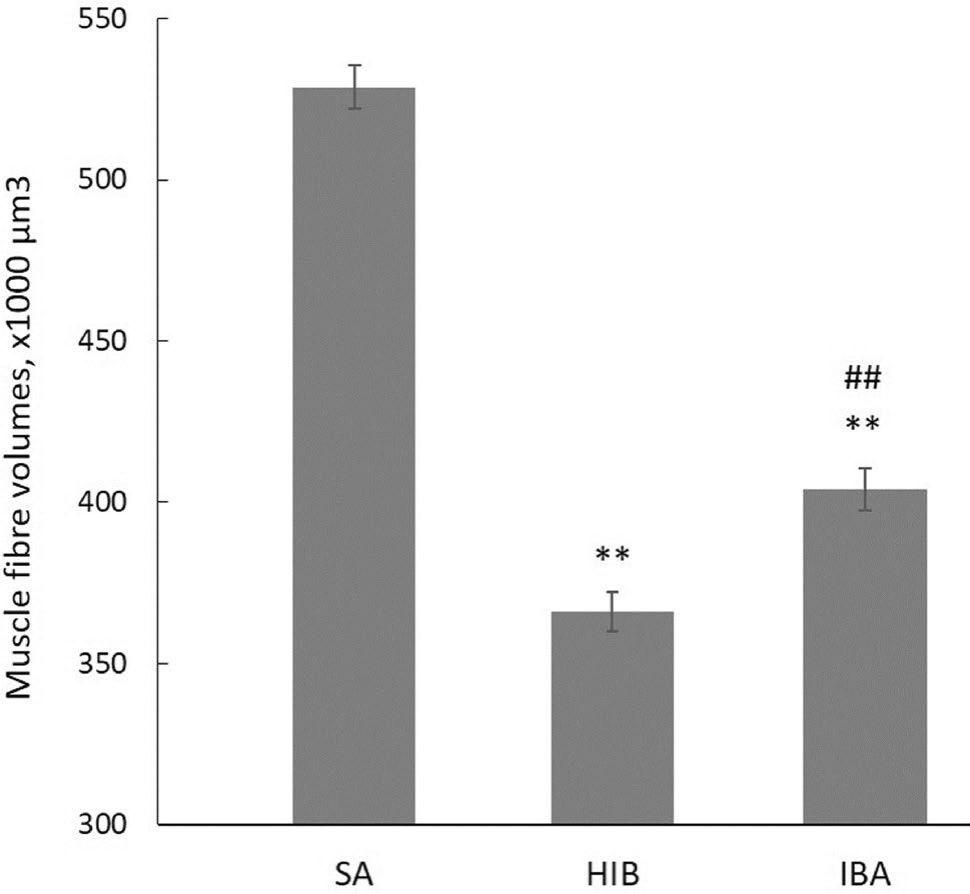
Muscle fibre volumes of m. longissimus dorsi of ground squirrels. *SA* summer activity, *HIB* hibernation, *IBA* interbout arousal. Animals from the SA (*n* = 5), HIB (*n* = 5) and IBA (*n* = 4) groups were used. The volumes of 300–375 fragments of muscle fibres were calculated for each animal group (Supplementary Tables S8, S9). Image alignment was made using the IGL Align sEM Align program. The 3D images of muscle fibres were formed using IGL Trace software. Contours of muscle fibres were manually retouched in each image (Supplementary Figs. S1, S2). Quantitative parameters were calculated using 3D View 3.5 software. The statistical analysis of the results was carried out with SigmaPlot 11.0, from Systat Software, Inc., San Jose California USA, https://www.systatsoftware.com. The data were analysed using nonparametric single-factor dispersion analysis for repeated measurements (Kruskal–Wallis One Way Analysis of Variance on Ranks) followed with the pairwise comparison by the Tukey’s test. Values are means ± SEM (SD = 129.3 for SA, 119.9 for HIB and 113.3 for IBA). **p < 0.01 (vs SA), ^##^*p* < 0.01 (IBA vs HIB).

A decrease in the weight of animals from the hibernation (HIB) group (by 20.7%, *p* < 0.01) and a tendency towards a weight loss of animals from the interbout arousal (IBA) group (by 11.7%) were observed as compared to the summer active (SA) group (Table 1; Supplementary Tables S1, S4). As compared with animals weighed before the hibernation season began (early November), a decrease in the weight of animals from the HIB group was 22.4% (*p* < 0.01); from the IBA group, 15.9% (*p* < 0.01) (Supplementary Table S5). No reliable differences in the heart weight and heart weight/body weight ratio in ground squirrels from the three experimental groups (SA, HIB, IBA) were found; however, there was a tendency towards an increase in the heart weight/body weight ratio in ground squirrels from the HIB and IBA groups (Table 1; Supplementary Tables S2, S3). Three-dimensional reconstruction showed a decrease (by 30.8%, *p *˂ 0.01) of muscle fibre volume in m. longissimus dorsi in the HIB group as compared to the SA group (Fig. 1, Supplementary Table S8; Supplementary Figs. S1, S2), indicating the development of atrophic changes in this muscle during hibernation. The volume of muscle fibres in m. longissimus dorsi in the IBA group was lower by 23.6% (*p* < 0.01) relative to the SA group, but higher by 7.2% (*p* < 0.01) than in the HIB group (Fig. 1, Supplementary Table S8; Supplementary Figs. S1, S2) which testifies to the dynamics of fibre volume recovery in this muscle in IBA animals.

**Table 1:** Animal body weight, heart weight and heart weight to animal body weight ratio.

Groups	Animal body weight, g	Heart weight, g	Heart weight/animal body weight, mg/g
SA, *n* = 7	666.7 ± 69.6	3.60 ± 0.85	5.45 ± 1.36
HIB, *n* = 7	528.9 ± 70.8**	2.96 ± 0.49	5.60 ± 0.68
IBA, *n* = 7	588.7 ± 45.8	3.42 ± 0.45	5.80 ± 0.89

Values are means ± SD.

*SA* summer activity, *HIB* hibernation, *IBA* interbout arousal.

**Significant difference vs SA, *p* < 0.01.

### Western blot analysis of calpain-1, calpastatin and Hsp 90 contents

**Figure 2:**
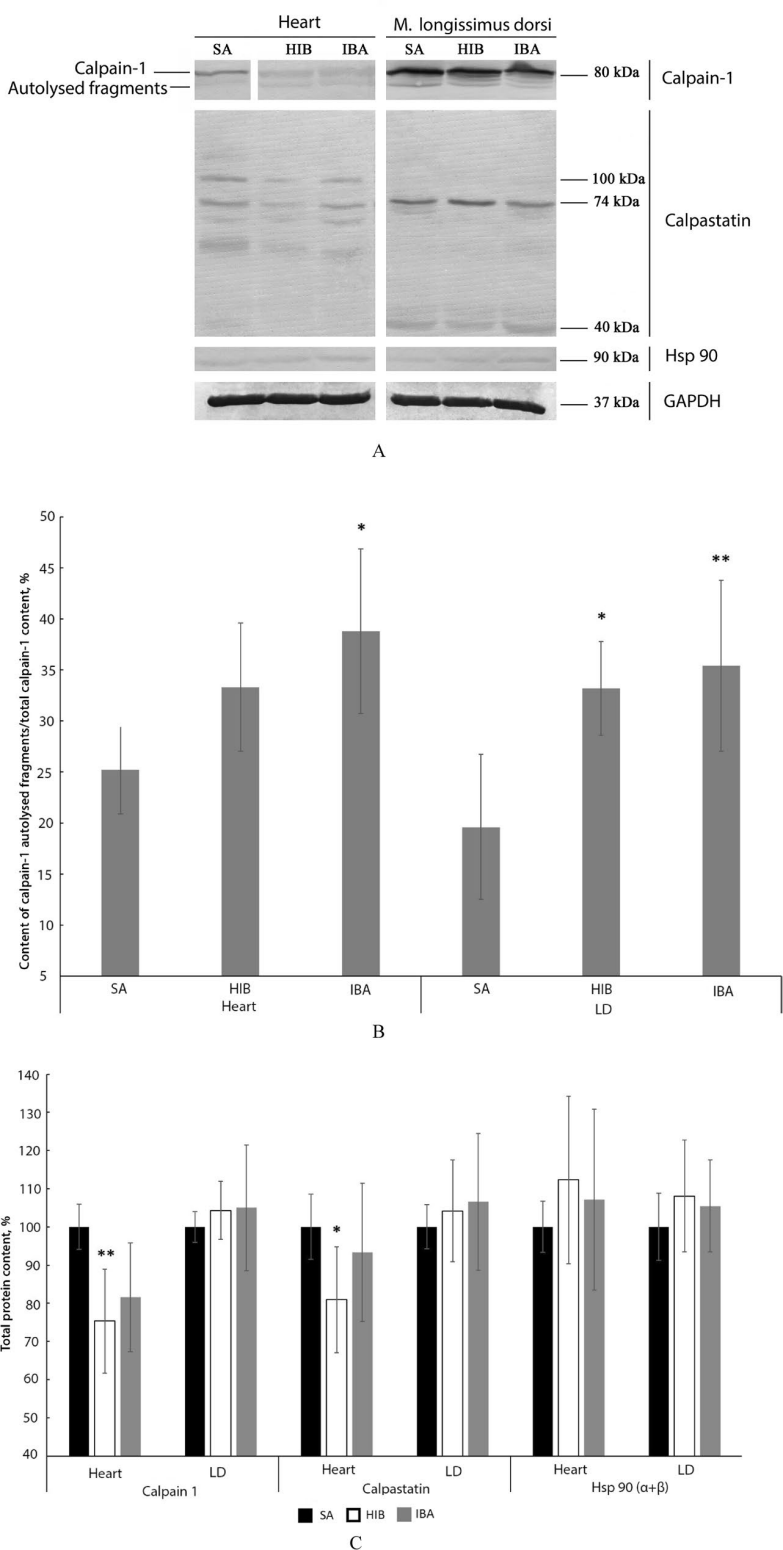
Changes in the contents of calpain-1, calpastatin and Hsp 90 in the hearts and m. longissimus dorsi of ground squirrels. *SA* summer activity, *HIB* hibernation, *IBA* interbout arousal. (**A**) Representative immunoblots of the proteins (contrast of all images, + 100; brightness, from – 18 to – 105). Full-length blot for cardiac calpain-1 is given in Supplementary Figs. S3, S4. The first (SA), third (HIB) and fourth (IBA) tracks from the full-length blot (Supplementary Fig. S3) are shown in (**A**). Full-length blots for other proteins are in Supplementary Figs. S5, S6, S8–S13. The original images are at: https://drive.google.com/open?id=1XljyCN3sfWktjuHRPACab3eQfOc6i2AC. (**B**) Content of autolysed fragments of calpain-1 (as percentage of the total content of calpain-1). Increased content of autolysed calpain-1 fragments was found both in the heart (1.32- and 1.54-fold, *p* < 0.05) and m. longissimus dorsi (LD; 1.69-fold, *p* < 0.05 and 1.8-fold, *p* < 0.01) during hibernation and interbout arousal, respectively (Supplementary Tables S11, S13). (**C**) Bar graphs of the total contents of calpain-1, calpastatin and Hsp90α/ß (Supplementary Tables S10, S12, S14–S17). The statistical analysis of the results was carried out with SigmaPlot 11.0, from Systat Software, Inc., San Jose California USA, https://www.systatsoftware.com. The data were analysed using nonparametric single-factor dispersion analysis for repeated measurements (Kruskal–Wallis One Way Analysis of Variance on Ranks) followed with the pairwise comparison by the Tukey’s test. Values are means ± SD. *p < 0.05, **p < 0.01 (vs SA). *n* = 7/group.

An increase in the content of autolysed calpain-1 fragments was found both in the heart (1.32- and 1.54-fold, *p* < 0.05) and m. longissimus dorsi (1.69-fold, *p* < 0.05 and 1.8-fold, *p* < 0.01) during hibernation and interbout arousal, respectively (Fig. 2; Supplementary Tables S11, S13). A decrease in the total calpain-1 content was detected in the heart during torpor (by 24.7%, *p* < 0.01) (Fig. 2, Supplementary Table S10). Only a tendency to a decrease in the calpain-1 content (by 18.4%) was observed in the hearts of IBA ground squirrels (Fig. 2). The total calpain-1 contents in m. longissimus dorsi of ground squirrels from the three experimental groups were equal (Fig. 2, Supplementary Table S12).

A 19.1% (*p* < 0.05) decrease in calpastatin content was found in the heart during torpor (Fig. 2, Supplementary Table S14). No statistically significant differences were revealed in calpastatin content in the heart of ground squirrels from the IBA and SA groups (Fig. 2). No seasonal differences were observed in the calpastatin contents in m. longissimus dorsi or in the Hsp 90α/ß content in the heart and m. longissimus dorsi of ground squirrels from the three experimental groups (Fig. 2, Supplementary Tables S15–S17).

### SDS–PAGE analysis of titin and nebulin contents

**Figure 3:**
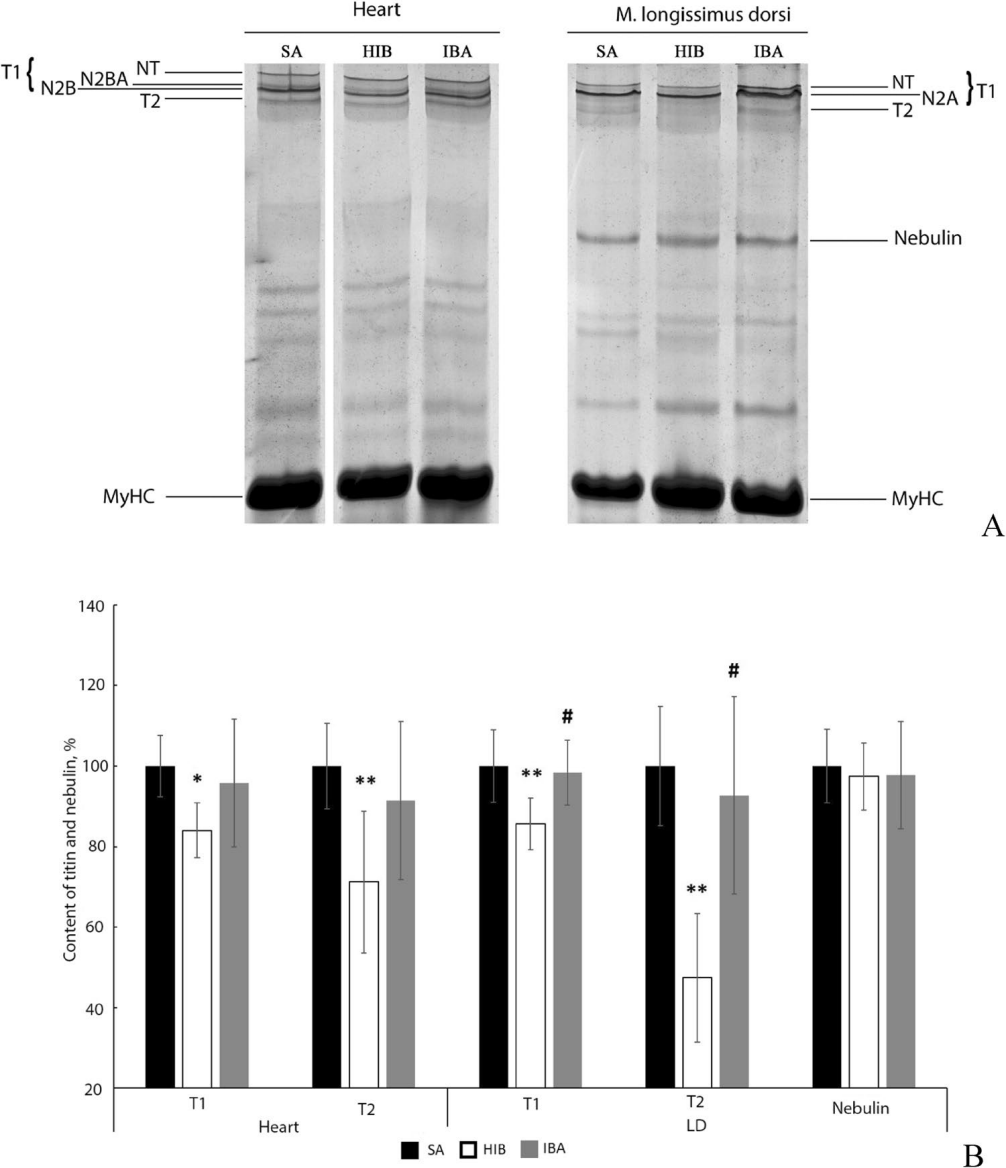
Titin and nebulin contents in striated muscles of ground squirrels. *SA* summer activity, *HIB* hibernation, *IBA* interbout arousal. (**A**) SDS–PAGE analysis of titin in the heart (left) and titin and nebulin in the m. longissimus dorsi (LD, right) (brightness, – 9; contrast, + 30). Full-length gel is shown in Supplementary Fig. S14. The second (SA heart), fourth (HIB heart), fifth (IBA heart), seventh (SA LD), eighth (HIB LD) and ninth (IBA LD) tracks from the full-length gel are shown in (**A**). *MyHC* myosin heavy chains (MW, 205 kDa). T2 (MW, 2,000–2,200 kDa) are proteolytic fragments of titin. NT, N2A, N2BA and N2B are isoforms of intact titin-1 (T1; MW, 3,000–3,700 kDa). High molecular weight titin isoforms (denoted as NT-titin) were found in striated muscles of mammals^63^. (**B**) Bar graphs of T1, T2 fragments and nebulin contents in striated muscles (Supplementary Tables S18–S22). The statistical analysis of the results was carried out with SigmaPlot 11.0, from Systat Software, Inc., San Jose California USA, https://www.systatsoftware.com. The data were analysed using nonparametric single-factor dispersion analysis for repeated measurements (Kruskal–Wallis One Way Analysis of Variance on Ranks) followed with the pairwise comparison by the Tukey’s test. Values are means ± SD. *n* = 7/group. *p < 0.05, **p < 0.01 (vs SA). ^#^*p* < 0.05 (IBA vs HIB).

The content of intact titin-1 (T1) decreased in the heart (by 16%, *p* < 0.05) and m. longissimus dorsi (by 14.4%, *p* < 0.01) during torpor (Fig. 3; Supplementary Tables S18, S20). The content of proteolytic fragments of titin (T2) also decreased in the muscles of HIB animals (by 28.8% and by 2.1 times, respectively, *p* < 0.01) (Fig. 3; Supplementary Tables S19, S21). No reliable differences were found between T1 and T2 contents in striated muscles of ground squirrels from the SA and IBA groups (Fig. 3). The nebulin content was unchanged in m. longissimus dorsi of ground squirrels from the three experimental groups (Fig. 3, Supplementary Table S22).

### Determination of titin phosphorylation level

**Figure 4:**
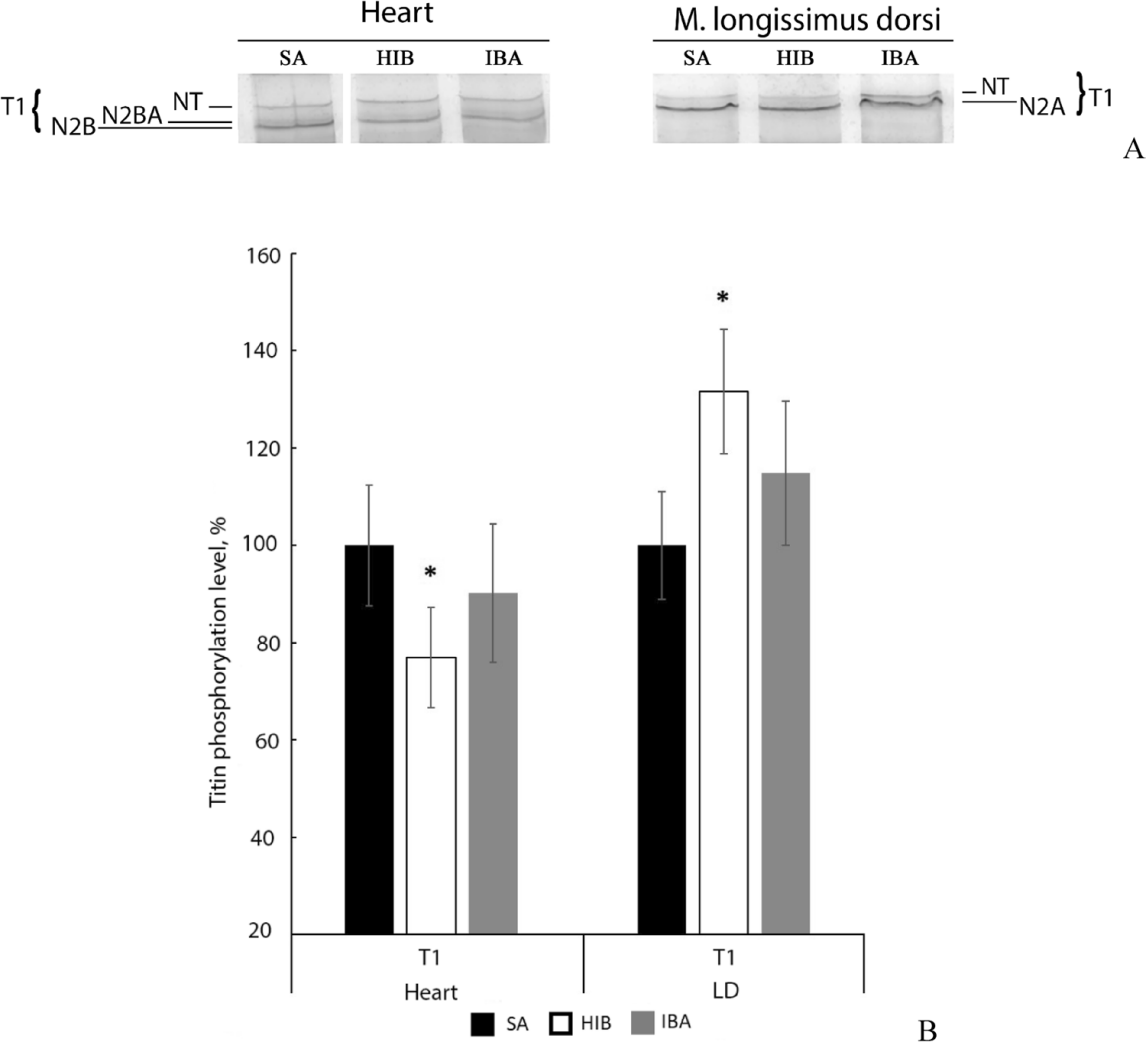
Changes in titin phosphorylation levels in striated muscles of ground squirrels. *SA* summer activity, *HIB* hibernation, *IBA* interbout arousal. (**A**) Heart (left), m. longissimus dorsi (LD, right). The native level of protein phosphorylation was estimated in gels using Pro-Q Diamond fluorescent dye (ThermoFisher Scientific) (brightness, – 41; contrast, – 50). Full-length gel is shown in Supplementary Fig. S15. The second (SA heart), fourth (HIB heart), fifth (IBA heart), seventh (SA LD), eighth (HIB LD) and ninth (IBA LD) tracks from the full-length gel are shown in (**A**). (**B**) Bar graphs of titin phosphorylation level in striated muscles (Supplementary Tables S23, S24). The statistical analysis of the results was carried out with SigmaPlot 11.0, from Systat Software, Inc., San Jose California USA, https://www.systatsoftware.com. The data were analysed using nonparametric single-factor dispersion analysis for repeated measurements (Kruskal–Wallis One Way Analysis of Variance on Ranks) followed with the pairwise comparison using the Tukey’s test. Values are means ± SD. *n* = 5/group. *p < 0.05 (vs SA).

We observed a decreased T1 phosphorylation level (by 23%, *p* < 0.05) in the heart during hibernation (Fig. 4, Supplementary Table S23). An increased T1 phosphorylation level (by 31.6%, *p* < 0.05) was observed in m. longissimus dorsi during torpor (Fig. 4, Supplementary Table S24). No reliable differences were found between T1 phosphorylation levels in striated muscles of summer active animals and ground squirrels during interbout arousal (Fig. 4). Since the T2 content was comparatively low during hibernation and its phosphorylation in striated muscles of ground squirrels was insignificant, seasonal changes in the phosphorylation level of T2 were not compared.

### Electron microscopy of sarcomeric structure

**Figure 5:**
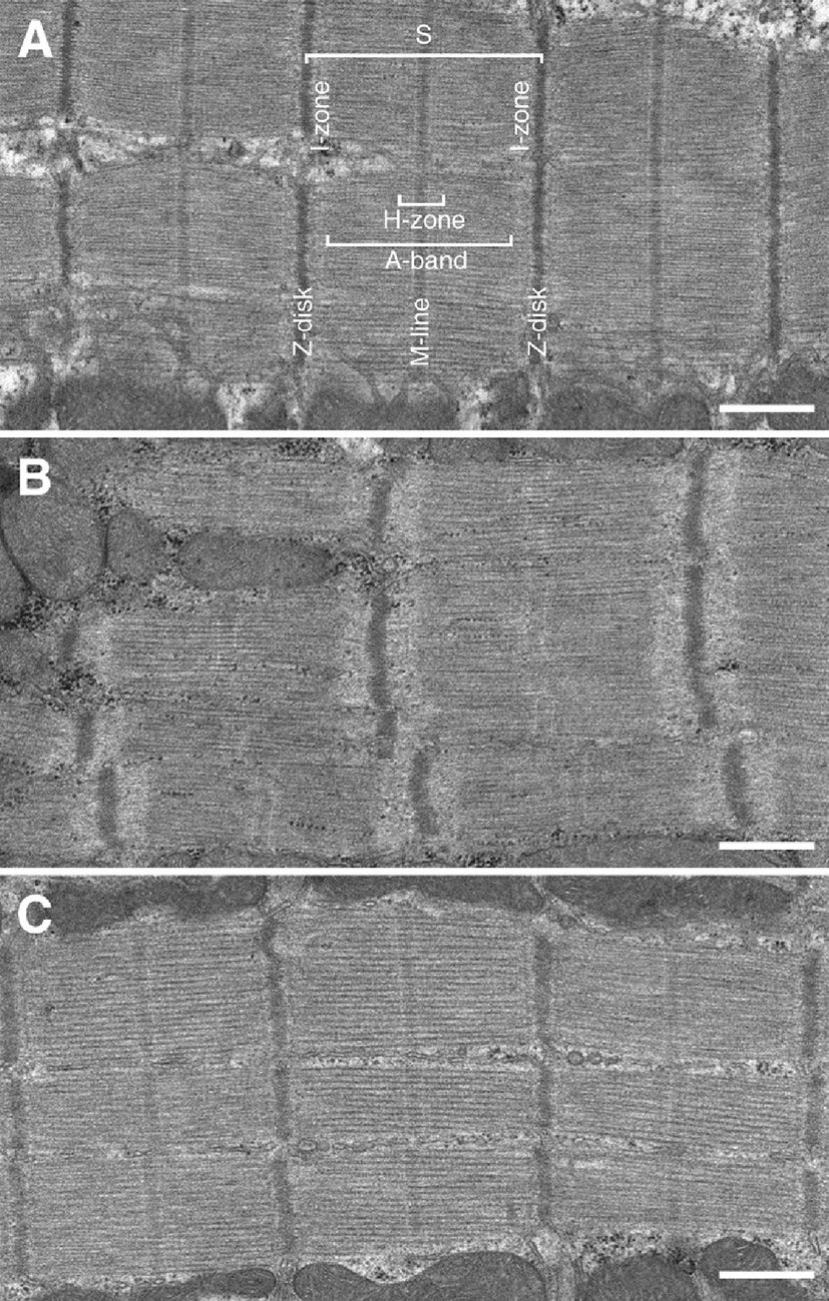
Ultrastructural organization of longitudinally sectioned cardiac muscle sarcomeres (S) of ground squirrels: representative views. (**A**) Summer activity, (**B**) hibernation, (**C**) interbout arousal. All the states manifest similar straight ordered sarcomeric structures (having Z-disks, M-lines; A-, H-, I-zones) with tight bundles of parallel lining myofilaments. All scale bars, 500 nm.

**Figure 6:**
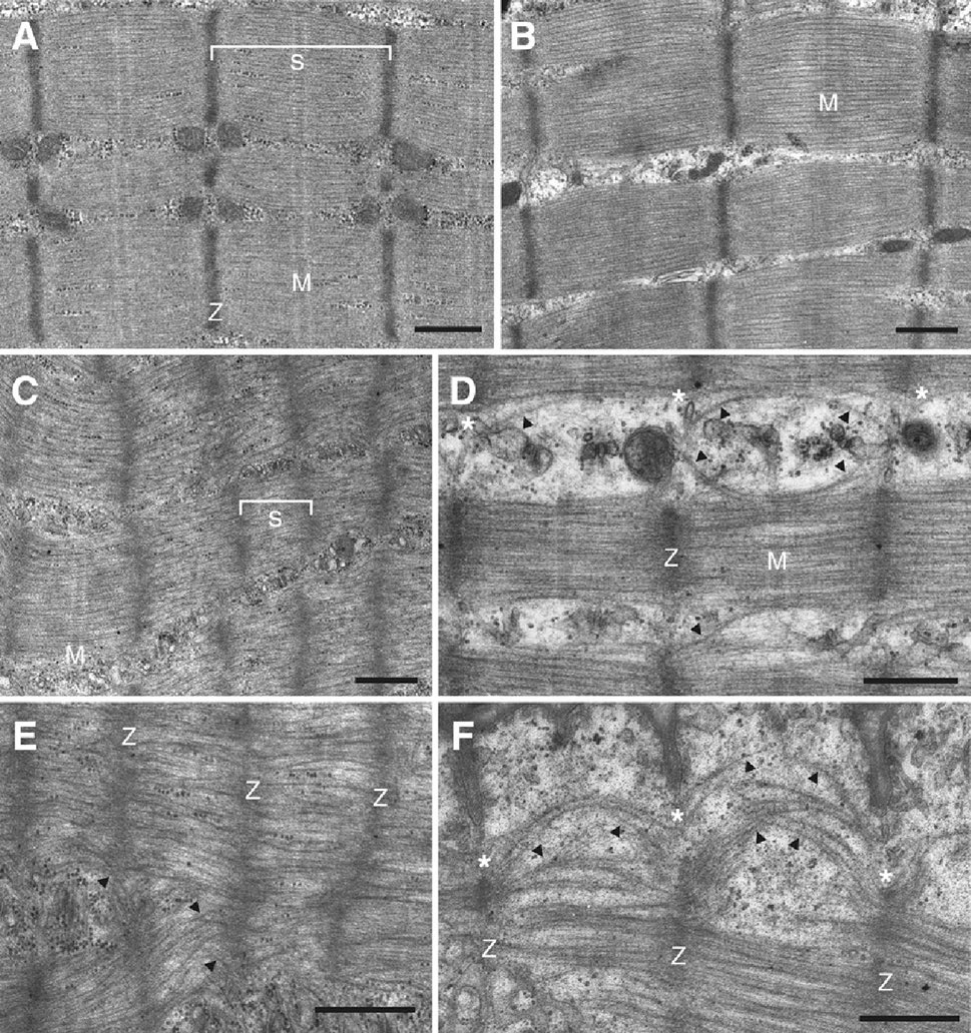
Ultrastructural organization of longitudinally sectioned m. longissimus dorsi sarcomeres of ground squirrels: representative views. (**A**) Summer activity, (**B**) interbout arousal, (**C–F**) hibernation. *S* sarcomeres. About one third of muscle fibres of m. longissimus dorsi during torpor represents straight ordered sarcomeric structures. The other two thirds show a disordered sarcomeric structure, with dramatic changes in sarcomeric length (cf. length S on **A** and **C**), disordering of myofilament directions (arrowheads on **E**), disappearance of zones I, A, H and lines M (see **C**,**E**,**F**), dissociation of Z-disk structure (Z on **E**,**F**), and detachment of myofilaments from Z-disks (asterisks on **D**,**F**) and from one another (arrowheads on **D**,**F**). (**A**,**B**,**C**,**E**) are the central part of the myofibre; (**D**,**F**), in close vicinity to myofibres’ plasma membrane. All scale bars, 500 nm.

We observed no changes in the sarcomeric structure of the heart from the three experimental groups (Fig. 5). Disorders of the sarcomeric structure were found in m. longissimus dorsi during hibernation (Fig. 6C–F). About one third of m. longissimus dorsi muscle fibres during torpor were represented by straight ordered sarcomeres. Two thirds of the muscle fibres exhibited disordered sarcomeric structures with dramatic changes in sarcomeric length (cf. length S in Fig. 6A,C), disordering of myofilament directions (arrowheads in Fig. 6E), disappearance of I-, A-, H-zones and M-lines (see Fig. 6C,E,F), dissociation of Z-disk structure (Z in Fig. 6E,F), and detachment of myofilaments from Z-disks (asterisks in Fig. 6D,F) and from one another (arrowheads in Fig. 6D,F). No sarcomeric structure disorders in m. longissimus dorsi of IBA ground squirrels were found.

### Protein synthesis rate

**Figure 7:**
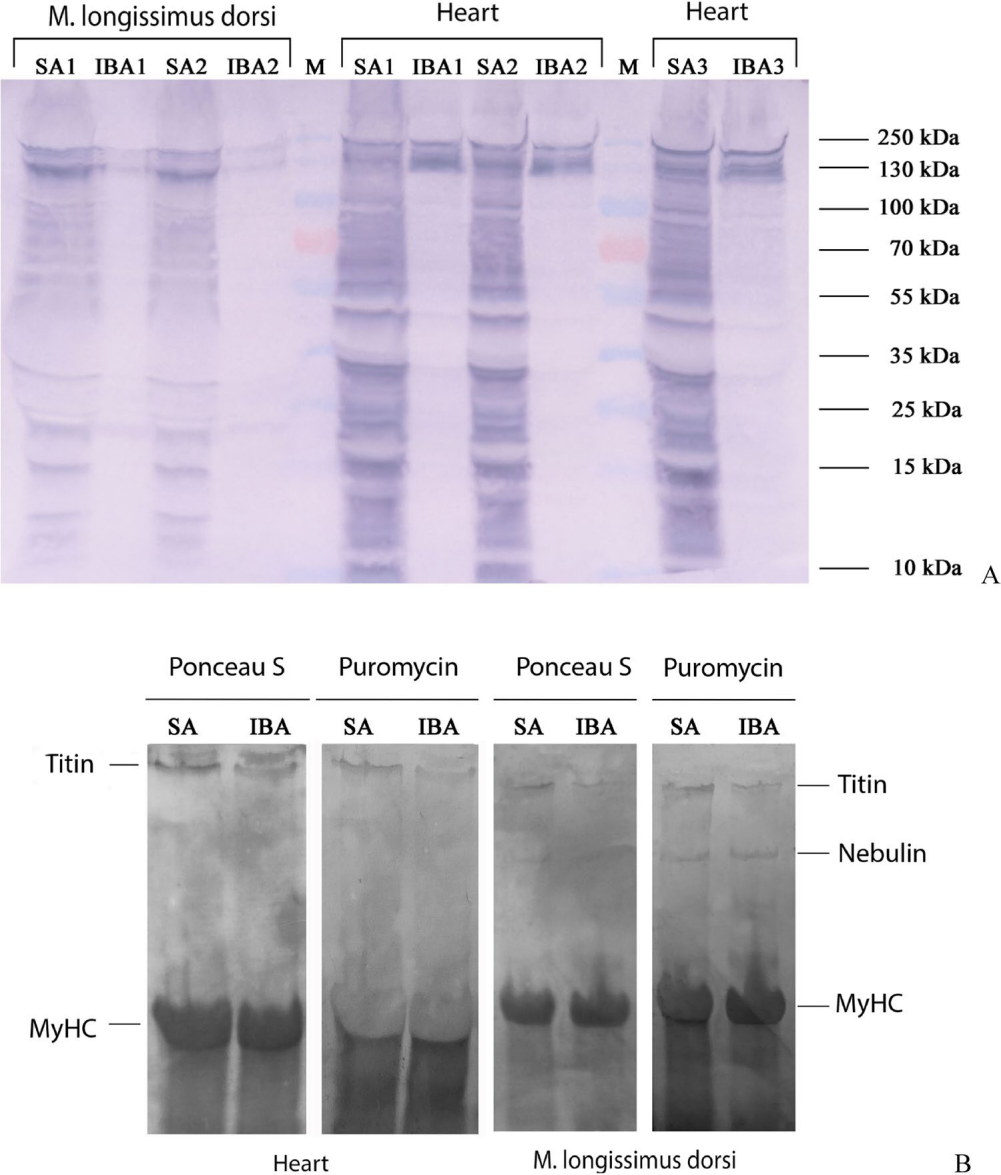

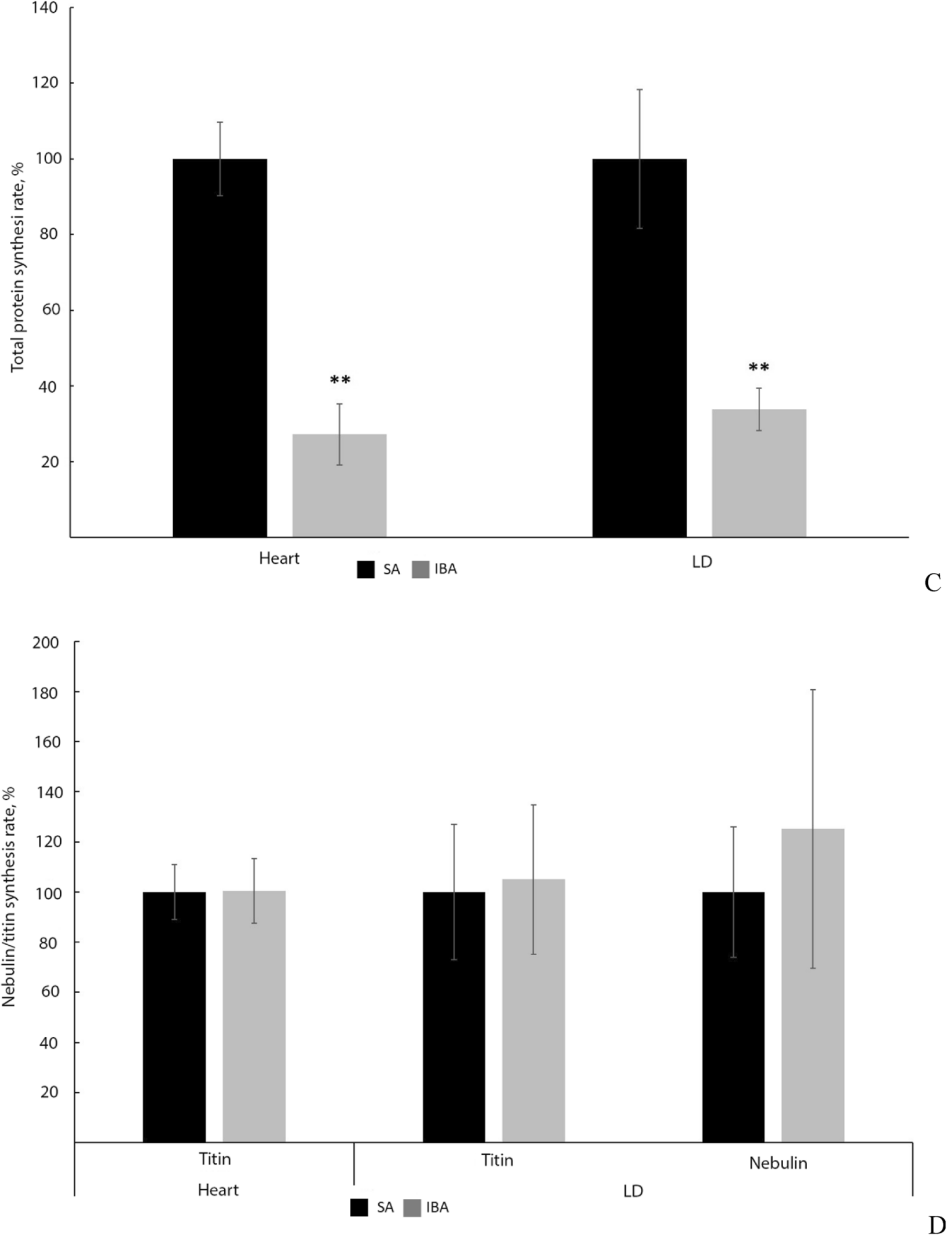
Rate of protein synthesis in cardiac and skeletal muscles of ground squirrels. *SA* summer activity, *IBA* interbout arousal. (**A**) Total protein synthesis. Full-length representative immunoblot for puromycin on PVDF membrane (brightness, + 30; contrast, – 50). The levels of proteins synthesised in vivo were identified relative to the total protein level. The total protein level in the samples was measured by the Bradford method. Bovine serum albumin was used as a standard. M, molecular weight marker. The original images are at: https://drive.google.com/open?id=1XljyCN3sfWktjuHRPACab3eQfOc6i2AC. (**B**) Syntheses of nebulin and titin. Ponceau S staining and representative immunoblots for puromycin on nitrocellulose membranes (puromycin for heart: brightness, – 88; contrast, + 200; puromycin for m. longissimus dorsi: brightness, – 90; contrast, + 200; Ponceau S for heart and m. longissimus dorsi, without changes). The levels of proteins synthesised in vivo were identified relative to the total protein level (stained with Ponceau S). SDS-PAGE analysis was performed using agarose-strengthened 2.2% polyacrylamide gel. MyHC, myosin heavy chains. Full-length Ponceau S and puromycin stained membranes are shown in Supplementary Figs. S16–S23. The first and second tracks (heart, Supplementary Figs. S16, S18), the second and third tracks (m. longissimus dorsi, Supplementary Figs. S20, S22) from the full-length membranes are shown in (**B**). (**C**) Bar graphs of total protein synthesis in the heart and m. longissimus dorsi (Supplementary Tables S25, S26). There was a significant decrease in the rate of total protein synthesis in the heart (by 3.67, *p* < 0.01) and m. longissimus dorsi (by 2.96, *p* < 0.01) during interbout arousal in comparison with those in summer active animals (*n* = 5/group). (**D**) Bar graphs of titin and nebulin syntheses in striated muscles (Supplementary Tables S27–S29). No significant differences were found between the synthesis rates of titin and nebulin in muscles of ground squirrels from the two groups (*n* = 3/group). The data were analysed using the nonparametric Mann–Whitney U criterion. Values are means ± SD. **p < 0.01.

SUnSET measurements revealed a significant decrease in the rate of total protein synthesis in the heart (by 3.67 ± 0.8, *p* < 0.01) and m. longissimus dorsi (by 2.96, *p* < 0.01) during interbout arousal as compared to that in summer active animals (Fig. 7A,C; Supplementary Tables S25, S26). No reliable differences were found in the synthesis rates of titin and nebulin in ground squirrel muscles from the SA and IBA groups (Fig. 7B,D; Supplementary Tables S27–S29).

## Discussion

Seasonal changes in the total content of calpain proteases (calpain-1, calpain-2) and their inhibitor calpastatin in striated muscles of such hibernators as the Daurian ground squirrel^7,22,36^ and thirteen-lined ground squirrel^30,31^ have been investigated earlier. Various changes have been found in the content of these proteins in different torpor–activity cycles in striated muscles of these hibernators. This research is the first to report on seasonal changes in the total content of calpain-1 and calpastatin in striated muscles of the long-tailed ground squirrel *Urocitellus undulatus*. No seasonal differences were observed in calpain-1 and calpastatin contents in m. longissimus dorsi of animals from the three experimental groups (Fig. 2). A comparatively minor but statistically significant decrease in the content of these proteins was found in the heart of HIB ground squirrels (Fig. 2).

This article is also the first to communicate an increased autolysis of calpain-1 in striated muscles of a hibernating animal during interbout arousal (Fig. 2). These data are indicative of a hyperactivation of calpain-1 in the heart and m. longissimus dorsi of the long-tailed ground squirrel during this period. These changes were accompanied by the recovery of T1 content, which was reduced in striated muscles during hibernation (Fig. 3). Simultaneously, the content of titin T2 fragments significantly increased in striated muscles in the IBA group as compared to the HIB group (Fig. 3). These results testify to an increased titin turnover in striated muscles during interbout arousal. The increase can be explained by the hyperactivation of calpain-1 because titin is substrate for this protease. Our SUnSET data are consistent with these results. A significant decrease was found in the rate of total protein synthesis (MW range, 10–200 kDa) in the heart and m. longissimus dorsi during interbout arousal, whereas no reliable differences were observed between the synthesis rates of titin and nebulin in the muscles of animals from the IBA and SA groups (Fig. 7). These changes were accompanied by the restoration of the highly ordered sarcomeric structure of m. longissimus dorsi (Fig. 6B), by a slight increase in the volume of muscle fibres of m. longissimus dorsi (Fig. 1) and by a trend towards an increase in the mass of the heart muscle in the IBA group (Table 1). Titin and nebulin are giant proteins of thick and thin filaments and play the role of a sarcomeric cytoskeleton. The renewal of these proteins and the recovery of their content during interbout arousal shall, undoubtedly, contribute to an increase in muscle mass.

Thus, our results show that the hyperactivation of calcium-activated protease calpain-1 in striated muscles of long-tailed ground squirrels during interbout arousals is accompanied by a recovery/renewal of the titin and nebulin contents and an increase in muscle mass. Data on Ca^2+^ overload during interbout arousals in three skeletal muscles of Daurian ground squirrels^35^ are consistent with our findings. An increased activity of calpain-1 may contribute to an activation of the NFAT-calcineurin pathway^30,31^, as well as to an increase in myofibrillar protein turnover, which is initiated by calpain-dependent proteolysis of titin and nebulin. These changes, in turn, may contribute to muscle mass retention during hibernation. The results obtained do not contradict the conclusion made by the authors of Ref.^33^ that the myofibrillar remodelling is most likely responsible for preventing skeletal muscle atrophy during a prolonged disuse in hibernation.

An increased content of autolysed fragments of calpain-1 was also observed in the heart and longissimus dorsi muscle of long-tailed ground squirrels during torpor (Fig. 2). There is no doubt that in hypothermia, when the muscle temperature is 1.5–2.5 °C, the activity of calpain-1 is considerably but, apparently, not completely, inhibited. The level of titin, especially its T2 fragments, reduced in muscles of long-tailed ground squirrels during torpor (Fig. 3). Similar changes were found in striated muscles of the brown bear (*Ursus arctos*) during winter sleep^52^. These results show that the proteolytic activity of calpains in striated muscles of hibernating animals is maintained during hibernation. These data are consistent with the results of our earlier research using casein zymography^53^. The ability of calpain-1 (μ-calpain) and calpain-2 (m-calpain) to proteolyse casein in gel was significantly higher in extracts prepared from striated muscles of long-tailed ground squirrels from the HIB group as compared to the SA group^53^. Incomplete inhibition of calpain proteolytic activity may be due to Ca^2+^ overload observed, for instance, in the gastrocnemius muscle of Daurian ground squirrels during hibernation^35^. The authors of Ref.^35^ have hypothesised that Ca^2+^ overload may activate calpains and promote protein degradation during hibernation. In this context, it is necessary to discuss our data related to changes in the titin phosphorylation level in the heart and longissimus dorsi muscle of the long-tailed ground squirrel.

It is known that phosphorylation modifies the sensitivity of proteins to degradation by calpain-1^54^. We found no direct experimental evidence to confirm a change in the sensitivity of titin to proteolysis mediated by a change in its phosphorylation level. However, there are indirect data testifying that hyperphosphorylation of skeletal muscle titin enhances its sensitivity to cleavage by calpains (for references, see Ref.^55^). Our data obtained in this work do not contradict this assumption. A decreased titin content was observed in longissimus dorsi muscle during hibernation against the background of the increased phosphorylation level of T1 (Figs. 3, 4). The hyperphosphorylation of skeletal muscle titin can be mediated by calcium-dependent protein kinases such as PKC, the activity of which should increase under Ca^2+^ overload identified in striated muscles of Daurian ground squirrels during torpor^35^. A decrease in the T1 content in the skeletal muscle of long-tailed ground squirrels from the HIB group probably contributed to sarcomeric structure disorders (Fig. 6), because it is known that giant elastic protein titin (also known as connectin) plays an important role in maintaining an ordered sarcomeric structure^56,57^. An increase in the number of PO_3_^-^ groups in titin could also contribute to that of interfilament spacing within this muscle (Fig. 6D–F). These changes may increase the access of proteases to the most vulnerable parts of the titin molecule.

A decreased phosphorylation of titin was detected in the cardiac muscle of long-tailed ground squirrels during torpor (Fig. 4). The molecular mechanisms of these changes are not clear, but hypophosphorylation of titin might probably be responsible for the decrease in its sensitivity to proteolysis in cardiac muscle, the contractile activity of which is not completely suppressed during hibernation. This hypothesis needs to be proven.

When discussing the results, the point to note is that there were no appreciable seasonal changes in calpastatin and Hsp 90 levels (Fig. 2). It was shown that Hsp 90 interacted with calpain-1, not with calpain-2, to form a discrete complex where the protease maintains its catalytic activity, though with a lower affinity for Ca^2+^ ions^51^. Keeping in mind that the level of Hsp 70 was 1.7 times higher during hibernation and the early phase of arousal in skeletal muscles of the hibernating bat *Murina leucogaster*^58^, we expected to observe similar changes for Hsp 90, which in turn would have led to an increased activity of calpain-1. This assumption was not confirmed. No significant seasonal differences were found in the level of Hsp 90 in investigated striated muscles of long-tailed ground squirrels (Fig. 2). Our results provide evidence that calpain-1 is the most essential of the investigated proteins for turnover and de novo recovery of giant proteins of thick and thin filaments during interbout arousals.

In conclusion, the results of our study indicate that increased autolysis of calpain-1 was accompanied by predominant synthesis of giant myofibrillar proteins titin and nebulin with significant inhibition of synthesis of other proteins in striated muscles of long-tailed ground squirrels during interbout arousals. These changes were accompanied by a slight increase in the volume of muscle fibres of m. longissimus dorsi, by a trend towards an increase in heart muscle mass and recovery of a highly ordered sarcomeric structure of m. longissimus dorsi in the IBA group. Myofibrillar remodelling most likely plays an important role in long-tailed ground squirrels both in preventing skeletal muscle atrophy and in cardiac muscle mass retention during hibernation. However, it is to be verified if such changes would be observed in other skeletal muscles, e.g., hind-limb muscles, in this ground squirrel species and in other hibernators. It may be assumed that both atrophic changes and those of various molecular parameters can differ significantly depending on the type of muscles containing—to a greater or lesser extent—slow- or fast-twitch fibres. In this regard, it looks promising to compare two traditionally investigated skeletal muscles—slow-twitch m. soleus and fast-twitch m. gastrocnemius.

## Materials and methods

### Experimental animals

Long-tailed ground squirrels *Urocitellus undulatus* of both sexes (body mass, 450–750 g; estimated age, between 1 and 2 years old) were captured in early August 2017 and early August 2018 in Yakutiya (Siberia), transported by air to Pushchino (Moscow Region) and housed in individual cages (74 × 57 × 55 cm) in a specially equipped vivarium under natural photoperiodicity. Food was supplemented with sunflower seeds and carrots, and nesting material was provided ad libitum. Early in November, the animals were weighed, then the cages with the animals were transferred to a darkroom with a temperature of 1–3 °C for the onset of the hibernation season. The weights of the animals were 535–845 g (Supplementary Table S5). Part of the animals was individually placed in wooden hibernation boxes (20 × 20 × 25 cm). At the bottom of the wooden animal box, a thermistor (sensitivity, 0.2 °C) was mounted in the nest bedding. During hibernation, the temperature of the bedding was in the range of 1–4 °C, whereas in interbout events it increased to 14 °C. The course of torpor–activity cycles could thus be monitored in individual animals and enabled predicting the length of a subsequent bout.

In December, hibernation bouts were 7–8 days; in January–February, 10–14 days. In March, hibernation bouts lasted for 8–10 days. Food was not provided during hibernation. “Deep torpor” samples were taken from animals sacrificed in the predicted mid-bout. Spontaneously aroused animals were placed into individual cages, which contained nesting material and ~ 100 g of cabbage as a source of moisture. After several hours these animals were sacrificed for taking samples. As wooden hibernation boxes became freed, they got occupied by other ground squirrels, which were preliminarily weighed.

Experiments were carried out with three groups of animals taken at different phases of their annual cycle: (1) hibernation (HIB; hypothermia, myocardium temperature, 2.3 °C ± 0.1 °C; rectal temperature, 1.5 °C ± 0.1 °C; duration of hypothermia, 4–11 days, January–February 2018, *n* = 3; the last 10 days of December 2018, *n* = 1; January–February 2019, *n* = 2; the first 10 days of March 2019, *n* = 1); (2) interbout arousal (IBA; normothermia, 36–37 °C, winter activity, during the first 5–12 h after the spontaneous arousing, January–February 2018/2019, *n* = 7); (3) summer activity (SA; normothermia, 38 °C, June–July 2018/2019, *n* = 7). Samples of myocardium of the left ventricle and of m. longissimus dorsi (m. longissimus lumborum) taken from the lumbar region of the spine were rapidly excised and frozen in liquid nitrogen. The hearts of ground squirrels were preliminarily weighed. Muscle tissue samples were stored at – 75 °C until further use. For microscopy, muscle tissue samples were submerged into a fixing solution as indicated below.

All animal procedures performed with ground squirrels were approved by the Commission on Biosafety and Bioethics (Institute of Cell Biophysics—Pushchino Scientific Center for Biological Research of the Russian Academy of Sciences, Permission no. 6 of December 12, 2017) in accordance with Directive 2010/63/EU of the European Parliament. Surgeries for active ground squirrels were performed under anaesthesia with Zoletil (Virbac Sante Animale, Carros, France) (4 mg/kg, i.m.); all efforts were made to minimise animal suffering.

### SDS–PAGE analysis and Western blotting

Extraction of calpain-1 from muscle tissues was based on a previously described method^59^. Muscle tissues were homogenised in lysis buffer (Tris buffer) containing 0.4 M Tris HCl, pH 6.8, and 25 mM EGTA. Following homogenization, 4% SDS was added to the buffer. The homogenates were then incubated at 4 °C for 20–40 min and centrifuged at 3,000×*g* for 5 min. Subsequently, the supernatant was collected and mixed (2:1 v/v) with SDS loading buffer (0.125 M Tris HCl, 10% glycerol, 4% SDS, 4 M urea, 10% ß-mercaptoethanol and 0.001% bromophenol blue, pH 6.8). The samples were heated at 95 °C for 4 min. Calpastatin, Hsp 90 and GAPDH (reference protein, Table 2) were extracted from muscles using lysis buffer (12 mM Tris HCl, 1.2% SDS, 5 mM EGTA, 10% glycerol, 2% ß-mercaptoethanol, 5 µg/ml leupeptin and E64, pH 6.8–7.0). Total protein concentrations in the samples were measured by the Bradford method according to the manufacturer’s recommended protocol (Sileks, Russia). Bovine serum albumin was used as a standard. The protein samples were electrophoresed in 6.5% (for calpain-1) and 9.5% (for the other above-mentioned proteins) polyacrylamide slab gels^60^. Equal protein amounts were added to the gels (muscle samples of all experimental groups were run on the same gel).

**Table 2:** GAPDH levels in the heart and m. longissimus dorsi of ground squirrels (Supplementary Tables S6, S7).

Seasonal period	Heart, %, *n* = 6	m. longissimus dorsi, %, *n* = 7
SA	100.0 ± 5.0	100.0 ± 3.7
HIB	99.2 ± 6.6	99.3 ± 3.1
IBA	104.3 ± 12.7	100.9 ± 2.7

Values are means ± SD.

*SA* summer activity, *HIB* hibernation, *IBA* interbout arousal.

Protein transfer to PVDF or nitrocellulose membranes was run for 2 h at 100 mA according to a previously described method^61^. Titin and nebulin were transferred for three days at 80 mA. To verify the equal loading of protein in all lanes, the nitrocellulose membrane was stained with Ponceau S. The membranes were blocked for 1 h at room temperature with blocking buffer (5% nonfat milk powder; PBS, pH 7.4; and 0.05% Tween 20). Then the membranes were washed in buffer (PBS, pH 7.4; and 0.05% Tween 20) 5 times for 5 min and were incubated for 2 h at 21–23 °C with primary rabbit monoclonal antibodies against calpain-1 (1:4,000; Abcam, ab28258; Cambridge, UK), calpastatin (1:3,000; Abcam, ab28252), GAPDH (1:2000; Abcam, ab37168), with primary mouse monoclonal antibodies against puromycin (1:2000, Kerafast, Anti-Pyromycin [3RH11]) and against Hsp 90α/ß (1:2000, clone number 6H1/F8, produced at the laboratory headed by Prof. O.S. Morenkov, ICB RAS, see information about the antibodies in Supplementary Materials (Supplementary Fig. S24, pp. 50–51). Secondary antibodies conjugated to alkaline phosphatase (goat anti-rabbit Ig, 1:3,000; Abcam, ab6722 and goat anti-mouse Ig, 1:3,000; Abcam, ab6790) were used. Incubation with secondary antibodies proceeded at 21–23 °C for 1 h. Then the membranes were washed in buffer (PBS, pH 7.4; and 0.05% Tween 20) 5 times for 5 min. An NBT/BCIP substrate solution (Roche, Basel, Switzerland) was used to visualise the antibody–protein complexes. Incubation with this substrate was carried out at room temperature for 5–30 min. The GAPDH protein contents were used as loading controls. All stages of incubation of PVDF or nitrocellulose membranes were carried out on an MR-1 Mini-Rocker Shaker (Biosan, Latvia).

Changes in titin-1 (T1) isoform (3,000–3,700 kDa), T2 fragment (T2, 2000–2,200 kDa) and nebulin (700 kDa) contents were detected using polyacrylamide slab gels (2.2%). The gels were strengthened with agarose according to the Tatsumi–Hattori technique^62^ with our modifications^63^. Muscle tissues were homogenised in lysis buffer (12 mM Tris HCl, 1.2% SDS, 5 mM EGTA, 10% glycerol, 2% ß-mercaptoethanol or 75 mM DTT, 5 µg/ml leupeptin and E64, pH 6.8–7.0). To prevent titin degradation at high temperatures^64^, the samples were incubated for 30–40 min at + 40 °C instead of boiling^63^. The samples were mixed on an ELMI V-3 shaker (Latvia) every 5 min. SDS-PAGE analysis was performed using the Helicon system (Moscow, Russia, size of slab gel 10.0 × 8.0 × 0.1 cm) at 8–10 mA. The gels were stained with Coomassie Brilliant Blue G-250 and R-250 mixed at a 1:1 ratio. Western blotting was carried out using the Bio-Rad Mini Trans-Blot System (Bio-Rad Laboratories, Inc.).

### Determination of titin phosphorylation level

The level of titin phosphorylation was determined using a previously described method^65^ with minor modifications. The native level of protein phosphorylation was estimated in the gels using a Pro-Q Diamond fluorescent dye (ThermoFisher Scientific). The gels were incubated in an aqueous solution of 50% EtOH and 10% acetic acid for 12–18 h, washed with distilled water for 30 min and stained for 1.5 h. The gels were then washed with Pro-Q Diamond phosphoprotein gel destaining solution (ThermoFisher Scientific), and protein bands containing phosphate groups were visualised using a Bio-Rad ChemiDoc Touch Imaging System (Bio-Rad Laboratories, Inc., Hercules, CA, USA). Finally, the gels were stained with Coomassie Brilliant Blue G-250 and R-250 mixed at a 1:1 ratio to estimate the total protein content.

### Electron microscopy

For ultrastructural analysis, muscle samples (3–5 mm in length and 1-mm-thick) were dissected from cardiac muscle (from the upper third of left ventricle) and m. longissimus dorsi muscle of ground squirrels from the three experimental groups. After circulatory arrest the time lapse did not exceed 1.5 min before muscles were submerged into a fixing solution containing 3% paraformaldehyde, 1.25% glutaraldehyde and 50 mM sucrose in 0.1 M Na-cacodylate buffer (pH 7.2–7.4). After 2–4 h at room temperature, the fixing solution was replaced with 2.5% glutaraldehyde and 50 mM sucrose in the same buffer and left overnight at + 4 °C. Then the samples were washed twice to remove aldehydes and postfixed at room temperature in 1% osmium tetroxide in the same buffer for 1–2 h. After drying in ethanol and acetone, the tissues were embedded in EMBed epoxy resin (EMbed812/NMA/DDSA; mixture ratio A:B = 1:1 in accordance with^66^), cured for 48 h at + 60 °C and cut to semithin sections to achieve proper longitudinal orientation of muscle sarcomeres. Thin sections were prepared with a Leica EM UM6 ultramicrotome, picked up on pioloform-coated slot grids, stained with 2% uranyl acetate and modified Sato triple lead stain, and imaged with a JEOL 1200EX electron microscope at a magnification of ×12 K. Negatives were digitised at 2,400 dpi on an Epson Perfection V700 photo scanner with SilverFast software. Photoshop software was used to enhance brightness/contrast levels across the entire field of each image. Final magnifications were calculated according to the images of cross grating replicas (2,160 lines/mm; https://www.2spi.com/item/02902-ab/) imaged at the same magnification.

### Determination of muscle fibre volumes by 3D reconstruction

Using 3D reconstruction, we assessed the level of atrophic changes in the m. longissimus dorsi. For 3D reconstruction, fragments of m. longissimus dorsi were taken from animals of the SA (*n* = 5), HIB (*n* = 5) and IBA (*n* = 4) groups. The samples were fixed for 16 h at room temperature by submerging into a solution containing 4% paraformaldehyde, 2.5% glutaraldehyde and 50 mM sucrose in 0.1 M Na-cacodylate buffer (pH 7.4). For post-fixation, a 2% solution of osmium tetroxide was used. After drying in ethanol and acetone, the tissues were embedded in EMBed epoxy resin and cured for 48 h at + 60 °C. Serial 9-µm-thick sections of m. longissimus dorsi were prepared from blocks embedded into epoxy resin on a pyramitome (LKB 11800, Sweden). Imaging was performed on an NU-2E microscope (Carl Zeiss, Germany, E25x Planachromat objective) at 300 × 300 resolution using a NIKON D5100 digital camera and Camera Control Pro 2.11.0 W software (see Supplementary Fig. S1). At least 8–20 consecutive shots of one slice were taken by the checkerboard method. Then a panoramic image of each section was produced using PTGui 9.1.8 Pro; the contrast and brightness of the sections were changed using Adobe Photoshop. The scale of the object micrometer at the same magnification was photographed. The panoramic images of the serial sections were aligned relative to one another in IGL Align sEM Align. Each point marked on the reference image was indicated by a corresponding point on an image to be aligned^67^. In addition, the contours of external perimysium served as reference points. The outlines for each muscle fibre were constructed by manual segmentation in IGL Trace (version 1.20b)^67^. For calibration, the shrinkage coefficient of muscle tissue (5%) induced by postfixation procedures was taken into account (see Supplementary Fig. S2). The 3D images of muscle fibres were generated in the wrl/vrml format followed by their transformation into the raw format. The 3D images of muscle fibres were formed using IGL Trace software (see Supplementary Fig. S2). The quantitative parameters were calculated using the commercial program Actify’s 3D View. The volumes of 300–375 muscle fibres (the number of fibres for each muscle sample was 75) were calculated for each animal group (see Supplementary Tables S8, S9). Since the distribution of the volume data was not normal (Shapiro–Wilk test), we estimated the significance of differences using nonparametric single-factor dispersion analysis for repeated measurements (Kruskal–Wallis One Way Analysis of Variance on Ranks) which was followed with the pairwise comparison by the Tukey’s test. The values are given as M ± SEM or M ± SD, where M is the mean value, SEM is the standard error of the mean and SD is the standard deviation. Statistical significance was set at *p* ≤ 0.05. The dynamics of atrophic change development in the investigated skeletal muscle from December to March was not studied.

### SUnSET technique for measuring the protein synthesis rate

SUnSET (surface sensing of translation) is a nonradioactive technique for in vivo measurement of protein synthesis in striated muscles^68^. The technique involves the use of the antibiotic puromycin (a structural analogue of tyrosyl-tRNA) and anti-puromycin antibodies to detect the amount of puromycin incorporation into nascent peptide chains. The SUnSET technique uses standard Western blotting and immunohistochemical technologies to visualise and quantify in vivo rates of protein synthesis^68–70^. In our experiments for in vivo measurements of protein synthesis, summer active (*n* = 5) and winter active (*n* = 5) ground squirrels were injected intraperitoneally with 40 nmol/g puromycin hydrochloride (Enzo Life Sciences, USA) diluted in normal saline (Solopharm, Russia). Exactly 20 min after the injection, the animals were anaesthetised with Zoletil. Exactly 25 min after the puromycin injection, the anaesthetised ground squirrels were euthanised by decapitation, striated muscle tissue was collected and frozen immediately in liquid nitrogen for Western blotting analysis.

### Densitometry and statistical analysis

Gels and membranes were digitised, and the data were processed using Total Lab v1.11 software (Newcastle Upon Tyne, England). The levels of calpain-1, calpastatin and Hsp 90 were analysed relative to GAPDH (reference protein). Applying the SUnSET assay, the levels of proteins synthesised in vivo were identified relative to the total protein (measured by the Bradford method or stained with Ponceau S). To determine the titin and nebulin contents relative to the MyHC content, the total optical density (OD) of the MyHC peak, as well as the total OD of the nebulin and titin peaks (T1 isoforms (T1) and T2 fragments (T2)), were determined. There is evidence that the titin/myosin ratio within the A-band titin in the sarcomere is 6 titin molecules per half myosin filament^71^. A previously described method for estimating the titin and nebulin contents relative to the MyHC content is widely used^72^. This approach is more precise than that for measuring the titin content relative to the total protein content in a sample. The statistical analysis of the results was carried out with SigmaPlot 11.0, from Systat Software, Inc., San Jose California USA, www.systatsoftware.com. Since the distribution of some data samples was not normal (Shapiro–Wilk test), we estimated the significance of differences using nonparametric single-factor dispersion analysis for repeated measurements (Kruskal–Wallis One Way Analysis of Variance on Ranks) which was followed with the pairwise comparison by the Tukey’s test. Data obtained while measuring the protein synthesis rates in striated muscles of ground squirrels from the SA and IBA groups were analysed using the nonparametric Mann–Whitney U criterion. The values are presented as M ± SD, where M is the mean value and SD is the standard deviation. The differences were considered to be statistically significant at *p* < 0.05.

### Equipment and settings

Gels and blots were digitised at 1,200 dpi on an Epson Perfection 3,200 PHOTO scanner with Epson scan 3.0 software. Photoshop software was used to enhance the brightness/contrast levels across the entire field of each image. Minor manipulations were performed to remove contamination in the figures of the article. Densitometry was performed using Total Lab v1.11 software (Newcastle Upon Tyne, England). The measurement tracks were created manually. Background subtraction was done by the rubber-band tool. Protein bands containing phosphate groups were visualised using a Bio-Rad ChemiDoc Touch Imaging System (Bio-Rad Laboratories, Inc., Hercules, CA, USA). Gels were placed on the Chemi/UV/Stain-Free Tray, and measurements were carried out according to the following parameters: Application Category—Protein Gel—SYPRO Ruby. Exposure time was 10–15 s.

For 3D reconstruction, exposure times during imaging varied from 1/160 to 1/640 s. The size of the images was 2464 × 1632 pixels (8 bits/pixel). The panoramic image of each section was produced using PTGui 9.1.8 Pro at 300 × 300 resolution. Alignment of panoramic image series was carried out using IGL Align sEM Align (version 1.20b). Muscle fibre 3D reconstructions were created using the IGL Trace software (version 1.20b), courtesy of Dr John Fiala (Boston University, USA, http//www.synapses.bu.edu/). Muscle fibre volumes were measured using Actify’s 3D View according to the following parameters: Model–Mass properties–Volume (render mode, smooth shading).

## Supplementary information

Supplementary file 1

## Data Availability

All data generated or analysed in the course of this research (including files of additional information) were incorporated into the article. Initial gels and Western blots can be accessed at: https://drive.google.com/open?id=1XljyCN3sfWktjuHRPACab3eQfOc6i2AC. 3D reconstruction data are at: https://drive.google.com/drive/folders/1esCaqzZivXVBWh33c3kZNzuJj1f3F3w3?usp=sharing; https://drive.google.com/drive/folders/1ZSFpcdn5U-v3J0lTguC8qXt8Q7jBzshx?usp=sharing; https://drive.google.com/drive/folders/1-XJ5GrqSEaWTTnQVNl4Zy_e8-pnJi2dc?usp=sharing. Final magnifications for electron microscopy images were calculated according to the images of cross grating replicas (2,160 lines/mm; https://www.2spi.com/item/02902-ab/) imaged at the same magnification. Unique searchable RRID identifiers provided by the Resource Identification Portal are as follows: antibodies against calpain-1, RRID: AB_725819; calpastatin, RRID:AB_725881; GAPDH, RRID:AB_732652; goat anti-rabbit Ig, RRID:AB_954595; goat anti-mouse Ig, RRID:AB_954670.
