# Supplementary material for: Predominant synthesis of giant myofibrillar proteins in striated muscles of the long-tailed ground squirrel Urocitellus undulatus during interbout arousal
Source: Sci Rep. 2020 Sep 16;10:15185. doi: 10.1038/s41598-020-72127-y (PMC7495002; doi:10.1038/s41598-020-72127-y)
Supplement: Supplementary file 1 [file 41598_2020_72127_MOESM1_ESM.docx]

**Predominant synthesis of giant myofibrillar proteins in striated muscles of the long-tailed ground squirrel *Urocitellus undulatus* during interbout arousal** *Svetlana Popova1, Anna Ulanova1, Yulia Gritsyna1, Nikolay Salmov1, Vadim Rogachevsky2, Gulnara Mikhailova1, Alexander Bobylev1, Liya Bobyleva1, Yana Yutskevich3, Oleg Morenkov4, Nadezda Zakharova5 & Ivan Vikhlyantsev1,**

1Laboratory of the Structure and Functions of Muscle Proteins, Institute of Theoretical and Experimental Biophysics, Russian Academy of Sciences, Pushchino, Moscow Region, 142290, Russia; 2Laboratory of Signal Perception Mechanisms, Institute of Cell Biophysics, FRC PSCBR, Russian Academy of Sciences, Pushchino, Moscow Region, 142290, Russia; 3Kuban State University, Krasnodar, Krasnodar Krai, 350040, Russia; 4Laboratory of Cell Culture and Tissue Engineering, Institute of Cell Biophysics, FRC PSCBR, Russian Academy of Sciences, Pushchino, Moscow Region, 142290, Russia; 5Laboratory of Natural and Artificial Hypobiosis Mechanisms, Institute of Cell Biophysics, FRC PSCBR, Russian Academy of Sciences, Pushchino, Moscow Region, 142290, Russia

**Supplementary Materials and Figures**

The statistical analysis of the results obtained was carried out with SigmaPlot 11.0 software (Systat Software, Inc., 2008). Since the distribution of some data samples was not normal (Shapiro–Wilk test), we estimated the significance of differences using nonparametric single-factor dispersion analysis for repeated measurements (Kruskal–Wallis One Way Analysis of Variance on Ranks) with the following pairwise comparison by the Tukey’s test. The differences were considered to be statistically significant at *р* < 0.05.

**Supplementary Table S1. Animal body weight, g.**

| **Animals** | **Summer activity (SA), *n*=7** | **Hibernation**  **(HIB), *n*=7** | **Interbout arousal (IBA), *n*=7** |
| --- | --- | --- | --- |
| **1.**  **2.**  **3.**  **4.**  **5.**  **6.**  **7.** | 550  610  660  672  700  715  760 | 410  504  510  519  532  597  630 | 526  566  568  570  598  629  664 |
| Mean±SD | | **666.7±** | | --- | | **69.6** | | | **528.9±** | | --- | | **70.8**** | | | **588.7±** | | --- | | **45.8** | |

**Significant difference vs SA, *p* < 0.01.

**Kruskal-Wallis One Way Analysis of Variance on Ranks**

**Normality Test (Shapiro–Wilk):**  Passed (*P* = 0.506)

**Equal Variance Test (Brown–Forsythe):** Passed (*P* = 0.665)

**Group N Missing Median 25% 75%**

SA 7 0 672.000 610.000 715.000

HIB 7 0 519.000 504.000 597.000

IBA 7 0 570.000 566.000 629.000

*H* = 9.640 with 2 degrees of freedom (*P* = 0.008)

The differences in the median values among the treatment groups are greater than would be expected by chance; **there is a statistically significant difference (*P* = 0.008)**

To isolate the group or groups that differ from the others, use a multiple comparison procedure.

All Pairwise Multiple Comparison Procedures (Tukey’s Test):

**Comparison Diff of Ranks *q* *P*  *P*<0.050**

**SA vs HIB** 72.000 4.386 0.005 **Yes**

SA vs IBA 39.000 2.376 0.213 No

IBA vs HIB 33.000 2.010 0.330 No

Note: The multiple comparisons on ranks do not include an adjustment for ties.

**Predominant synthesis of giant myofibrillar proteins in striated muscles of the long-tailed ground squirrel *Urocitellus undulatus* during interbout arousal** *Svetlana Popova1, Anna Ulanova1, Yulia Gritsyna1, Nikolay Salmov1, Vadim Rogachevsky2, Gulnara Mikhailova1, Alexander Bobylev1, Liya Bobyleva1, Yana Yutskevich3, Oleg Morenkov4, Nadezda Zakharova5 & Ivan Vikhlyantsev1,**

1Laboratory of the Structure and Functions of Muscle Proteins, Institute of Theoretical and Experimental Biophysics, Russian Academy of Sciences, Pushchino, Moscow Region, 142290, Russia; 2Laboratory of Signal Perception Mechanisms, Institute of Cell Biophysics, FRC PSCBR, Russian Academy of Sciences, Pushchino, Moscow Region, 142290, Russia; 3Kuban State University, Krasnodar, Krasnodar Krai, 350040, Russia; 4Laboratory of Cell Culture and Tissue Engineering, Institute of Cell Biophysics, FRC PSCBR, Russian Academy of Sciences, Pushchino, Moscow Region, 142290, Russia; 5Laboratory of Natural and Artificial Hypobiosis Mechanisms, Institute of Cell Biophysics, FRC PSCBR, Russian Academy of Sciences, Pushchino, Moscow Region, 142290, Russia

**Supplementary Table S2. Heart weight, g.**

| **Animals** | **Summer activity (SA), *n*=7** | **Hibernation**  **(HIB), *n*=7** | **Interbout arousal (IBA), *n*=7** |
| --- | --- | --- | --- |
| **1.**  **2.**  **3.**  **4.**  **5.**  **6.**  **7.** | 3.10  4.80  3.35  2.50  3.00  4.50  4.00 | 2.25  3.50  2.65  2.50  3.10  3.40  3.30 | 3.70  2.90  3.85  3.50  3.00  3.00  4.00 |
| Mean±SD | | **3.6±** | | --- | | **0.85** | | | **2.96±** | | --- | | **0.49** | | | **3.42±** | | --- | | **0.45** | |

**Kruskal–Wallis One Way Analysis of Variance on Ranks**

**Normality Test (Shapiro–Wilk):**  Passed (*P* = 0.596)

**Equal Variance Test (Brown–Forsythe):** Passed (*P* = 0.139)

**Group N Missing Median 25% 75%**

SA 7 0 3.350 3.000 4.500

HIB 7 0 3.100 2.500 3.400

IBA 7 0 3.500 3.000 3.850

*H* = 2.643 with 2 degrees of freedom (*P* = 0.267)

The differences in the median values among the treatment groups are not great enough to exclude the possibility that the difference is due to random sampling variability; **there is no statistically significant difference (*P* = 0.267)**

**Predominant synthesis of giant myofibrillar proteins in striated muscles of the long-tailed ground squirrel *Urocitellus undulatus* during interbout arousal** *Svetlana Popova1, Anna Ulanova1, Yulia Gritsyna1, Nikolay Salmov1, Vadim Rogachevsky2, Gulnara Mikhailova1, Alexander Bobylev1, Liya Bobyleva1, Yana Yutskevich3, Oleg Morenkov4, Nadezda Zakharova5 & Ivan Vikhlyantsev1,**

1Laboratory of the Structure and Functions of Muscle Proteins, Institute of Theoretical and Experimental Biophysics, Russian Academy of Sciences, Pushchino, Moscow Region, 142290, Russia; 2Laboratory of Signal Perception Mechanisms, Institute of Cell Biophysics, FRC PSCBR, Russian Academy of Sciences, Pushchino, Moscow Region, 142290, Russia; 3Kuban State University, Krasnodar, Krasnodar Krai, 350040, Russia; 4Laboratory of Cell Culture and Tissue Engineering, Institute of Cell Biophysics, FRC PSCBR, Russian Academy of Sciences, Pushchino, Moscow Region, 142290, Russia; 5Laboratory of Natural and Artificial Hypobiosis Mechanisms, Institute of Cell Biophysics, FRC PSCBR, Russian Academy of Sciences, Pushchino, Moscow Region, 142290, Russia

**Supplementary Table S3. Heart weight/animal body weight, mg/g.**

| **Animals** | **Summer activity (SA), *n*=7** | **Hibernation**  **(HIB), *n*=7** | **Interbout arousal (IBA), *n*=7** |
| --- | --- | --- | --- |
| **1.**  **2.**  **3.**  **4.**  **5.**  **6.**  **7.** | 5.64  7.87  5.08  3.72  4.29  6.29  5.26 | 5.49  6.94  5.20  4.82  5.83  5.70  5.24 | 7.03  5.12  6.78  6.14  5.02  4.77  6.02 |
| Mean±SD | | **5.45±** | | --- | | **1.36** | | | **5.6±** | | --- | | **0.68** | | | **5.8±** | | --- | | **0.89** | |

**Kruskal–Wallis One Way Analysis of Variance on Ranks**

**Normality Test (Shapiro–Wilk):**  Passed (*P* = 0.743)

**Equal Variance Test (Brown–Forsythe):** Passed (*P* = 0.293)

**Group N Missing Median 25% 75%**

SA 7 0 5.260 4.290 6.290

HIB 7 0 5.490 5.200 5.830

IBA 7 0 6.020 5.020 6.780

*H* = 0.423 with 2 degrees of freedom (*P* = 0.809)

The differences in the median values among the treatment groups are not great enough to exclude the possibility that the difference is due to random sampling variability; **there is no statistically significant difference (*P* = 0.809)**

**Predominant synthesis of giant myofibrillar proteins in striated muscles of the long-tailed ground squirrel *Urocitellus undulatus* during interbout arousal** *Svetlana Popova1, Anna Ulanova1, Yulia Gritsyna1, Nikolay Salmov1, Vadim Rogachevsky2, Gulnara Mikhailova1, Alexander Bobylev1, Liya Bobyleva1, Yana Yutskevich3, Oleg Morenkov4, Nadezda Zakharova5 & Ivan Vikhlyantsev1,**

1Laboratory of the Structure and Functions of Muscle Proteins, Institute of Theoretical and Experimental Biophysics, Russian Academy of Sciences, Pushchino, Moscow Region, 142290, Russia; 2Laboratory of Signal Perception Mechanisms, Institute of Cell Biophysics, FRC PSCBR, Russian Academy of Sciences, Pushchino, Moscow Region, 142290, Russia; 3Kuban State University, Krasnodar, Krasnodar Krai, 350040, Russia; 4Laboratory of Cell Culture and Tissue Engineering, Institute of Cell Biophysics, FRC PSCBR, Russian Academy of Sciences, Pushchino, Moscow Region, 142290, Russia; 5Laboratory of Natural and Artificial Hypobiosis Mechanisms, Institute of Cell Biophysics, FRC PSCBR, Russian Academy of Sciences, Pushchino, Moscow Region, 142290, Russia

**Supplementary Table S4, which summarizes Tables S1–S3.**

| **Groups** | **Animals** | **Animal body weight, g** | **Heart weight, g** | **Heart weight/animal body weight, mg/g** |
| --- | --- | --- | --- | --- |
| **Summer activity (SA)** | **1.**  **2.**  **3.**  **4.**  **5.**  **6.**  **7.** | 550  610  660  672  700  715  760  (666.7±69.6) | 3.10  4.80  3.35  2.50  3.00  4.50  4.00  (3.6±0.85) | 5.64  7.87  5.08  3.72  4.29  6.29  5.26  (5.45±1.36) |
|  |  |  |  |  |
| **Hibernation**  **(HIB)** | **1.**  **2.**  **3.**  **4.**  **5.**  **6.**  **7.** | 410  504  510  519  532  597  630  (528.9±70.8**) | 2.25  3.50  2.65  2.50  3.10  3.40  3.30  (2.96±0.49) | 5.49  6.94  5.20  4.82  5.83  5.70  5.24  (5.6±0.68) |
|  |  |  |  |  |
| **Interbout arousal (IBA)** | **1.**  **2.**  **3.**  **4.**  **5.**  **6.**  **7.** | 526  566  568  570  598  629  664  (588.7±45.8) | 3.70  2.90  3.85  3.50  3.00  3.00  4.00  (3.42±0.45) | 7.03  5.12  6.78  6.14  5.02  4.77  6.02  (5.8±0.89) |

**Significant difference vs SA, *p* < 0.01.

Long-tailed ground squirrels (*Urocitellus undulatus*) of both sexes (body mass, 450–750 g, estimated to be between 1 and 2 years of age) were captured in early August 2017 and early August 2018 in Yakutiya (Siberia), transported by air to Pushchino (Moscow Region) and housed in individual cages (74 × 57 × 55 cm) in a specially equipped vivarium under natural photoperiodicity. Food was supplemented with sunflower seeds and carrots, and nesting material was provided *ad libitum*. Early in November, the animals were weighed (Supplementary Table S5), and then the cages with the animals were transferred to a darkroom with a temperature of 1–3°C for the onset of the hibernation season. Part of the animals was individually placed in wooden hibernation boxes (20 × 20 × 25 cm). Experiments were carried out with three groups of animals taken at different phases of their annual cycle: 1) hibernation (HIB; hypothermia, January–February 2018, *n* = 3; the last 10 days of December 2018, *n* = 1; January–February 2019, *n* = 2; the first 10 days of March 2019, *n* = 1); (2) interbout arousal (IBA; normothermia, winter activity, during the first 5–12 h after the spontaneous arousal, January–February 2018, *n* = 3; January–February 2019, *n*= 4); (3) summer activity (SA; normothermia, June–July 2018, *n* = 4; June–July 2019, *n* = 3).

**Predominant synthesis of giant myofibrillar proteins in striated muscles of the long-tailed ground squirrel *Urocitellus undulatus* during interbout arousal** *Svetlana Popova1, Anna Ulanova1, Yulia Gritsyna1, Nikolay Salmov1, Vadim Rogachevsky2, Gulnara Mikhailova1, Alexander Bobylev1, Liya Bobyleva1, Yana Yutskevich3, Oleg Morenkov4, Nadezda Zakharova5 & Ivan Vikhlyantsev1,**

1Laboratory of the Structure and Functions of Muscle Proteins, Institute of Theoretical and Experimental Biophysics, Russian Academy of Sciences, Pushchino, Moscow Region, 142290, Russia; 2Laboratory of Signal Perception Mechanisms, Institute of Cell Biophysics, FRC PSCBR, Russian Academy of Sciences, Pushchino, Moscow Region, 142290, Russia; 3Kuban State University, Krasnodar, Krasnodar Krai, 350040, Russia; 4Laboratory of Cell Culture and Tissue Engineering, Institute of Cell Biophysics, FRC PSCBR, Russian Academy of Sciences, Pushchino, Moscow Region, 142290, Russia; 5Laboratory of Natural and Artificial Hypobiosis Mechanisms, Institute of Cell Biophysics, FRC PSCBR, Russian Academy of Sciences, Pushchino, Moscow Region, 142290, Russia

**Supplementary Table S5. Weight of animals before the start of the hibernation season, after torpor and during interbout arousal.**

| **Groups** | **Animals** | **Animal body weight (g)**  **(early November 2017/2018, before the start of the hibernation season)** | **Animal body weight, g**  **(after torpor or during interbout arousal)** |
| --- | --- | --- | --- |
| **Hibernation**  **(HIB)** | **1.**  **2.**  **3.**  **4.**  **5.**  **6.**  **7.** | 535  632  645  635  702  778  845  (681.7±95,5) | 410  504  510  519  532  597  630  (528.9±70.8)**  **↓22.4%** |
|  |  |  |  |
| **Interbout arousal (IBA)** | **1.**  **2.**  **3.**  **4.**  **5.**  **6.**  **7.** | 595  646  679  684  698  765  836  (700.4±73.0) | 526  566  568  570  598  629  664  (588.7±45.8)**  **↓15.9%** |

The data were analyzed using the nonparametric Mann–Whitney U criterion.

*****p*<0.01.**

**Predominant synthesis of giant myofibrillar proteins in striated muscles of the long-tailed ground squirrel *Urocitellus undulatus* during interbout arousal** *Svetlana Popova1, Anna Ulanova1, Yulia Gritsyna1, Nikolay Salmov1, Vadim Rogachevsky2, Gulnara Mikhailova1, Alexander Bobylev1, Liya Bobyleva1, Yana Yutskevich3, Oleg Morenkov4, Nadezda Zakharova5 & Ivan Vikhlyantsev1,**

1Laboratory of the Structure and Functions of Muscle Proteins, Institute of Theoretical and Experimental Biophysics, Russian Academy of Sciences, Pushchino, Moscow Region, 142290, Russia; 2Laboratory of Signal Perception Mechanisms, Institute of Cell Biophysics, FRC PSCBR, Russian Academy of Sciences, Pushchino, Moscow Region, 142290, Russia; 3Kuban State University, Krasnodar, Krasnodar Krai, 350040, Russia; 4Laboratory of Cell Culture and Tissue Engineering, Institute of Cell Biophysics, FRC PSCBR, Russian Academy of Sciences, Pushchino, Moscow Region, 142290, Russia; 5Laboratory of Natural and Artificial Hypobiosis Mechanisms, Institute of Cell Biophysics, FRC PSCBR, Russian Academy of Sciences, Pushchino, Moscow Region, 142290, Russia

**Supplementary Table S6. GAPDH levels in the heart of ground squirrels, %.**

| **Summer activity (SA), *n*=6** | **Hibernation (HIB),  *n*=6** | **Interbout arousal (IBA), *n*=6** |
| --- | --- | --- |
| 100.0 | 91.0 | 88.2 |
| 100.0 | 92.0 | 93.6 |
| 100.0 | 99.3 | 99.6 |
| 100.0 | 100.7 | 107.6 |
| 100.0 | 104.8 | 118.0 |
| 100.0 | 107.2 | 118.9 |
| **100,0±5.0%** | **99.2±6.6%** | **104.3±12.7%** |

GAPDH content of the SA group was taken as 100% (control group). For each individual experiment, the average content of 2–3 GAPDH spots (from the control SA group) present on the blot membrane was taken into consideration.

**Kruskal–Wallis One Way Analysis of Variance on Ranks**

**Normality Test (Shapiro–Wilk):**  Passed (*P* = 0.368)

**Equal Variance Test (Brown–Forsythe):** Failed (*P* < 0.050)

**Group N Missing Median 25% 75%**

SA 6 0 100.000 100.000 100.000

HIB 6 0 100.000 91.750 105.400

IBA 6 0 103.600 92.250 118.225

*H* = 0.303 with 2 degrees of freedom (*P* = 0.859)

The differences in the median values among the treatment groups are not great enough to exclude the possibility that the difference is due to random sampling variability; **there is no statistically significant difference (*P* = 0.859)**

**Predominant synthesis of giant myofibrillar proteins in striated muscles of the long-tailed ground squirrel *Urocitellus undulatus* during interbout arousal** *Svetlana Popova1, Anna Ulanova1, Yulia Gritsyna1, Nikolay Salmov1, Vadim Rogachevsky2, Gulnara Mikhailova1, Alexander Bobylev1, Liya Bobyleva1, Yana Yutskevich3, Oleg Morenkov4, Nadezda Zakharova5 & Ivan Vikhlyantsev1,**

1Laboratory of the Structure and Functions of Muscle Proteins, Institute of Theoretical and Experimental Biophysics, Russian Academy of Sciences, Pushchino, Moscow Region, 142290, Russia; 2Laboratory of Signal Perception Mechanisms, Institute of Cell Biophysics, FRC PSCBR, Russian Academy of Sciences, Pushchino, Moscow Region, 142290, Russia; 3Kuban State University, Krasnodar, Krasnodar Krai, 350040, Russia; 4Laboratory of Cell Culture and Tissue Engineering, Institute of Cell Biophysics, FRC PSCBR, Russian Academy of Sciences, Pushchino, Moscow Region, 142290, Russia; 5Laboratory of Natural and Artificial Hypobiosis Mechanisms, Institute of Cell Biophysics, FRC PSCBR, Russian Academy of Sciences, Pushchino, Moscow Region, 142290, Russia

**Supplementary Table S7. GAPDH levels in m. longissimus dorsi of ground squirrels, %.**

| **Summer activity (SA), *n*=7** | **Hibernation (HIB), *n*=7** | **Interbout arousal (IBA), *n*=7** |
| --- | --- | --- |
| 100.0 | 93.7 | 96.5 |
| 100.0 | 97.6 | 98.3 |
| 100.0 | 98.5 | 100.9 |
| 100.0 | 99.3 | 101.4 |
| 100.0 | 101.5 | 102.7 |
| 100.0 | 101.7 | 103.0 |
| 100.0 | 102.5 | 103.9 |
| **100.0±3.7%** | **99.3±3.1%** | **100.9±2.7%** |

GAPDH content of the SA group was taken as 100% (control group). For each individual experiment, the average content of 2–3 GAPDH spots (from the control SA group) present on the blot membrane was taken into consideration.

**Kruskal–Wallis One Way Analysis of Variance on Ranks**

**Normality Test (Shapiro–Wilk):**  Failed (*P* < 0.050)

**Group N Missing Median 25% 75%**

SA 7 0 100.000 100.000 100.000

HIB 7 0 99.300 97.600 101.700

IBA 7 0 101.400 98.300 103.000

*H* = 1.902 with 2 degrees of freedom (*P* = 0.386)

The differences in the median values among the treatment groups are not great enough to exclude the possibility that the difference is due to random sampling variability; **there is no statistically significant difference (*P* = 0.386)**

**Predominant synthesis of giant myofibrillar proteins in striated muscles of the long-tailed ground squirrel *Urocitellus undulatus* during interbout arousal** *Svetlana Popova1, Anna Ulanova1, Yulia Gritsyna1, Nikolay Salmov1, Vadim Rogachevsky2, Gulnara Mikhailova1, Alexander Bobylev1, Liya Bobyleva1, Yana Yutskevich3, Oleg Morenkov4, Nadezda Zakharova5 & Ivan Vikhlyantsev1,**

1Laboratory of the Structure and Functions of Muscle Proteins, Institute of Theoretical and Experimental Biophysics, Russian Academy of Sciences, Pushchino, Moscow Region, 142290, Russia; 2Laboratory of Signal Perception Mechanisms, Institute of Cell Biophysics, FRC PSCBR, Russian Academy of Sciences, Pushchino, Moscow Region, 142290, Russia; 3Kuban State University, Krasnodar, Krasnodar Krai, 350040, Russia; 4Laboratory of Cell Culture and Tissue Engineering, Institute of Cell Biophysics, FRC PSCBR, Russian Academy of Sciences, Pushchino, Moscow Region, 142290, Russia; 5Laboratory of Natural and Artificial Hypobiosis Mechanisms, Institute of Cell Biophysics, FRC PSCBR, Russian Academy of Sciences, Pushchino, Moscow Region, 142290, Russia

**Supplementary Table S8. Muscle fibre volumes of m. longissimus dorsi, × 1000 µm3.**

| **Fragments of m. longissimus dorsi muscle fibres** | **Summer activity (SA), *n*=5** | **Hibernation (HIB), *n*=5** | **Interbout arousal (IBA), *n*=4** |
| --- | --- | --- | --- |
|  | **Animal No. 1** | **Animal No. 1** | **Animal No. 1** |
|  | 785 | 253 | 404 |
|  | 851 | 249 | 493 |
|  | 624 | 274 | 287 |
|  | 755 | 324 | 382 |
|  | 672 | 302 | 326 |
|  | 569 | 227 | 407 |
|  | 619 | 343 | 363 |
|  | 625 | 241 | 267 |
|  | 502 | 241 | 620 |
|  | 686 | 357 | 426 |
|  | 559 | 324 | 516 |
|  | 643 | 286 | 536 |
|  | 710 | 243 | 558 |
|  | 632 | 250 | 430 |
|  | 747 | 260 | 424 |
|  | 830 | 258 | 342 |
|  | 742 | 247 | 431 |
|  | 546 | 246 | 461 |
|  | 934 | 293 | 477 |
|  | 717 | 189 | 584 |
|  | 571 | 260 | 634 |
|  | 535 | 244 | 509 |
|  | 676 | 271 | 399 |
|  | 758 | 250 | 535 |
|  | 702 | 300 | 371 |
|  | 591 | 264 | 396 |
|  | 555 | 277 | 463 |
|  | 702 | 262 | 435 |
|  | 443 | 300 | 338 |
|  | 465 | 218 | 411 |
|  | 784 | 219 | 364 |
|  | 335 | 278 | 361 |
|  | 668 | 287 | 400 |
|  | 740 | 406 | 348 |
|  | 662 | 344 | 373 |
|  | 582 | 263 | 321 |
|  | 682 | 223 | 434 |
|  | 421 | 271 | 334 |
|  | 524 | 275 | 396 |
|  | 682 | 243 | 400 |
|  | 590 | 237 | 318 |
|  | 534 | 258 | 303 |
|  | 600 | 260 | 310 |
|  | 634 | 208 | 367 |
|  | 676 | 263 | 367 |
|  | 682 | 282 | 400 |
|  | 628 | 281 | 343 |
|  | 554 | 301 | 435 |
|  | 300 | 300 | 450 |
|  | 376 | 260 | 354 |
|  | 540 | 408 | 301 |
|  | 511 | 232 | 260 |
|  | 640 | 220 | 415 |
|  | 605 | 220 | 311 |
|  | 650 | 215 | 507 |
|  | 693 | 231 | 557 |
|  | 607 | 269 | 353 |
|  | 399 | 264 | 271 |
|  | 611 | 259 | 285 |
|  | 741 | 434 | 388 |
|  | 543 | 360 | 464 |
|  | 507 | 308 | 487 |
|  | 748 | 524 | 453 |
|  | 492 | 316 | 464 |
|  | 564 | 322 | 484 |
|  | 607 | 286 | 575 |
|  | 568 | 256 | 490 |
|  | 602 | 278 | 509 |
|  | 765 | 296 | 399 |
|  | 505 | 347 | 474 |
|  | 610 | 247 | 446 |
|  | 606 | 232 | 373 |
|  | 720 | 256 | 323 |
|  | 600 | 272 | 309 |
|  | 465 | 334 | 600 |
|  |  |  |  |
|  | **Animal No. 2** | **Animal No. 2** | **Animal No. 2** |
|  | 304 | 711 | 401 |
|  | 467 | 487 | 347 |
|  | 584 | 579 | 451 |
|  | 562 | 632 | 390 |
|  | 581 | 669 | 448 |
|  | 455 | 613 | 411 |
|  | 529 | 615 | 419 |
|  | 405 | 545 | 420 |
|  | 309 | 662 | 380 |
|  | 359 | 529 | 444 |
|  | 285 | 465 | 544 |
|  | 333 | 533 | 475 |
|  | 508 | 445 | 474 |
|  | 500 | 541 | 498 |
|  | 418 | 540 | 510 |
|  | 239 | 537 | 511 |
|  | 373 | 461 | 426 |
|  | 384 | 605 | 257 |
|  | 481 | 585 | 474 |
|  | 336 | 300 | 470 |
|  | 651 | 516 | 500 |
|  | 317 | 532 | 312 |
|  | 340 | 301 | 391 |
|  | 380 | 527 | 419 |
|  | 280 | 606 | 405 |
|  | 535 | 686 | 397 |
|  | 300 | 567 | 376 |
|  | 241 | 576 | 508 |
|  | 390 | 601 | 301 |
|  | 507 | 519 | 508 |
|  | 281 | 587 | 375 |
|  | 270 | 564 | 330 |
|  | 558 | 536 | 324 |
|  | 506 | 481 | 531 |
|  | 313 | 597 | 449 |
|  | 308 | 452 | 296 |
|  | 330 | 476 | 322 |
|  | 425 | 484 | 294 |
|  | 361 | 474 | 286 |
|  | 364 | 630 | 267 |
|  | 533 | 456 | 448 |
|  | 871 | 309 | 364 |
|  | 389 | 449 | 321 |
|  | 584 | 379 | 348 |
|  | 713 | 523 | 389 |
|  | 562 | 434 | 309 |
|  | 478 | 486 | 310 |
|  | 554 | 300 | 285 |
|  | 451 | 451 | 283 |
|  | 680 | 554 | 290 |
|  | 571 | 498 | 336 |
|  | 703 | 375 | 307 |
|  | 598 | 502 | 280 |
|  | 605 | 628 | 299 |
|  | 455 | 543 | 319 |
|  | 542 | 630 | 362 |
|  | 504 | 491 | 381 |
|  | 822 | 384 | 322 |
|  | 471 | 431 | 353 |
|  | 351 | 567 | 340 |
|  | 648 | 511 | 308 |
|  | 377 | 440 | 234 |
|  | 577 | 563 | 298 |
|  | 530 | 387 | 276 |
|  | 612 | 322 | 370 |
|  | 642 | 494 | 317 |
|  | 784 | 603 | 355 |
|  | 653 | 630 | 286 |
|  | 486 | 379 | 320 |
|  | 658 | 440 | 326 |
|  | 624 | 458 | 363 |
|  | 673 | 496 | 274 |
|  | 533 | 355 | 336 |
|  | 495 | 623 | 335 |
|  | 627 | 395 | 349 |
|  |  |  |  |
|  | **Animal No. 3** | **Animal No. 3** | **Animal No. 3** |
|  | 312 | 448 | 361 |
|  | 431 | 298 | 340 |
|  | 541 | 395 | 261 |
|  | 580 | 284 | 276 |
|  | 541 | 306 | 309 |
|  | 384 | 330 | 300 |
|  | 344 | 400 | 301 |
|  | 435 | 347 | 317 |
|  | 443 | 309 | 269 |
|  | 568 | 334 | 282 |
|  | 479 | 440 | 328 |
|  | 402 | 319 | 307 |
|  | 353 | 329 | 544 |
|  | 636 | 337 | 438 |
|  | 436 | 362 | 418 |
|  | 334 | 325 | 460 |
|  | 605 | 375 | 354 |
|  | 480 | 301 | 361 |
|  | 604 | 316 | 259 |
|  | 383 | 327 | 240 |
|  | 405 | 374 | 458 |
|  | 380 | 362 | 304 |
|  | 377 | 371 | 248 |
|  | 382 | 344 | 338 |
|  | 318 | 473 | 255 |
|  | 370 | 317 | 243 |
|  | 771 | 304 | 314 |
|  | 623 | 363 | 265 |
|  | 340 | 337 | 366 |
|  | 423 | 396 | 316 |
|  | 439 | 372 | 336 |
|  | 397 | 360 | 288 |
|  | 444 | 263 | 244 |
|  | 418 | 385 | 311 |
|  | 394 | 317 | 276 |
|  | 354 | 348 | 226 |
|  | 346 | 388 | 268 |
|  | 281 | 396 | 317 |
|  | 337 | 330 | 373 |
|  | 407 | 292 | 321 |
|  | 450 | 319 | 370 |
|  | 618 | 300 | 227 |
|  | 470 | 283 | 246 |
|  | 588 | 292 | 353 |
|  | 471 | 231 | 299 |
|  | 446 | 298 | 215 |
|  | 561 | 217 | 330 |
|  | 603 | 279 | 338 |
|  | 560 | 269 | 368 |
|  | 714 | 264 | 444 |
|  | 573 | 318 | 280 |
|  | 619 | 293 | 356 |
|  | 362 | 368 | 480 |
|  | 441 | 281 | 360 |
|  | 780 | 269 | 410 |
|  | 688 | 293 | 471 |
|  | 486 | 273 | 196 |
|  | 555 | 312 | 188 |
|  | 401 | 302 | 265 |
|  | 301 | 297 | 273 |
|  | 421 | 344 | 281 |
|  | 391 | 309 | 413 |
|  | 352 | 398 | 418 |
|  | 290 | 259 | 265 |
|  | 415 | 273 | 464 |
|  | 328 | 283 | 301 |
|  | 370 | 301 | 446 |
|  | 495 | 317 | 413 |
|  | 595 | 370 | 365 |
|  | 328 | 408 | 347 |
|  | 528 | 240 | 392 |
|  | 449 | 281 | 307 |
|  | 614 | 309 | 347 |
|  | 544 | 297 | 414 |
|  | 428 | 281 | 476 |
|  |  |  |  |
|  | **Animal No. 4** | **Animal No. 4** | **Animal No. 4** |
|  | 573 | 252 | 445 |
|  | 587 | 279 | 299 |
|  | 566 | 270 | 291 |
|  | 605 | 228 | 441 |
|  | 616 | 295 | 151 |
|  | 444 | 271 | 413 |
|  | 511 | 223 | 254 |
|  | 400 | 266 | 284 |
|  | 467 | 229 | 409 |
|  | 467 | 284 | 376 |
|  | 625 | 279 | 471 |
|  | 468 | 236 | 332 |
|  | 782 | 283 | 379 |
|  | 616 | 287 | 263 |
|  | 514 | 277 | 288 |
|  | 555 | 271 | 451 |
|  | 613 | 246 | 427 |
|  | 645 | 241 | 540 |
|  | 561 | 251 | 735 |
|  | 570 | 278 | 718 |
|  | 582 | 266 | 759 |
|  | 792 | 255 | 508 |
|  | 817 | 258 | 470 |
|  | 677 | 228 | 584 |
|  | 718 | 300 | 486 |
|  | 715 | 234 | 505 |
|  | 726 | 244 | 561 |
|  | 783 | 270 | 399 |
|  | 672 | 246 | 411 |
|  | 678 | 232 | 369 |
|  | 609 | 213 | 497 |
|  | 714 | 222 | 518 |
|  | 394 | 266 | 502 |
|  | 661 | 244 | 421 |
|  | 714 | 312 | 440 |
|  | 796 | 266 | 454 |
|  | 716 | 230 | 417 |
|  | 639 | 239 | 433 |
|  | 637 | 272 | 401 |
|  | 761 | 240 | 383 |
|  | 488 | 220 | 380 |
|  | 390 | 228 | 471 |
|  | 499 | 263 | 422 |
|  | 550 | 285 | 395 |
|  | 401 | 246 | 543 |
|  | 535 | 265 | 557 |
|  | 438 | 276 | 568 |
|  | 523 | 239 | 552 |
|  | 435 | 231 | 455 |
|  | 325 | 204 | 488 |
|  | 673 | 286 | 612 |
|  | 437 | 236 | 540 |
|  | 536 | 228 | 653 |
|  | 550 | 196 | 892 |
|  | 370 | 259 | 702 |
|  | 446 | 206 | 671 |
|  | 458 | 216 | 690 |
|  | 541 | 236 | 533 |
|  | 670 | 293 | 500 |
|  | 682 | 273 | 560 |
|  | 769 | 212 | 544 |
|  | 686 | 202 | 613 |
|  | 696 | 297 | 587 |
|  | 576 | 244 | 543 |
|  | 557 | 309 | 616 |
|  | 553 | 221 | 633 |
|  | 760 | 259 | 669 |
|  | 594 | 273 | 911 |
|  | 496 | 283 | 424 |
|  | 421 | 301 | 371 |
|  | 570 | 297 | 526 |
|  | 465 | 270 | 658 |
|  | 574 | 344 | 483 |
|  | 549 | 301 | 421 |
|  | 482 | 230 | 400 |
|  |  |  |  |
|  | **Animal No. 5** | **Animal No. 5** |  |
|  | 572 | 454 |  |
|  | 469 | 421 |  |
|  | 529 | 474 |  |
|  | 463 | 485 |  |
|  | 427 | 437 |  |
|  | 500 | 318 |  |
|  | 428 | 544 |  |
|  | 587 | 349 |  |
|  | 554 | 461 |  |
|  | 454 | 491 |  |
|  | 587 | 405 |  |
|  | 531 | 412 |  |
|  | 502 | 496 |  |
|  | 425 | 507 |  |
|  | 400 | 537 |  |
|  | 547 | 406 |  |
|  | 446 | 439 |  |
|  | 586 | 436 |  |
|  | 556 | 493 |  |
|  | 473 | 495 |  |
|  | 606 | 434 |  |
|  | 525 | 566 |  |
|  | 607 | 429 |  |
|  | 495 | 499 |  |
|  | 527 | 497 |  |
|  | 455 | 522 |  |
|  | 465 | 546 |  |
|  | 561 | 445 |  |
|  | 518 | 347 |  |
|  | 532 | 379 |  |
|  | 428 | 366 |  |
|  | 585 | 413 |  |
|  | 522 | 402 |  |
|  | 552 | 533 |  |
|  | 587 | 374 |  |
|  | 540 | 477 |  |
|  | 423 | 358 |  |
|  | 499 | 564 |  |
|  | 500 | 380 |  |
|  | 570 | 390 |  |
|  | 563 | 380 |  |
|  | 404 | 525 |  |
|  | 567 | 398 |  |
|  | 484 | 471 |  |
|  | 562 | 410 |  |
|  | 481 | 339 |  |
|  | 555 | 356 |  |
|  | 529 | 570 |  |
|  | 505 | 380 |  |
|  | 509 | 380 |  |
|  | 559 | 460 |  |
|  | 485 | 398 |  |
|  | 533 | 557 |  |
|  | 508 | 605 |  |
|  | 500 | 524 |  |
|  | 418 | 572 |  |
|  | 439 | 606 |  |
|  | 573 | 580 |  |
|  | 484 | 350 |  |
|  | 481 | 500 |  |
|  | 436 | 422 |  |
|  | 451 | 450 |  |
|  | 517 | 401 |  |
|  | 340 | 436 |  |
|  | 380 | 507 |  |
|  | 480 | 469 |  |
|  | 535 | 490 |  |
|  | 400 | 532 |  |
|  | 341 | 600 |  |
|  | 390 | 560 |  |
|  | 507 | 349 |  |
|  | 381 | 489 |  |
|  | 370 | 400 |  |
|  | 458 | 450 |  |
|  | 506 | 550 |  |
| **Mean ± SEM (SD)** | **528.8±6.7 (SD=129.3)** | **365.9±6.2 (SD=119.9)** | **404.0±6.5 (SD=113.3)** |
|  | **100.0%** | **69.19%**** | **76.4% **##** |

****Significant difference vs SA, *p* < 0.01. ##** **Significant difference vs HIB, *p* < 0.01.**

**Normality Test (Shapiro–Wilk)**

**Data source:** Data 1 in Notebook1

SA: W-Statistic = 0.992 *P* = 0.054 Passed

HIB: W-Statistic = 0.920 *P* < 0.001 Failed

IBA: W-Statistic = 0.948 *P* < 0.001 Failed

A test that fails indicates that the data varies significantly from the pattern expected if the data was drawn from a population with a normal distribution.

A test that passes indicates that the data matches the pattern expected if the data was drawn from a population with a normal distribution.

**Kruskal–Wallis One Way Analysis of Variance on Ranks**

**Data source:** Data 1 in Notebook1

**Group N Missing Median 25% 75%**

SA 375 0 533.000 435.250 608.500

HIB 375 0 325.000 269.250 455.500

IBA 300 0 391.500 319.500 470.000

*H* = 274.269 with 2 degrees of freedom (*P* = <0.001)

The differences in the median values among the treatment groups are greater than would be expected by chance; there is a statistically significant difference (*P* = <0.001)

To isolate the group or groups that differ from the others, use a multiple comparison procedure.

All Pairwise Multiple Comparison Procedures (Dunn's Method):

**Comparison Diff of Ranks *Q* *P*<0.05**

SA vs HIB 355.525 16.053 Yes

SA vs IBA 262.052 11.156 Yes

IBA vs HIB 93.473 3.979 Yes

**Predominant synthesis of giant myofibrillar proteins in striated muscles of the long-tailed ground squirrel *Urocitellus undulatus* during interbout arousal** *Svetlana Popova1, Anna Ulanova1, Yulia Gritsyna1, Nikolay Salmov1, Vadim Rogachevsky2, Gulnara Mikhailova1, Alexander Bobylev1, Liya Bobyleva1, Yana Yutskevich3, Oleg Morenkov4, Nadezda Zakharova5 & Ivan Vikhlyantsev1,**

1Laboratory of the Structure and Functions of Muscle Proteins, Institute of Theoretical and Experimental Biophysics, Russian Academy of Sciences, Pushchino, Moscow Region, 142290, Russia; 2Laboratory of Signal Perception Mechanisms, Institute of Cell Biophysics, FRC PSCBR, Russian Academy of Sciences, Pushchino, Moscow Region, 142290, Russia; 3Kuban State University, Krasnodar, Krasnodar Krai, 350040, Russia; 4Laboratory of Cell Culture and Tissue Engineering, Institute of Cell Biophysics, FRC PSCBR, Russian Academy of Sciences, Pushchino, Moscow Region, 142290, Russia; 5Laboratory of Natural and Artificial Hypobiosis Mechanisms, Institute of Cell Biophysics, FRC PSCBR, Russian Academy of Sciences, Pushchino, Moscow Region, 142290, Russia


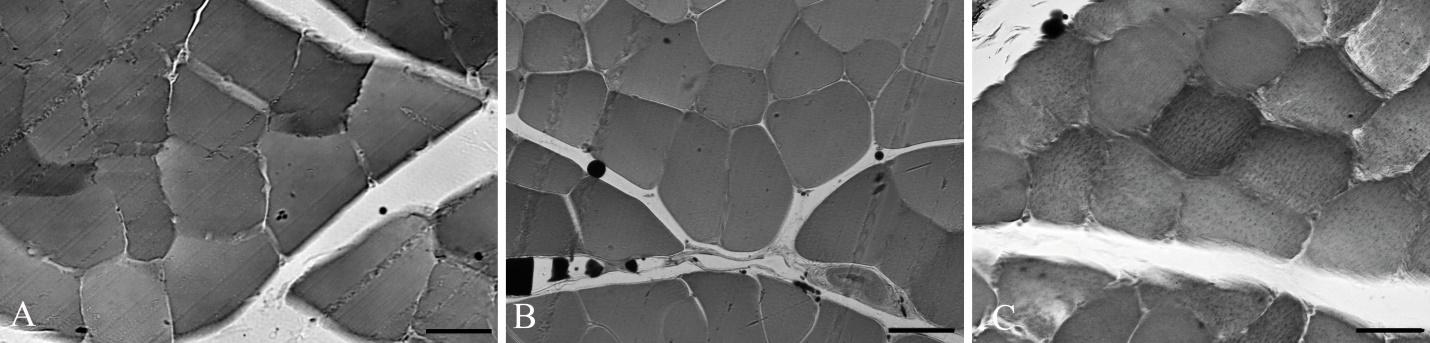


**Supplementary Fig. S1. (**A–C) Micrographs of frontal sections of the m. longissimus dorsi of long-tailed ground squirrels. Glutaraldehyde and osmium fixation, embedded in Epon. (A) Muscle from an SA animal; (B) muscle from an HIB animal; (C) muscle from an IBA animal. Scale bar, 50 µm.

**
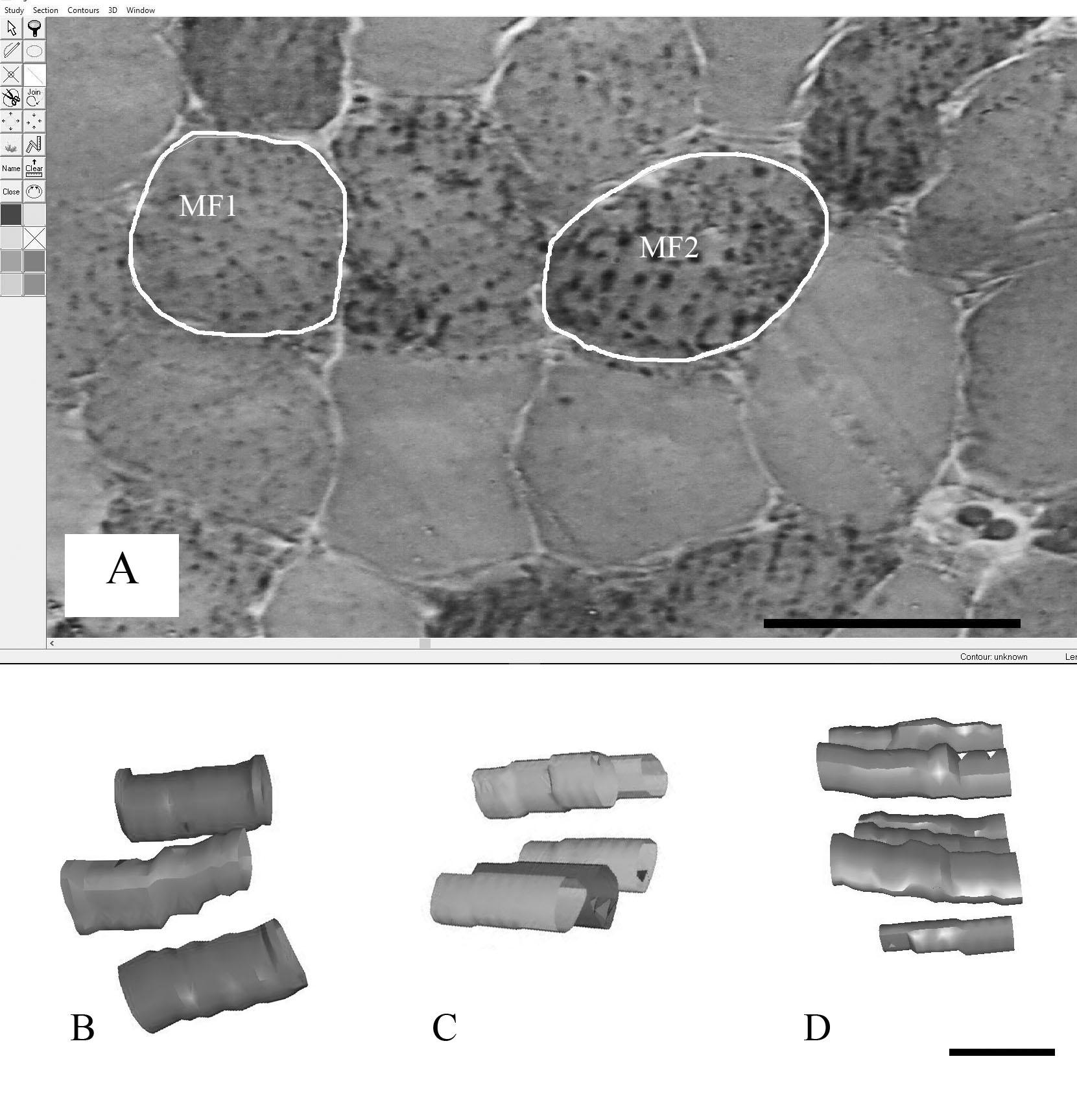
**

**Supplementary Fig. S2.** (**A**) A frontal section of a muscle fragment from an IBA animal after manual retouching in Igl Trace. The white lines around MF1 and MF2 show the contours of two independent muscle fibres. Scale bar, 50 µm. Before the retouching, the coefficient of muscle tissue shrinkage caused by the fixation procedure were calculated. Based on the measurement of the length and width of nine muscle fragments (5 mm in length and 1-mm-thick), prior to the fixation and before embedding the fragments in Epon, the shrinkage coefficients were 4.54±0.46% (by length) and 5.39±093% (by width). (**B–D**) Representative reconstructions of muscle fibres. SA, summer activity; HIB, hibernation; IBA, interbout arousal. (**B**) SA, (**C**) HIB, (**D**) IBA. Scale bar, 100 µm. The method is described in detail in Fiala and Harris (2001). [Fiala, J.C. & Harris, K.M. Extending unbiased stereology of brain ultrastructure to three-dimensional volumes. J. Am. Med. Inform. Assoc. 8(1): 1–16 (2001)].

3D reconstruction data are at:

<https://drive.google.com/drive/folders/1esCaqzZivXVBWh33c3kZNzuJj1f3F3w3?usp=sharing>

<https://drive.google.com/drive/folders/1ZSFpcdn5U-v3J0lTguC8qXt8Q7jBzshx?usp=sharing>

<https://drive.google.com/drive/folders/1-XJ5GrqSEaWTTnQVNl4Zy_e8-pnJi2dc?usp=sharing>

**Predominant synthesis of giant myofibrillar proteins in striated muscles of the long-tailed ground squirrel *Urocitellus undulatus* during interbout arousal** *Svetlana Popova1, Anna Ulanova1, Yulia Gritsyna1, Nikolay Salmov1, Vadim Rogachevsky2, Gulnara Mikhailova1, Alexander Bobylev1, Liya Bobyleva1, Yana Yutskevich3, Oleg Morenkov4, Nadezda Zakharova5 & Ivan Vikhlyantsev1,**

1Laboratory of the Structure and Functions of Muscle Proteins, Institute of Theoretical and Experimental Biophysics, Russian Academy of Sciences, Pushchino, Moscow Region, 142290, Russia; 2Laboratory of Signal Perception Mechanisms, Institute of Cell Biophysics, FRC PSCBR, Russian Academy of Sciences, Pushchino, Moscow Region, 142290, Russia; 3Kuban State University, Krasnodar, Krasnodar Krai, 350040, Russia; 4Laboratory of Cell Culture and Tissue Engineering, Institute of Cell Biophysics, FRC PSCBR, Russian Academy of Sciences, Pushchino, Moscow Region, 142290, Russia; 5Laboratory of Natural and Artificial Hypobiosis Mechanisms, Institute of Cell Biophysics, FRC PSCBR, Russian Academy of Sciences, Pushchino, Moscow Region, 142290, Russia

**Supplementary Table S9. The distribution of fibre volumes between animals in each group
× 1000 µm3.**

|  | **SA** | **HIB** | **IBA** |
| --- | --- | --- | --- |
| **Animal No. 1, Mean ± SEM (SD)** | 617.3±13.5 (SD=116.9) | 278.6±6.3 (SD=54.6) | 414.7±10.2 (SD=88.4) |
| **Animal No. 2, Mean ± SEM (SD)** | 485.7±16.7 (SD=144.2) | 510.0±11.2 (SD=96.7) | 369.8±8.8 (SD=76.4) |
| **Animal No. 3, Mean ± SEM (SD)** | 463.1±13.4 (SD=116.4) | 325.4±5.8 (SD=50.3) | 332.1±8.8 (SD=76.0) |
| **Animal No. 4, Mean ± SEM (SD)** | 582.4±13.7 (SD=118.3) | 256.2±3.5 (SD=30.2) | 499.6±14.9 (SD=128.9) |
| **Animal No. 5, Mean ± SEM (SD)** | 495.5±7.5  (SD=65.2) | 459.3±8.6 (SD=74.2) |  |

**Normality Test (Shapiro–Wilk) (for SA group)**

**Data source:** Data 1 in Notebook1

SA 1: W-Statistic = 0.991 *P* = 0.868 Passed

SA 2: W-Statistic = 0.973 *P* = 0.116 Passed

SA 3: W-Statistic = 0.942 *P* = 0.002 Failed

SA 4: W-Statistic = 0.982 *P* = 0.351 Passed

SA 5: W-Statistic = 0.971 *P* = 0.085 Passed

A test that fails indicates that the data varies significantly from the pattern expected if the data was drawn from a population with a normal distribution.

A test that passes indicates that the data matches the pattern expected if the data was drawn from a population with a normal distribution.

**Kruskal–Wallis One Way Analysis of Variance on Ranks (for SA group)**

**Data source:** Data 1 in Notebook1

**Equal Variance Test:** Failed (*P* < 0.050)

**Group N Missing Median 25% 75%**

SA 1 75 0 611.000 548.000 691.250

SA 2 75 0 495.000 361.750 583.250

SA 3 75 0 436.000 377.750 558.750

SA 4 75 0 573.000 490.000 676.000

SA 5 75 0 502.000 4 51.750 550.750

*H* = 82.501 with 4 degrees of freedom (*P* = <0.001)

The differences in the median values among the treatment groups are greater than would be expected by chance; there is a statistically significant difference (*P* = <0.001)

To isolate the group or groups that differ from the others, use a multiple comparison procedure.

All Pairwise Multiple Comparison Procedures (Tukey’s Test):

**Comparison Diff of Ranks *q* *P*<0.05**

SA 1 vs SA 3 9884.000 10.529 Yes

SA 1 vs SA 2 8169.500 8.703 Yes

SA 1 vs SA 5 7916.000 8.432 Yes

SA 1 vs SA 4 2278.000 2.427 No

SA 4 vs SA 3 7606.000 8.102 Yes

SA 4 vs SA 2 5891.500 6.276 Yes

SA 4 vs SA 5 5638.000 6.006 Yes

SA 5 vs SA 3 1968.000 2.096 No

SA 5 vs SA 2 253.500 0.270 Do Not Test

SA 2 vs SA 3 1714.500 1.826 Do Not Test

Note: The multiple comparisons on ranks do not include an adjustment for ties.

A result of "Do Not Test" occurs for a comparison when no significant difference is found between the two rank sums that enclose that comparison. For example, if you had four rank sums sorted in order, and found no significant difference between rank sums 4 vs 2, then you would not test 4 vs 3 and 3 vs 2, but still test 4 vs 1 and 3 vs 1 (4 vs 3 and 3 vs 2 are enclosed by 4 vs 2: 4 3 2 1). Note that not testing the enclosed rank sums is a procedural rule, and a result of Do Not Test should be treated as if there is no significant difference between the rank sums, even though one may appear to exist.

**Normality Test (Shapiro–Wilk) (for HIB group)**

**Data source:** Data 1 in Notebook1

HIB 1: W-Statistic = 0.859 *P* < 0.001 Failed

HIB 2: W-Statistic = 0.978 *P* = 0.220 Passed

HIB 3: W-Statistic = 0.973 *P* = 0.103 Passed

HIB 4: W-Statistic = 0.983 *P* = 0,413 Passed

HIB 5: W-Statistic = 0.972 *P* = 0.091 Passed

A test that fails indicates that the data varies significantly from the pattern expected if the data was drawn from a population with a normal distribution.

A test that passes indicates that the data matches the pattern expected if the data was drawn from a population with a normal distribution.

**Kruskal–Wallis One Way Analysis of Variance on Ranks (for HIB group)**

**Data source:** Data 1 in Notebook1

**Group N Missing Median 25% 75%**

HIB 1 75 0 264.000 246.250 300.000

HIB 2 75 0 519.000 451.250 583.500

HIB 3 75 0 317.000 292.250 362.000

HIB 4 75 0 258.000 231.250 277.750

HIB 5 75 0 454.000 400.250 518.250

*H* = 272.396 with 4 degrees of freedom (*P* = <0.001)

The differences in the median values among the treatment groups are greater than would be expected by chance; **there is a statistically significant difference (*P* = <0.001)**

To isolate the group or groups that differ from the others, use a multiple comparison procedure.

All Pairwise Multiple Comparison Procedures (Tukey’s Test):

**Comparison Diff of Ranks *q* *P*<0.05**

HIB 2 vs HIB 4 17350.500 18.483 Yes

HIB 2 vs HIB 1 15162.000 16.151 Yes

HIB 2 vs HIB 3 10038.500 10.693 Yes

HIB 2 vs HIB 5 1906.500 2.031 No

HIB 5 vs HIB 4 15444.000 16.452 Yes

HIB 5 vs HIB 1 13255.500 14.120 Yes

HIB 5 vs HIB 3 8132.000 8.663 Yes

HIB 3 vs HIB 4 7312.000 7.789 Yes

HIB 3 vs HIB 1 5123.500 5.458 Yes

HIB 1 vs HIB 4 2188.500 2.331 No

Note: The multiple comparisons on ranks do not include an adjustment for ties.

**Normality Test (Shapiro–Wilk) (for IBA group)**

**Data source:** Data 1 in Notebook1

IBA 1: W-Statistic = 0.975 *P* = 0.145 Passed

IBA 2: W-Statistic = 0.947 *P* = 0,004 Failed

IBA 3: W-Statistic = 0.972 *P* = 0.089 Passed

IBA 4: W-Statistic = 0.949 *P* = 0.004 Failed

A test that fails indicates that the data varies significantly from the pattern expected if the data was drawn from a population with a normal distribution.

A test that passes indicates that the data matches the pattern expected if the data was drawn from a population with a normal distribution.

**Kruskal–Wallis One Way Analysis of Variance on Ranks (for IBA group)**

**Data source:** Data 1 in Notebook1

**Group N Missing Median 25% 75%**

IBA 1 75 0 400.000 349.250 471.500

IBA 2 75 0 353.000 309.250 419.750

IBA 3 75 0 317.000 273.750 369.500

IBA 4 75 0 483.000 418.000 559.250

*H* = 91.596 with 3 degrees of freedom (*P* = <0.001)

The differences in the median values among the treatment groups are greater than would be expected by chance; **there is a statistically significant difference (*P* = <0.001)**

To isolate the group or groups that differ from the others, use a multiple comparison procedure.

All Pairwise Multiple Comparison Procedures (Tukey’s Test):

**Comparison Diff of Ranks *q* *P*<0.05**

IBA 4 vs IBA 3 9657.500 12.855 Yes

IBA 4 vs IBA 2 7089.000 9.436 Yes

IBA 4 vs IBA 1 4097.500 5.454 Yes

IBA 1 vs IBA 3 5560.000 7.401 Yes

IBA 1 vs IBA 2 2991.500 3.982 Yes

IBA 2 vs IBA 3 2568.500 3.419 No

Note: The multiple comparisons on ranks do not include an adjustment for ties.

**Predominant synthesis of giant myofibrillar proteins in striated muscles of the long-tailed ground squirrel *Urocitellus undulatus* during interbout arousal** *Svetlana Popova1, Anna Ulanova1, Yulia Gritsyna1, Nikolay Salmov1, Vadim Rogachevsky2, Gulnara Mikhailova1, Alexander Bobylev1, Liya Bobyleva1, Yana Yutskevich3, Oleg Morenkov4, Nadezda Zakharova5 & Ivan Vikhlyantsev1,**

1Laboratory of the Structure and Functions of Muscle Proteins, Institute of Theoretical and Experimental Biophysics, Russian Academy of Sciences, Pushchino, Moscow Region, 142290, Russia; 2Laboratory of Signal Perception Mechanisms, Institute of Cell Biophysics, FRC PSCBR, Russian Academy of Sciences, Pushchino, Moscow Region, 142290, Russia; 3Kuban State University, Krasnodar, Krasnodar Krai, 350040, Russia; 4Laboratory of Cell Culture and Tissue Engineering, Institute of Cell Biophysics, FRC PSCBR, Russian Academy of Sciences, Pushchino, Moscow Region, 142290, Russia; 5Laboratory of Natural and Artificial Hypobiosis Mechanisms, Institute of Cell Biophysics, FRC PSCBR, Russian Academy of Sciences, Pushchino, Moscow Region, 142290, Russia

**Supplementary Table S10. Total calpain-1 content in heart of ground squirrels, %.**

| **Summer activity (SA), *n*=7** | **Hibernation (HIB), *n*=7** | **Interbout arousal (IBA), *n*=7** |
| --- | --- | --- |
| 100.0 | 54.9 | 85,9 |
| 100.0 | 60.8 | 72,4 |
| 100.0 | 74.1 | 61,3 |
| 100.0 | 75.1 | 107,3 |
| 100.0 | 82.7 | 86,8 |
| 100.0 | 86.5 | 78,3 |
| 100.0 | 92.9 | 79,3 |
| **100.0±5.9%** | **75.28±13.65%**** | **81.61±14.25%** |

Calpain-1/GAPDH content of the SA group was taken as 100% (control group). For each individual experiment, the average content of 2–3 calpain-1spots (from the control SA group) present on the blot membrane was taken into consideration. *****p* < 0.01 (vs SA).**

**Kruskal–Wallis One Way Analysis of Variance on Ranks**

**Normality Test (Shapiro–Wilk):**  Passed (*P* = 0.065)

**Equal Variance Test (Brown–Forsythe):** Passed (*P* = 0.888)

**Group N Missing Median 25% 75%**

SA 7 0 100.000 100.000 100.000

HIB 7 0 75.100 60.800 86.500

IBA 7 0 79.300 72.400 86.800

*H* = 10.812 with 2 degrees of freedom (*P* = 0.004)

The differences in the median values among the treatment groups are greater than would be expected by chance; **there is a statistically significant difference (*P* = 0.004)**

To isolate the group or groups that differ from the others, use a multiple comparison procedure.

All Pairwise Multiple Comparison Procedures (Tukey’s Test):

**Comparison Diff of Ranks *q* *P* *P*<0.050**

**SA vs HIB** 72.000 4.386 0.005 **Yes**

SA vs IBA 54.000 3.289 0.052 No

IBA vs HIB 18.000 1.096 0.718 No

Note: The multiple comparisons on ranks do not include an adjustment for ties.

**Predominant synthesis of giant myofibrillar proteins in striated muscles of the long-tailed ground squirrel *Urocitellus undulatus* during interbout arousal** *Svetlana Popova1, Anna Ulanova1, Yulia Gritsyna1, Nikolay Salmov1, Vadim Rogachevsky2, Gulnara Mikhailova1, Alexander Bobylev1, Liya Bobyleva1, Yana Yutskevich3, Oleg Morenkov4, Nadezda Zakharova5 & Ivan Vikhlyantsev1,**

1Laboratory of the Structure and Functions of Muscle Proteins, Institute of Theoretical and Experimental Biophysics, Russian Academy of Sciences, Pushchino, Moscow Region, 142290, Russia; 2Laboratory of Signal Perception Mechanisms, Institute of Cell Biophysics, FRC PSCBR, Russian Academy of Sciences, Pushchino, Moscow Region, 142290, Russia; 3Kuban State University, Krasnodar, Krasnodar Krai, 350040, Russia; 4Laboratory of Cell Culture and Tissue Engineering, Institute of Cell Biophysics, FRC PSCBR, Russian Academy of Sciences, Pushchino, Moscow Region, 142290, Russia; 5Laboratory of Natural and Artificial Hypobiosis Mechanisms, Institute of Cell Biophysics, FRC PSCBR, Russian Academy of Sciences, Pushchino, Moscow Region, 142290, Russia

**Supplementary Table S11. Content of calpain-1 autolysed fragments in heart of ground squirrels (in % of the total calpain-1 content).**

| **Summer activity (SA), *n*=7** | **Hibernation (HIB), *n*=7** | **Interbout arousal (IBA), *n*=7** |
| --- | --- | --- |
| 19.3 | 25.4 | 27.7 |
| 20.0 | 28.4 | 28.0 |
| 23.8 | 32.9 | 37.5 |
| 26.1 | 32.9 | 42.7 |
| 27.8 | 33.1 | 43.6 |
| 29.3 | 34.5 | 46.1 |
| 30.1 | 45.6 | 46.3 |
| **25.20±4.32%** | **33.26±6.32%** | **38.84±8.05%*** |

**1.32 ↑ 1.54↑**

Total calpain-1 contents were taken as 100% in each of the three groups. ****p* < 0.05 (vs SA).**

**Kruskal–Wallis One Way Analysis of Variance on Ranks**

**Normality Test (Shapiro–Wilk):**  Passed (*P* = 0.874)

**Equal Variance Test (Brown–Forsythe):** Passed (*P* = 0.529)

**Group N Missing Median 25% 75%**

SA 7 0 26.100 20.000 29.300

HIB 7 0 32.900 28.400 34.500

IBA 7 0 42.700 28.000 46.100

*H* = 8.785 with 2 degrees of freedom (*P* = 0.012)

The differences in the median values among the treatment groups are greater than would be expected by chance; **there is a statistically significant difference (*P* = 0.012)**

To isolate the group or groups that differ from the others, use a multiple comparison procedure.

All Pairwise Multiple Comparison Procedures (Tukey’s Test):

**Comparison Diff of Ranks *q* *P* *P*<0.050**

**IBA vs SA** 67.000 4.081 0.011 **Yes**

IBA vs HIB 20.000 1.218 0.665 No

HIB vs SA 47.000 2.863 0.106 No

Note: The multiple comparisons on ranks do not include an adjustment for ties.

**Predominant synthesis of giant myofibrillar proteins in striated muscles of the long-tailed ground squirrel *Urocitellus undulatus* during interbout arousal** *Svetlana Popova1, Anna Ulanova1, Yulia Gritsyna1, Nikolay Salmov1, Vadim Rogachevsky2, Gulnara Mikhailova1, Alexander Bobylev1, Liya Bobyleva1, Yana Yutskevich3, Oleg Morenkov4, Nadezda Zakharova5 & Ivan Vikhlyantsev1,**

1Laboratory of the Structure and Functions of Muscle Proteins, Institute of Theoretical and Experimental Biophysics, Russian Academy of Sciences, Pushchino, Moscow Region, 142290, Russia; 2Laboratory of Signal Perception Mechanisms, Institute of Cell Biophysics, FRC PSCBR, Russian Academy of Sciences, Pushchino, Moscow Region, 142290, Russia; 3Kuban State University, Krasnodar, Krasnodar Krai, 350040, Russia; 4Laboratory of Cell Culture and Tissue Engineering, Institute of Cell Biophysics, FRC PSCBR, Russian Academy of Sciences, Pushchino, Moscow Region, 142290, Russia; 5Laboratory of Natural and Artificial Hypobiosis Mechanisms, Institute of Cell Biophysics, FRC PSCBR, Russian Academy of Sciences, Pushchino, Moscow Region, 142290, Russia

**Supplementary Table S12. Total calpain-1 content in m. longissimus dorsi of ground squirrels, %.**

| **Summer activity (SA), *n*=7** | **Hibernation (HIB), *n*=7** | **Interbout arousal (IBA), *n*=7** |
| --- | --- | --- |
| 100.0 | 95.4 | 77.6 |
| 100.0 | 98.5 | 94.2 |
| 100.0 | 99.4 | 106.2 |
| 100.0 | 104.3 | 106.6 |
| 100.0 | 106.5 | 107.3 |
| 100.0 | 108.1 | 111.3 |
| 100.0 | 118.0 | 131.8 |
| **100.0±4.0%** | **104.31±7.57%** | **105.0±16.50%** |

Calpain-1/GAPDH content of the SA group was taken as 100% (control group). For each individual experiment, the average content of 2–3 calpain-1 spots (from the control SA group) present on the blot membrane was taken into consideration.

**Kruskal–Wallis One Way Analysis of Variance on Ranks**

**Normality Test (Shapiro–Wilk):**  Failed (*P* < 0.050)

**Group N Missing Median 25% 75%**

SA 7 0 100.000 100.000 100.000

HIB 7 0 104.300 98.500 108.100

IBA 7 0 106.600 94.200 111.300

*H* = 1.409 with 2 degrees of freedom (*P* = 0.494)

The differences in the median values among the treatment groups are not great enough to exclude the possibility that the difference is due to random sampling variability; **there is no statistically significant difference (*P* = 0.494)**

**Predominant synthesis of giant myofibrillar proteins in striated muscles of the long-tailed ground squirrel *Urocitellus undulatus* during interbout arousal** *Svetlana Popova1, Anna Ulanova1, Yulia Gritsyna1, Nikolay Salmov1, Vadim Rogachevsky2, Gulnara Mikhailova1, Alexander Bobylev1, Liya Bobyleva1, Yana Yutskevich3, Oleg Morenkov4, Nadezda Zakharova5 & Ivan Vikhlyantsev1,**

1Laboratory of the Structure and Functions of Muscle Proteins, Institute of Theoretical and Experimental Biophysics, Russian Academy of Sciences, Pushchino, Moscow Region, 142290, Russia; 2Laboratory of Signal Perception Mechanisms, Institute of Cell Biophysics, FRC PSCBR, Russian Academy of Sciences, Pushchino, Moscow Region, 142290, Russia; 3Kuban State University, Krasnodar, Krasnodar Krai, 350040, Russia; 4Laboratory of Cell Culture and Tissue Engineering, Institute of Cell Biophysics, FRC PSCBR, Russian Academy of Sciences, Pushchino, Moscow Region, 142290, Russia; 5Laboratory of Natural and Artificial Hypobiosis Mechanisms, Institute of Cell Biophysics, FRC PSCBR, Russian Academy of Sciences, Pushchino, Moscow Region, 142290, Russia

**Supplementary Table S13. Content of calpain-1 autolysed fragments in m. longissimus dorsi of ground squirrels (in % of the total calpain-1 content).**

| **Summer activity (SA), *n*=7** | **Hibernation (HIB), *n*=7** | **Interbout arousal (IBA), *n*=7** |
| --- | --- | --- |
| 13.3 | 25.1 | 19.7 |
| 15.3 | 30.6 | 32.5 |
| 14.5 | 32.7 | 34.5 |
| 16.2 | 32.8 | 35.1 |
| 18.8 | 34.2 | 37.1 |
| 27.7 | 37.7 | 44.0 |
| 31.5 | 39.0 | 45.0 |
| **19.6±7.1%** | **33.2±4.6%*** | **35.4±8.4%**** |

**1.69↑ 1.8↑**

Total calpain-1 contents were taken as 100% in each of the three groups. ****p* < 0.05, ***p* < 0.01 (vs SA).**

**Kruskal–Wallis One Way Analysis of Variance on Ranks**

**Normality Test (Shapiro–Wilk):**  Passed (*P* = 0.688)

**Equal Variance Test (Brown–Forsythe):** Passed (*P* = 0.624)

**Group N Missing Median 25% 75%**

SA 7 0 16.200 14.500 27.700

HIB 7 0 32.800 30.600 37.700

IBA 7 0 35.100 32.500 44.000

*H* = 11.139 with 2 degrees of freedom (*P* = 0.004)

The differences in the median values among the treatment groups are greater than would be expected by chance; **there is a statistically significant difference (*P* = 0.004)**

To isolate the group or groups that differ from the others, use a multiple comparison procedure.

All Pairwise Multiple Comparison Procedures (Tukey’s Test):

**Comparison Diff of Ranks *q* *P* *P*<0.050**

**IBA vs SA** 73.000 4.447 0.005 **Yes**

IBA vs HIB 14.000 0.853 0.818 No

**HIB vs SA** 59.000 3.594 0.030 **Yes**

Note: The multiple comparisons on ranks do not include an adjustment for ties.

**Predominant synthesis of giant myofibrillar proteins in striated muscles of the long-tailed ground squirrel *Urocitellus undulatus* during interbout arousal** *Svetlana Popova1, Anna Ulanova1, Yulia Gritsyna1, Nikolay Salmov1, Vadim Rogachevsky2, Gulnara Mikhailova1, Alexander Bobylev1, Liya Bobyleva1, Yana Yutskevich3, Oleg Morenkov4, Nadezda Zakharova5 & Ivan Vikhlyantsev1,**

1Laboratory of the Structure and Functions of Muscle Proteins, Institute of Theoretical and Experimental Biophysics, Russian Academy of Sciences, Pushchino, Moscow Region, 142290, Russia; 2Laboratory of Signal Perception Mechanisms, Institute of Cell Biophysics, FRC PSCBR, Russian Academy of Sciences, Pushchino, Moscow Region, 142290, Russia; 3Kuban State University, Krasnodar, Krasnodar Krai, 350040, Russia; 4Laboratory of Cell Culture and Tissue Engineering, Institute of Cell Biophysics, FRC PSCBR, Russian Academy of Sciences, Pushchino, Moscow Region, 142290, Russia; 5Laboratory of Natural and Artificial Hypobiosis Mechanisms, Institute of Cell Biophysics, FRC PSCBR, Russian Academy of Sciences, Pushchino, Moscow Region, 142290, Russia

**Supplementary Table S14. Calpastatin content in heart of ground squirrels, %.**

| **Summer activity (SA), *n*=7** | **Hibernation (HIB), *n*=7** | **Interbout arousal (IBA), *n*=7** |
| --- | --- | --- |
| 100.0 | 69.1 | 72.1 |
| 100.0 | 71.7 | 73.5 |
| 100.0 | 72.5 | 87.7 |
| 100.0 | 74.8 | 93.0 |
| 100.0 | 78.5 | 93.9 |
| 100.0 | 92.0 | 114.2 |
| 100.0 | 107.4 | 118.7 |
| **100.0±8.5%** | **80.86±13.92%*** | **93.3±18.05%** |

Calpastatin/GAPDH content of the SA group was taken as 100% (control group). For each individual experiment, the average content of 2–3 calpastatin spots (from the control SA group) present on the blot membrane was taken into consideration. ****p* < 0.05 (vs SA).**

**Kruskal–Wallis One Way Analysis of Variance on Ranks**

**Normality Test (Shapiro–Wilk):**  Failed (*P* < 0.050)

**Group N Missing Median 25% 75%**

SA 7 0 100.000 100.000 100.000

HIB 7 0 74.800 71.700 92.000

IBA 7 0 93.000 73.500 114.200

*H* = 6.261 with 2 degrees of freedom (*P* = 0.044)

The differences in the median values among the treatment groups are greater than would be expected by chance; **there is a statistically significant difference (*P* = 0.044)**

To isolate the group or groups that differ from the others, use a multiple comparison procedure.

All Pairwise Multiple Comparison Procedures (Tukey’s Test):

**Comparison Diff of Ranks *q* *P* *P*<0.050**

**SA vs HIB** 57.000 3.472 0.037 **Yes**

SA vs IBA 27.000 1.645 0.475 No

IBA vs HIB 30.000 1.827 0.400 No

Note: The multiple comparisons on ranks do not include an adjustment for ties.

**Predominant synthesis of giant myofibrillar proteins in striated muscles of the long-tailed ground squirrel *Urocitellus undulatus* during interbout arousal** *Svetlana Popova1, Anna Ulanova1, Yulia Gritsyna1, Nikolay Salmov1, Vadim Rogachevsky2, Gulnara Mikhailova1, Alexander Bobylev1, Liya Bobyleva1, Yana Yutskevich3, Oleg Morenkov4, Nadezda Zakharova5 & Ivan Vikhlyantsev1,**

1Laboratory of the Structure and Functions of Muscle Proteins, Institute of Theoretical and Experimental Biophysics, Russian Academy of Sciences, Pushchino, Moscow Region, 142290, Russia; 2Laboratory of Signal Perception Mechanisms, Institute of Cell Biophysics, FRC PSCBR, Russian Academy of Sciences, Pushchino, Moscow Region, 142290, Russia; 3Kuban State University, Krasnodar, Krasnodar Krai, 350040, Russia; 4Laboratory of Cell Culture and Tissue Engineering, Institute of Cell Biophysics, FRC PSCBR, Russian Academy of Sciences, Pushchino, Moscow Region, 142290, Russia; 5Laboratory of Natural and Artificial Hypobiosis Mechanisms, Institute of Cell Biophysics, FRC PSCBR, Russian Academy of Sciences, Pushchino, Moscow Region, 142290, Russia

**Supplementary Table S15. Calpastatin content in m. longissimus dorsi**

**of ground squirrels, %.**

| **Summer activity (SA), *n*=7** | **Hibernation (HIB), *n*=7** | **Interbout arousal (IBA), *n*=7** |
| --- | --- | --- |
| 100.0 | 93.4 | 88.2 |
| 100.0 | 94.2 | 94.9 |
| 100.0 | 95.4 | 98.0 |
| 100.0 | 97.1 | 100.0 |
| 100.0 | 104.2 | 103.9 |
| 100.0 | 117.7 | 125.3 |
| 100.0 | 127.7 | 137.9 |
| **100.0±5.8%** | **104.24±13.4%** | **106.88±17.93%** |

Calpastatin/GAPDH content of the SA group was taken as 100% (control group). For each individual experiment, the average content of 2–3 Calpastatin spots (from the control SA group) present on the blot membrane was taken into consideration.

**Kruskal–Wallis One Way Analysis of Variance on Ranks**

**Normality Test (Shapiro–Wilk):**  Failed (*P* < 0.050)

**Group N Missing Median 25% 75%**

SA 7 0 100.000 100.000 100.000

HIB 7 0 97.100 94.200 117.700

IBA 7 0 100.000 94.900 125.300

*H* = 0.214 with 2 degrees of freedom (*P* = 0.899)

The differences in the median values among the treatment groups are not great enough to exclude the possibility that the difference is due to random sampling variability; **there is no statistically significant difference (*P* = 0.899)**

**Predominant synthesis of giant myofibrillar proteins in striated muscles of the long-tailed ground squirrel *Urocitellus undulatus* during interbout arousal** *Svetlana Popova1, Anna Ulanova1, Yulia Gritsyna1, Nikolay Salmov1, Vadim Rogachevsky2, Gulnara Mikhailova1, Alexander Bobylev1, Liya Bobyleva1, Yana Yutskevich3, Oleg Morenkov4, Nadezda Zakharova5 & Ivan Vikhlyantsev1,**

1Laboratory of the Structure and Functions of Muscle Proteins, Institute of Theoretical and Experimental Biophysics, Russian Academy of Sciences, Pushchino, Moscow Region, 142290, Russia; 2Laboratory of Signal Perception Mechanisms, Institute of Cell Biophysics, FRC PSCBR, Russian Academy of Sciences, Pushchino, Moscow Region, 142290, Russia; 3Kuban State University, Krasnodar, Krasnodar Krai, 350040, Russia; 4Laboratory of Cell Culture and Tissue Engineering, Institute of Cell Biophysics, FRC PSCBR, Russian Academy of Sciences, Pushchino, Moscow Region, 142290, Russia; 5Laboratory of Natural and Artificial Hypobiosis Mechanisms, Institute of Cell Biophysics, FRC PSCBR, Russian Academy of Sciences, Pushchino, Moscow Region, 142290, Russia

**Supplementary Table S16. Hsp 90 content in heart of ground squirrels, %.**

| **Summer activity (SA), *n*=7** | **Hibernation (HIB), *n*=7** | **Interbout arousal (IBA), *n*=7** |
| --- | --- | --- |
| 100.0 | 79.9 | 79.2 |
| 100.0 | 91.2 | 85.9 |
| 100.0 | 102.4 | 92.8 |
| 100.0 | 120.1 | 105.4 |
| 100.0 | 124.3 | 116.1 |
| 100.0 | 125.2 | 122.7 |
| 100.0 | 142.9 | 147.3 |
| **100.0±6.7%** | **112.3±21.9%** | **107.1±23.7%** |

HSP 90/GAPDH content of the SA group was taken as 100% (control group). For each individual experiment, the average content of 2–3 HSP 90 spots (from the control SA group) present on the blot membrane was taken into consideration.

**Kruskal–Wallis One Way Analysis of Variance on Ranks**

**Normality Test (Shapiro–Wilk):**  Passed (*P* = 0.378)

**Equal Variance Test (Brown–Forsythe):** Passed (*P* = 0.838)

**Group N Missing Median 25% 75%**

SA 7 0 100.000 100.000 100.000

HIB 7 0 120.100 91.200 125.200

IBA 7 0 105.400 85.900 122.700

*H* = 1.625 with 2 degrees of freedom (*P* = 0.444)

The differences in the median values among the treatment groups are not great enough to exclude the possibility that the difference is due to random sampling variability; **there is no statistically significant difference (*P* = 0.444)**

**Predominant synthesis of giant myofibrillar proteins in striated muscles of the long-tailed ground squirrel *Urocitellus undulatus* during interbout arousal** *Svetlana Popova1, Anna Ulanova1, Yulia Gritsyna1, Nikolay Salmov1, Vadim Rogachevsky2, Gulnara Mikhailova1, Alexander Bobylev1, Liya Bobyleva1, Yana Yutskevich3, Oleg Morenkov4, Nadezda Zakharova5 & Ivan Vikhlyantsev1,**

1Laboratory of the Structure and Functions of Muscle Proteins, Institute of Theoretical and Experimental Biophysics, Russian Academy of Sciences, Pushchino, Moscow Region, 142290, Russia; 2Laboratory of Signal Perception Mechanisms, Institute of Cell Biophysics, FRC PSCBR, Russian Academy of Sciences, Pushchino, Moscow Region, 142290, Russia; 3Kuban State University, Krasnodar, Krasnodar Krai, 350040, Russia; 4Laboratory of Cell Culture and Tissue Engineering, Institute of Cell Biophysics, FRC PSCBR, Russian Academy of Sciences, Pushchino, Moscow Region, 142290, Russia; 5Laboratory of Natural and Artificial Hypobiosis Mechanisms, Institute of Cell Biophysics, FRC PSCBR, Russian Academy of Sciences, Pushchino, Moscow Region, 142290, Russia

**Supplementary Table S17. Hsp 90 content in m. longissimus dorsi of ground squirrels, %.**

| **Summer activity (SA), *n*=7** | **Hibernation (HIB), *n*=7** | **Interbout arousal (IBA), *n*=7** |
| --- | --- | --- |
| 100.0 | 93.5 | 89.0 |
| 100.0 | 97.8 | 97.8 |
| 100.0 | 98.9 | 99.3 |
| 100.0 | 104.1 | 102.9 |
| 100.0 | 104.7 | 111.1 |
| 100.0 | 126.7 | 111.9 |
| 100.0 | 130.8 | 126.2 |
| **100.0±8.8%** | **108.1±14.7%** | **105.5±12.1%** |

HSP 90/GAPDH content of the SA group was taken as 100% (control group). For each individual experiment, the average content of 2–3 HSP 90 spots (from the control SA group) present on the blot membrane was taken into consideration.

**Kruskal–Wallis One Way Analysis of Variance on Ranks**

**Normality Test (Shapiro–Wilk):**  Failed (*P* < 0.050)

**Group N Missing Median 25% 75%**

SA 7 0 100.000 100.000 100.000

HIB 7 0 104.100 97.800 126.700

IBA 7 0 102.900 97.800 111.900

*H* = 0.314 with 2 degrees of freedom (*P* = 0.855)

The differences in the median values among the treatment groups are not great enough to exclude the possibility that the difference is due to random sampling variability; **there is no statistically significant difference (*P* = 0.855)**

**Predominant synthesis of giant myofibrillar proteins in striated muscles of the long-tailed ground squirrel *Urocitellus undulatus* during interbout arousal** *Svetlana Popova1, Anna Ulanova1, Yulia Gritsyna1, Nikolay Salmov1, Vadim Rogachevsky2, Gulnara Mikhailova1, Alexander Bobylev1, Liya Bobyleva1, Yana Yutskevich3, Oleg Morenkov4, Nadezda Zakharova5 & Ivan Vikhlyantsev1,**

1Laboratory of the Structure and Functions of Muscle Proteins, Institute of Theoretical and Experimental Biophysics, Russian Academy of Sciences, Pushchino, Moscow Region, 142290, Russia; 2Laboratory of Signal Perception Mechanisms, Institute of Cell Biophysics, FRC PSCBR, Russian Academy of Sciences, Pushchino, Moscow Region, 142290, Russia; 3Kuban State University, Krasnodar, Krasnodar Krai, 350040, Russia; 4Laboratory of Cell Culture and Tissue Engineering, Institute of Cell Biophysics, FRC PSCBR, Russian Academy of Sciences, Pushchino, Moscow Region, 142290, Russia; 5Laboratory of Natural and Artificial Hypobiosis Mechanisms, Institute of Cell Biophysics, FRC PSCBR, Russian Academy of Sciences, Pushchino, Moscow Region, 142290, Russia

**Supplementary Table S18. T1 content in the heart of ground squirrels, %.**

| **Summer activity (SA), *n*=7** | **Hibernation (HIB), *n*=7** | **Interbout arousal (IBA), *n*=7** |
| --- | --- | --- |
| 100.0 | 73.4 | 78.0 |
| 100.0 | 78.7 | 80.9 |
| 100.0 | 81.6 | 84.9 |
| 100.0 | 84.6 | 96.3 |
| 100.0 | 85.9 | 101.5 |
| 100.0 | 91.7 | 108.0 |
| 100.0 | 92.3 | 121.3 |
| **100.0±7.6%** | **84.03±6.8%*** | **95.8±15.75%** |

T1/MyHC content of the SA group was taken as 100% (control group). For each individual experiment, the average content of 2–3 T1 bands (from the control SA group) present on the gel was taken into consideration. ****p* < 0.05 (vs SA).**

**Kruskal–Wallis One Way Analysis of Variance on Ranks**

**Normality Test (Shapiro–Wilk):**  Passed (*P* = 0.068)

**Equal Variance Test (Brown–Forsythe):** Failed (*P* < 0.050)

**Group N Missing Median 25% 75%**

SA 7 0 100.000 100.000 100.000

HIB 7 0 84.600 78.700 91.700

IBA 7 0 96.300 80.900 108.000

*H* = 7.925 with 2 degrees of freedom (*P* = 0.019)

The differences in the median values among the treatment groups are greater than would be expected by chance; **there is a statistically significant difference (*P* = 0.019)**

To isolate the group or groups that differ from the others, use a multiple comparison procedure.

All Pairwise Multiple Comparison Procedures (Tukey’s Test):

**Comparison Diff of Ranks *q* *P* *P*<0.050**

**SA vs HIB** 63.000 3.838 0.018 **Yes**

SA vs IBA 21.000 1.279 0.637 No

IBA vs HIB 42.000 2.558 0.167 No

Note: The multiple comparisons on ranks do not include an adjustment for ties.

**Predominant synthesis of giant myofibrillar proteins in striated muscles of the long-tailed ground squirrel *Urocitellus undulatus* during interbout arousal** *Svetlana Popova1, Anna Ulanova1, Yulia Gritsyna1, Nikolay Salmov1, Vadim Rogachevsky2, Gulnara Mikhailova1, Alexander Bobylev1, Liya Bobyleva1, Yana Yutskevich3, Oleg Morenkov4, Nadezda Zakharova5 & Ivan Vikhlyantsev1,**

1Laboratory of the Structure and Functions of Muscle Proteins, Institute of Theoretical and Experimental Biophysics, Russian Academy of Sciences, Pushchino, Moscow Region, 142290, Russia; 2Laboratory of Signal Perception Mechanisms, Institute of Cell Biophysics, FRC PSCBR, Russian Academy of Sciences, Pushchino, Moscow Region, 142290, Russia; 3Kuban State University, Krasnodar, Krasnodar Krai, 350040, Russia; 4Laboratory of Cell Culture and Tissue Engineering, Institute of Cell Biophysics, FRC PSCBR, Russian Academy of Sciences, Pushchino, Moscow Region, 142290, Russia; 5Laboratory of Natural and Artificial Hypobiosis Mechanisms, Institute of Cell Biophysics, FRC PSCBR, Russian Academy of Sciences, Pushchino, Moscow Region, 142290, Russia

**Supplementary Table S19. T2 content in heart of ground squirrels, %.**

| **Summer activity (SA), *n*=7** | **Hibernation (HIB), *n*=7** | **Interbout arousal (IBA), *n*=7** |
| --- | --- | --- |
| 100.0 | 46.2 | 72.4 |
| 100.0 | 56.3 | 78.6 |
| 100.0 | 60.5 | 79.5 |
| 100.0 | 75.0 | 82.6 |
| 100.0 | 77.2 | 95.9 |
| 100.0 | 88.7 | 102.0 |
| 100.0 | 94.3 | 129.2 |
| **100.0±10.6%** | **71.17±17.57%**** | **91.45±19.6%** |

T2/MyHC content of the SA group was taken as 100% (control group). For each individual experiment, the average content of 2–3 T2 bands (from the control SA group) present on the gel was taken into consideration. *****p* < 0.01 (vs SA).**

**Kruskal–Wallis One Way Analysis of Variance on Ranks**

**Normality Test (Shapiro–Wilk):**  Passed (*P* = 0.221)

**Equal Variance Test (Brown–Forsythe):** Passed (*P* = 0.990)

**Group N Missing Median 25% 75%**

AS 7 0 100.000 100.000 100.000

HIB 7 0 75.000 56.300 88.700

AW 7 0 82.600 78.600 102.000

*H* = 10.635 with 2 degrees of freedom (*P* = 0.005)

The differences in the median values among the treatment groups are greater than would be expected by chance; **there is a statistically significant difference (*P* = 0.005)**

To isolate the group or groups that differ from the others, use a multiple comparison procedure.

All Pairwise Multiple Comparison Procedures (Tukey’s Test):

**Comparison Diff of Ranks *q* *P* *P*<0.050**

**SA vs HIB** 74.000 4.508 0.004 **Yes**

SA vs IBA 31.000 1.888 0.376 No

IBA vs HIB 43.000 2.619 0.153 No

**Predominant synthesis of giant myofibrillar proteins in striated muscles of the long-tailed ground squirrel *Urocitellus undulatus* during interbout arousal** *Svetlana Popova1, Anna Ulanova1, Yulia Gritsyna1, Nikolay Salmov1, Vadim Rogachevsky2, Gulnara Mikhailova1, Alexander Bobylev1, Liya Bobyleva1, Yana Yutskevich3, Oleg Morenkov4, Nadezda Zakharova5 & Ivan Vikhlyantsev1,**

1Laboratory of the Structure and Functions of Muscle Proteins, Institute of Theoretical and Experimental Biophysics, Russian Academy of Sciences, Pushchino, Moscow Region, 142290, Russia; 2Laboratory of Signal Perception Mechanisms, Institute of Cell Biophysics, FRC PSCBR, Russian Academy of Sciences, Pushchino, Moscow Region, 142290, Russia; 3Kuban State University, Krasnodar, Krasnodar Krai, 350040, Russia; 4Laboratory of Cell Culture and Tissue Engineering, Institute of Cell Biophysics, FRC PSCBR, Russian Academy of Sciences, Pushchino, Moscow Region, 142290, Russia; 5Laboratory of Natural and Artificial Hypobiosis Mechanisms, Institute of Cell Biophysics, FRC PSCBR, Russian Academy of Sciences, Pushchino, Moscow Region, 142290, Russia

**Supplementary Table S20. T1 content in m. longissimus dorsi of ground squirrels, %.**

| **Summer activity (SA), *n*=7** | **Hibernation (HIB), *n*=7** | **Interbout arousal (IBA), *n*=7** |
| --- | --- | --- |
| 100.0 | 76.1 | 85.4 |
| 100.0 | 81.0 | 93.1 |
| 100.0 | 83.3 | 96.4 |
| 100.0 | 84.2 | 98.6 |
| 100.0 | 88.2 | 100.5 |
| 100.0 | 92.3 | 103.3 |
| 100.0 | 94.1 | 111.3 |
| **100.0±9.0%** | **85.6±6.4%**** | **98.4±8.1%#** |

T1/MyHC content of the SA group was taken as 100% (control group). For each individual experiment, the average content of 2–3 T1 bands (from the control SA group) present on the gel was taken into consideration. *****p* < 0.01 (vs SA). #*p* < 0.05 (IBA vs HIB).**

**Kruskal–Wallis One Way Analysis of Variance on Ranks**

**Normality Test (Shapiro–Wilk):**  Passed (*P* = 0.211)

**Equal Variance Test (Brown–Forsythe):** Passed (*P* = 0.698)

**Group N Missing Median 25% 75%**

SA 7 0 100.000 100.000 100.000

HIB 7 0 84.200 81.000 92.300

IBA 7 0 98.600 93.100 103.300

*H* = 11.929 with 2 degrees of freedom (*P* = 0.003)

The differences in the median values among the treatment groups are greater than would be expected by chance; **there is a statistically significant difference (*P* = 0.003)**

To isolate the group or groups that differ from the others, use a multiple comparison procedure.

All Pairwise Multiple Comparison Procedures (Tukey’s Test):

**Comparison Diff of Ranks *q* *P* *P*<0.050**

**SA vs HIB** 73.000 4.447 0.005 **Yes**

SA vs IBA 11.000 0.670 0.884 No

**IBA vs HIB** 62.000 3.777 0.021 **Yes**

Note: The multiple comparisons on ranks do not include an adjustment for ties.

**Predominant synthesis of giant myofibrillar proteins in striated muscles of the long-tailed ground squirrel *Urocitellus undulatus* during interbout arousal** *Svetlana Popova1, Anna Ulanova1, Yulia Gritsyna1, Nikolay Salmov1, Vadim Rogachevsky2, Gulnara Mikhailova1, Alexander Bobylev1, Liya Bobyleva1, Yana Yutskevich3, Oleg Morenkov4, Nadezda Zakharova5 & Ivan Vikhlyantsev1,**

1Laboratory of the Structure and Functions of Muscle Proteins, Institute of Theoretical and Experimental Biophysics, Russian Academy of Sciences, Pushchino, Moscow Region, 142290, Russia; 2Laboratory of Signal Perception Mechanisms, Institute of Cell Biophysics, FRC PSCBR, Russian Academy of Sciences, Pushchino, Moscow Region, 142290, Russia; 3Kuban State University, Krasnodar, Krasnodar Krai, 350040, Russia; 4Laboratory of Cell Culture and Tissue Engineering, Institute of Cell Biophysics, FRC PSCBR, Russian Academy of Sciences, Pushchino, Moscow Region, 142290, Russia; 5Laboratory of Natural and Artificial Hypobiosis Mechanisms, Institute of Cell Biophysics, FRC PSCBR, Russian Academy of Sciences, Pushchino, Moscow Region, 142290, Russia

**Supplementary Table S21. T2 content in m. longissimus dorsi of ground squirrels, %.**

| **Summer activity (SA), *n*=7** | **Hibernation (HIB), *n*=7** | **Interbout arousal (IBA), *n*=7** |
| --- | --- | --- |
| 100.0 | 28.6 | 62.5 |
| 100.0 | 33.5 | 68.5 |
| 100.0 | 38.9 | 75.5 |
| 100.0 | 45.8 | 97.5 |
| 100.0 | 48.8 | 101.3 |
| 100.0 | 62.5 | 119.9 |
| 100.0 | 73.5 | 123.8 |
| **100.0±14.8%** | **47.4±15.98%**** | **92.7±24.48%#** |

T2/MyHC content of the SA group was taken as 100% (control group). For each individual experiment, the average content of 2–3 T2 bands (from the control SA group) present on the gel was taken into consideration. *****p* < 0.01 (vs SA). #*p* < 0.05 (IBA vs HIB).**

**Kruskal–Wallis One Way Analysis of Variance on Ranks**

**Normality Test (Shapiro–Wilk):**  Passed (*P* = 0.193)

**Equal Variance Test (Brown–Forsythe):** Passed (*P* = 0.135)

**Group N Missing Median 25% 75%**

SA 7 0 100.000 100.000 100.000

HIB 7 0 45.800 33.500 62.500

IBA 7 0 97.500 68.500 119.900

*H* = 12.671 with 2 degrees of freedom (*P* = 0.002)

The differences in the median values among the treatment groups are greater than would be expected by chance; **there is a statistically significant difference (*P* = 0.002)**

To isolate the group or groups that differ from the others, use a multiple comparison procedure.

All Pairwise Multiple Comparison Procedures (Tukey’s Test):

**Comparison Diff of Ranks *q* *P* *P*<0.050**

**SA vs HIB** 74.500 4.538 0.004 **Yes**

SA vs IBA 9.500 0.579 0.912 No

**IBA vs HIB** 65.000 3.959 0.014 **Yes**

**Predominant synthesis of giant myofibrillar proteins in striated muscles of the long-tailed ground squirrel *Urocitellus undulatus* during interbout arousal** *Svetlana Popova1, Anna Ulanova1, Yulia Gritsyna1, Nikolay Salmov1, Vadim Rogachevsky2, Gulnara Mikhailova1, Alexander Bobylev1, Liya Bobyleva1, Yana Yutskevich3, Oleg Morenkov4, Nadezda Zakharova5 & Ivan Vikhlyantsev1,**

1Laboratory of the Structure and Functions of Muscle Proteins, Institute of Theoretical and Experimental Biophysics, Russian Academy of Sciences, Pushchino, Moscow Region, 142290, Russia; 2Laboratory of Signal Perception Mechanisms, Institute of Cell Biophysics, FRC PSCBR, Russian Academy of Sciences, Pushchino, Moscow Region, 142290, Russia; 3Kuban State University, Krasnodar, Krasnodar Krai, 350040, Russia; 4Laboratory of Cell Culture and Tissue Engineering, Institute of Cell Biophysics, FRC PSCBR, Russian Academy of Sciences, Pushchino, Moscow Region, 142290, Russia; 5Laboratory of Natural and Artificial Hypobiosis Mechanisms, Institute of Cell Biophysics, FRC PSCBR, Russian Academy of Sciences, Pushchino, Moscow Region, 142290, Russia

**Supplementary Table S22. Nebulin content in m. longissimus dorsi of ground squirrels, %.**

| **Summer activity (SA), *n*=7** | **Hibernation (HIB), *n*=7** | **Interbout arousal (IBA), *n*=7** |
| --- | --- | --- |
| 100.0 | 85.6 | 81.1 |
| 100.0 | 90.0 | 83.8 |
| 100.0 | 93.4 | 93.0 |
| 100.0 | 97.0 | 94.5 |
| 100.0 | 102.3 | 105.0 |
| 100.0 | 105.8 | 110.2 |
| 100.0 | 107.8 | 116.6 |
| **100.0±9.2%** | **97.4±8.3%** | **97.7±13.34%** |

Nebulin/MyHC content of the SA group was taken as 100% (control group). For each individual experiment, the average content of 2–3 nebulin bands (from the control SA group) present on the gel was taken into consideration.

**Kruskal–Wallis One Way Analysis of Variance on Ranks**

**Normality Test (Shapiro–Wilk):**  Passed (*P* = 0.501)

**Equal Variance Test (Brown–Forsythe):** Passed (*P* = 0.171)

**Group N Missing Median 25% 75%**

SA 7 0 100.000 100.000 100.000

HIB 7 0 97.000 90.000 105.800

IBA 7 0 94.500 83.800 110.200

*H* = 0.285 with 2 degrees of freedom (*P* = 0.867)

The differences in the median values among the treatment groups are not great enough to exclude the possibility that the difference is due to random sampling variability; **there is no statistically significant difference (*P* = 0.867)**

**Predominant synthesis of giant myofibrillar proteins in striated muscles of the long-tailed ground squirrel *Urocitellus undulatus* during interbout arousal** *Svetlana Popova1, Anna Ulanova1, Yulia Gritsyna1, Nikolay Salmov1, Vadim Rogachevsky2, Gulnara Mikhailova1, Alexander Bobylev1, Liya Bobyleva1, Yana Yutskevich3, Oleg Morenkov4, Nadezda Zakharova5 & Ivan Vikhlyantsev1,**

1Laboratory of the Structure and Functions of Muscle Proteins, Institute of Theoretical and Experimental Biophysics, Russian Academy of Sciences, Pushchino, Moscow Region, 142290, Russia; 2Laboratory of Signal Perception Mechanisms, Institute of Cell Biophysics, FRC PSCBR, Russian Academy of Sciences, Pushchino, Moscow Region, 142290, Russia; 3Kuban State University, Krasnodar, Krasnodar Krai, 350040, Russia; 4Laboratory of Cell Culture and Tissue Engineering, Institute of Cell Biophysics, FRC PSCBR, Russian Academy of Sciences, Pushchino, Moscow Region, 142290, Russia; 5Laboratory of Natural and Artificial Hypobiosis Mechanisms, Institute of Cell Biophysics, FRC PSCBR, Russian Academy of Sciences, Pushchino, Moscow Region, 142290, Russia

**Supplementary Table S23. T1 phosphorylation level in the heart of ground squirrels, %.**

| **Summer activity (SA), *n*=5** | **Hibernation (HIB), *n*=5** | **Interbout arousal (IBA), *n*=5** |
| --- | --- | --- |
| 100.0 | 62.3 | 74.6 |
| 100.0 | 71.9 | 82.5 |
| 100.0 | 78.9 | 90.3 |
| 100.0 | 83.6 | 91.1 |
| 100.0 | 88.5 | 112.5 |
| **100.0±12.4%** | **77.04±10.26%*** | **90.2±14.15%** |

T1 phosphorylation level/T1 protein level of the SA group was taken as 100% (control group). For each individual experiment, the average content of 2–3 T1 bands (from the control SA group) present on the gel was taken into consideration. The native level of protein phosphorylation was estimated in the gels using the fluorescent dye Pro-Q Diamond (Thermo Fisher Scientific). ****p* < 0.05 (vs SA).**

**Kruskal–Wallis One Way Analysis of Variance on Ranks**

**Normality Test (Shapiro–Wilk):**  Passed (*P* = 0.098)

**Equal Variance Test (Brown–Forsythe):** Passed (*P* = 0.700)

**Group N Missing Median 25% 75%**

SA 5 0 100.000 100.000 100.000

HIB 5 0 78.900 67.100 86.050

IBA 5 0 90.300 78.550 101.800

*H* = 8.296 with 2 degrees of freedom (*P* = 0.016)

The differences in the median values among the treatment groups are greater than would be expected by chance; **there is a statistically significant difference (*P* = 0.016)**

To isolate the group or groups that differ from the others, use a multiple comparison procedure.

All Pairwise Multiple Comparison Procedures (Tukey’s Test):

**Comparison Diff of Ranks *q* *P* *P*<0.050**

**SA vs HIB** 40.000 4.000 0.013 **Yes**

SA vs IBA 20.000 2.000 0.333 No

IBA vs HIB 20.000 2.000 0.333 No

Note: The multiple comparisons on ranks do not include an adjustment for ties.

**Predominant synthesis of giant myofibrillar proteins in striated muscles of the long-tailed ground squirrel *Urocitellus undulatus* during interbout arousal** *Svetlana Popova1, Anna Ulanova1, Yulia Gritsyna1, Nikolay Salmov1, Vadim Rogachevsky2, Gulnara Mikhailova1, Alexander Bobylev1, Liya Bobyleva1, Yana Yutskevich3, Oleg Morenkov4, Nadezda Zakharova5 & Ivan Vikhlyantsev1,**

1Laboratory of the Structure and Functions of Muscle Proteins, Institute of Theoretical and Experimental Biophysics, Russian Academy of Sciences, Pushchino, Moscow Region, 142290, Russia; 2Laboratory of Signal Perception Mechanisms, Institute of Cell Biophysics, FRC PSCBR, Russian Academy of Sciences, Pushchino, Moscow Region, 142290, Russia; 3Kuban State University, Krasnodar, Krasnodar Krai, 350040, Russia; 4Laboratory of Cell Culture and Tissue Engineering, Institute of Cell Biophysics, FRC PSCBR, Russian Academy of Sciences, Pushchino, Moscow Region, 142290, Russia; 5Laboratory of Natural and Artificial Hypobiosis Mechanisms, Institute of Cell Biophysics, FRC PSCBR, Russian Academy of Sciences, Pushchino, Moscow Region, 142290, Russia

**Supplementary Table S24. T1 phosphorylation level in m. longissimus dorsi of ground squirrels, %.**

| **Summer activity (SA), *n*=5** | **Hibernation (HIB), *n*=5** | **Interbout arousal (IBA), *n*=5** |
| --- | --- | --- |
| 100.0 | 119.4 | 93.6 |
| 100.0 | 122.7 | 110.2 |
| 100.0 | 125.5 | 113.9 |
| 100.0 | 142.8 | 122.6 |
| 100.0 | 147.8 | 133.3 |
| **100.0±11.0%** | **131.64±12.78%*** | **114.72±14.79%** |

T1 phosphorylation level/T1 protein level of the SA group was taken as 100% (control group). For each individual experiment, the average content of 2–3 T1 bands (from the control SA group) present on the gel was taken into consideration. The native level of protein phosphorylation was estimated in the gels using the fluorescent dye Pro-Q Diamond (Thermo Fisher Scientific). ****p* < 0.05 (vs SA).**

**Kruskal–Wallis One Way Analysis of Variance on Ranks**

**Normality Test (Shapiro–Wilk):**  Passed (*P* = 0.581)

**Equal Variance Test (Brown–Forsythe):** Passed (*P* = 0.888)

**Group N Missing Median 25% 75%**

SA 5 0 100.000 100.000 100.000

HIB 5 0 125.500 121.050 145.300

IBA 5 0 113.900 101.900 127.950

*H* = 8.732 with 2 degrees of freedom (*P* = 0.013)

The differences in the median values among the treatment groups are greater than would be expected by chance; **there is a statistically significant difference (*P* = 0.013)**

To isolate the group or groups that differ from the others, use a multiple comparison procedure.

All Pairwise Multiple Comparison Procedures (Tukey’s Test):

**Comparison Diff of Ranks *q* *P* *P*<0.050**

**HIB vs SA** 41.000 4.100 0.010 **Yes**

HIB vs IBA 22.000 2.200 0.265 No

IBA vs SA 19.000 1.900 0.371 No

Note: The multiple comparisons on ranks do not include an adjustment for ties.

**Predominant synthesis of giant myofibrillar proteins in striated muscles of the long-tailed ground squirrel *Urocitellus undulatus* during interbout arousal** *Svetlana Popova1, Anna Ulanova1, Yulia Gritsyna1, Nikolay Salmov1, Vadim Rogachevsky2, Gulnara Mikhailova1, Alexander Bobylev1, Liya Bobyleva1, Yana Yutskevich3, Oleg Morenkov4, Nadezda Zakharova5 & Ivan Vikhlyantsev1,**

1Laboratory of the Structure and Functions of Muscle Proteins, Institute of Theoretical and Experimental Biophysics, Russian Academy of Sciences, Pushchino, Moscow Region, 142290, Russia; 2Laboratory of Signal Perception Mechanisms, Institute of Cell Biophysics, FRC PSCBR, Russian Academy of Sciences, Pushchino, Moscow Region, 142290, Russia; 3Kuban State University, Krasnodar, Krasnodar Krai, 350040, Russia; 4Laboratory of Cell Culture and Tissue Engineering, Institute of Cell Biophysics, FRC PSCBR, Russian Academy of Sciences, Pushchino, Moscow Region, 142290, Russia; 5Laboratory of Natural and Artificial Hypobiosis Mechanisms, Institute of Cell Biophysics, FRC PSCBR, Russian Academy of Sciences, Pushchino, Moscow Region, 142290, Russia

**Supplementary Table S25. Rate of total protein synthesis in the heart of ground squirrels, %**

**(PVDF membranes).**

| **Summer activity (SA), *n*=5**  Total protein level puromycin /  Total protein level, % | **Interbout arousal (IBA), *n*=5**  Total protein level puromycin /  Total protein level, % |
| --- | --- |
| 100.0 | 20.0 |
| 100.0 | 20.4 |
| 100.0 | 22.6 |
| 100.0 | 32.1 |
| 100.0 | 40.7 |
| **100±9.7%** | **27.2±8.0%**** |

**↓ 3.67**

The levels of proteins synthesised *in vivo* were identified relative to the total protein level. The total protein level in the samples was measured by the Bradford method according to the manufacturer's recommended protocol (“Sileks”, Russia). Bovine serum albumin was used as a standard.

Total protein level puromycin/Total protein level for the SA group was taken as 100% (control group). For each individual experiment, the average content of 2–3 control tracks in the gel (from the control SA group) present on the blot membrane was taken into consideration. The data were analyzed using the nonparametric Mann–Whitney U criterion. **** *p*<0.01.**

**Supplementary Table S26. Rate of total protein synthesis in m. longissimus dorsi of ground squirrels (PVDF membranes).**

| **Summer activity (SA), *n*=5**  Total protein level puromycin /  Total protein level, % | **Interbout arousal (IBA), *n*=5**  Total protein level puromycin /  Total protein level, % |
| --- | --- |
| 100.0 | 27.0 |
| 100.0 | 30.7 |
| 100.0 | 31.1 |
| 100.0 | 37.3 |
| 100.0 | 43.0 |
| **100.0±18.3%** | **33.8±5.6%**** |

**↓ 2.96**

The levels of proteins synthesised in vivo were identified relative to the total protein level. The total protein level in the samples was measured by the Bradford method according to the manufacturer's recommended protocol (“Sileks”, Russia). Bovine serum albumin was used as a standard.

Total protein level puromycin/Total protein level for the SA group was taken as 100% (control group). For each individual experiment, the average content of 2–3 control tracks in the gel (from the control SA group) present on the blot membrane were taken into consideration. The data were analyzed using the nonparametric Mann–Whitney U criterion. **** *p*<0.01.**

**Predominant synthesis of giant myofibrillar proteins in striated muscles of the long-tailed ground squirrel *Urocitellus undulatus* during interbout arousal** *Svetlana Popova1, Anna Ulanova1, Yulia Gritsyna1, Nikolay Salmov1, Vadim Rogachevsky2, Gulnara Mikhailova1, Alexander Bobylev1, Liya Bobyleva1, Yana Yutskevich3, Oleg Morenkov4, Nadezda Zakharova5 & Ivan Vikhlyantsev1,**

1Laboratory of the Structure and Functions of Muscle Proteins, Institute of Theoretical and Experimental Biophysics, Russian Academy of Sciences, Pushchino, Moscow Region, 142290, Russia; 2Laboratory of Signal Perception Mechanisms, Institute of Cell Biophysics, FRC PSCBR, Russian Academy of Sciences, Pushchino, Moscow Region, 142290, Russia; 3Kuban State University, Krasnodar, Krasnodar Krai, 350040, Russia; 4Laboratory of Cell Culture and Tissue Engineering, Institute of Cell Biophysics, FRC PSCBR, Russian Academy of Sciences, Pushchino, Moscow Region, 142290, Russia; 5Laboratory of Natural and Artificial Hypobiosis Mechanisms, Institute of Cell Biophysics, FRC PSCBR, Russian Academy of Sciences, Pushchino, Moscow Region, 142290, Russia

**Supplementary Table S27. Rate of titin synthesis in the heart of ground squirrels, %**

**(nitrocellulose membranes).**

| **Summer activity (SA), *n*=3** | **Interbout arousal (IBA), *n*=3** |
| --- | --- |
| 100.0 | 84.2 |
| 100.0 | 115.8 |
| 100.0 | 101.1 |
| **100.0±11.0%** | **100.4±12.9%** |

The levels of proteins synthesised *in vivo* were identified relative to the level of total protein (stained with Ponceau S). Titinpuromycin /TitinPonceau S content of the SA group was taken as 100% (control group). For each individual experiment, the average content of 2–3 titin spots (from the control SA group) present on the blot membrane was taken into consideration.

**Supplementary Table S28. Rate of titin synthesis in m. longissimus dorsi of ground squirrels, % (nitrocellulose membranes).**

| **Summer activity (SA), *n*=3** | **Interbout arousal (IBA), *n*=3** |
| --- | --- |
| 100.0 | 117.0 |
| 100.0 | 133.9 |
| 100.0 | 64.2 |
| **100.0±27.0%** | **105.0±29.7%** |

The levels of proteins synthesized *in vivo* were identified relative to the level of total protein (stained with Ponceau S). Titinpuromycin /TitinPonceau S content of the SA group was taken as 100% (control group). For each individual experiment, the average content of 2–3 titin spots (from the control SA group) present on the blot membrane was taken into consideration.

**Supplementary Table S29. Rate of nebulin synthesis in m. longissimus dorsi of ground squirrels, % (nitrocellulose membranes).**

| **Summer activity (SA), *n*=3** | **Interbout arousal (IBA), *n*=3** |
| --- | --- |
| 100.0 | 113.8 |
| 100.0 | 198.3 |
| 100.0 | 63.6 |
| **100.0±26.0%** | **125.2±55.6%** |

The levels of proteins synthesized *in vivo* were identified relative to the level of total protein (stained with Ponceau S). Nebulinpuromycin /NebulinPonceau S content of SA group was taken as 100% (control group). For each individual experiment, the average content of 2–3 nebulin spots (from the control SA group) present on the blot membrane was taken into consideration.

**Predominant synthesis of giant myofibrillar proteins in striated muscles of the long-tailed ground squirrel *Urocitellus undulatus* during interbout arousal** *Svetlana Popova1, Anna Ulanova1, Yulia Gritsyna1, Nikolay Salmov1, Vadim Rogachevsky2, Gulnara Mikhailova1, Alexander Bobylev1, Liya Bobyleva1, Yana Yutskevich3, Oleg Morenkov4, Nadezda Zakharova5 & Ivan Vikhlyantsev1,**

1Laboratory of the Structure and Functions of Muscle Proteins, Institute of Theoretical and Experimental Biophysics, Russian Academy of Sciences, Pushchino, Moscow Region, 142290, Russia; 2Laboratory of Signal Perception Mechanisms, Institute of Cell Biophysics, FRC PSCBR, Russian Academy of Sciences, Pushchino, Moscow Region, 142290, Russia; 3Kuban State University, Krasnodar, Krasnodar Krai, 350040, Russia; 4Laboratory of Cell Culture and Tissue Engineering, Institute of Cell Biophysics, FRC PSCBR, Russian Academy of Sciences, Pushchino, Moscow Region, 142290, Russia; 5Laboratory of Natural and Artificial Hypobiosis Mechanisms, Institute of Cell Biophysics, FRC PSCBR, Russian Academy of Sciences, Pushchino, Moscow Region, 142290, Russia


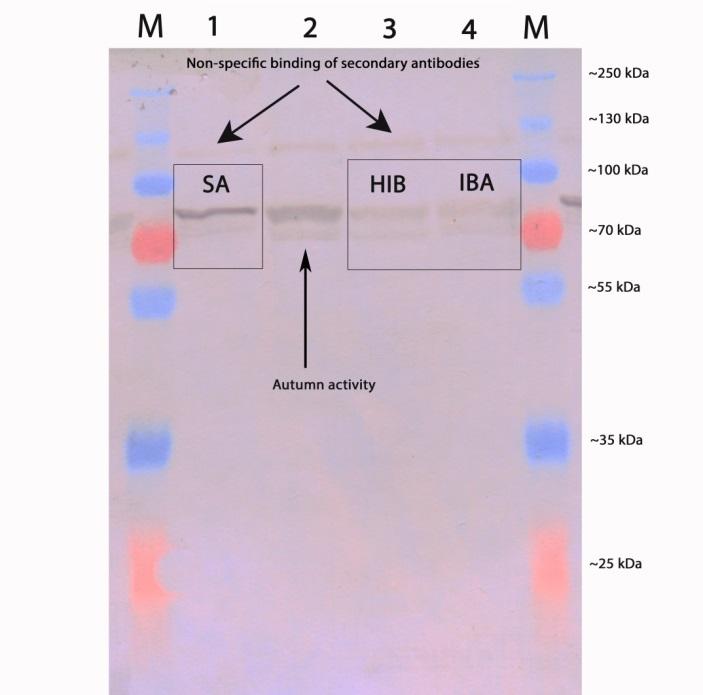


**Supplementary Fig. S3.** Part of the blot for calpain-1 in cardiac muscle.

SA, summer activity; HIB, hibernation; IBA, interbout arousal. The first, third and fourth tracks were selected for Figure 2A in the article. Secondary antibodies conjugated to alkaline phosphatase (goat anti-rabbit Ig, ab6722) were used.

M, molecular weight marker.


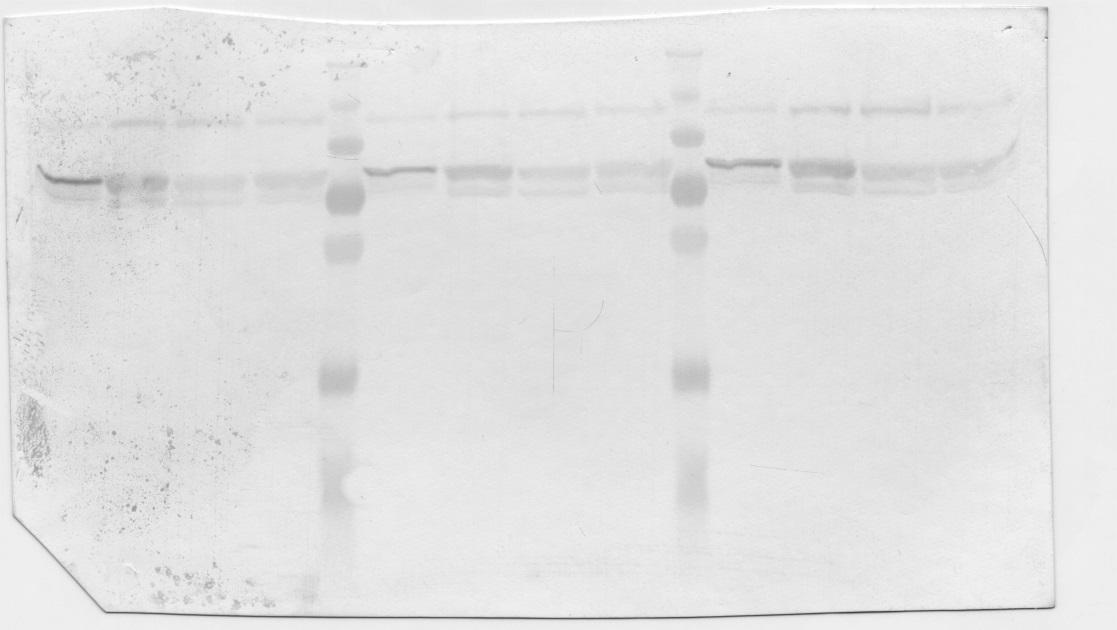


**Supplementary Fig. S4.** Full-length blot for calpain-1 in cardiac muscle. The original image is here:

<https://drive.google.com/open?id=1XljyCN3sfWktjuHRPACab3eQfOc6i2AC>

**Predominant synthesis of giant myofibrillar proteins in striated muscles of the long-tailed ground squirrel *Urocitellus undulatus* during interbout arousal** *Svetlana Popova1, Anna Ulanova1, Yulia Gritsyna1, Nikolay Salmov1, Vadim Rogachevsky2, Gulnara Mikhailova1, Alexander Bobylev1, Liya Bobyleva1, Yana Yutskevich3, Oleg Morenkov4, Nadezda Zakharova5 & Ivan Vikhlyantsev1,**

1Laboratory of the Structure and Functions of Muscle Proteins, Institute of Theoretical and Experimental Biophysics, Russian Academy of Sciences, Pushchino, Moscow Region, 142290, Russia; 2Laboratory of Signal Perception Mechanisms, Institute of Cell Biophysics, FRC PSCBR, Russian Academy of Sciences, Pushchino, Moscow Region, 142290, Russia; 3Kuban State University, Krasnodar, Krasnodar Krai, 350040, Russia; 4Laboratory of Cell Culture and Tissue Engineering, Institute of Cell Biophysics, FRC PSCBR, Russian Academy of Sciences, Pushchino, Moscow Region, 142290, Russia; 5Laboratory of Natural and Artificial Hypobiosis Mechanisms, Institute of Cell Biophysics, FRC PSCBR, Russian Academy of Sciences, Pushchino, Moscow Region, 142290, Russia


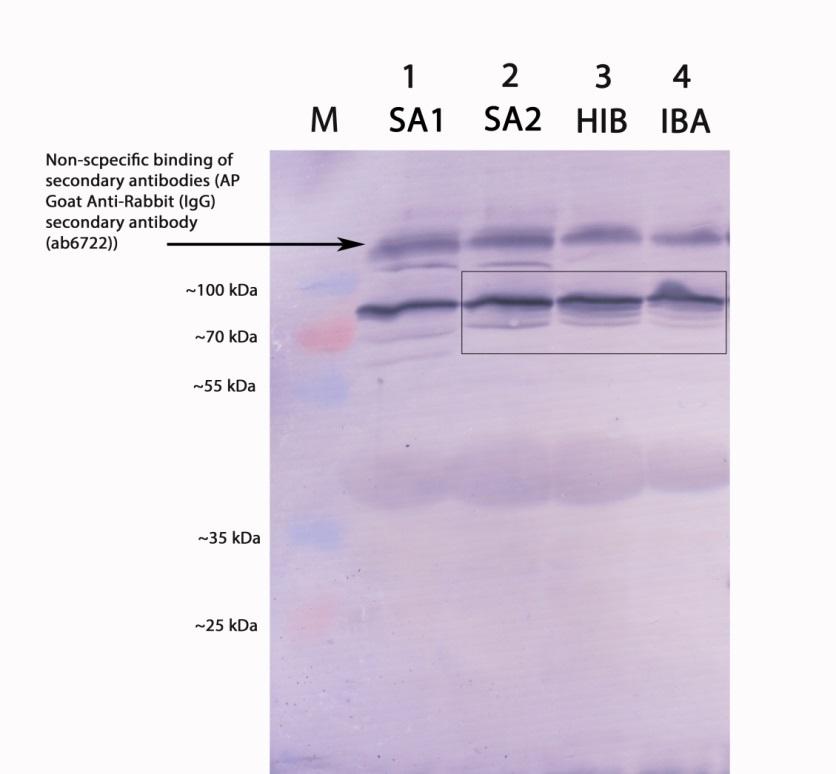


**Supplementary Fig. S5.** Part of the blot for calpain-1 in m. longissimus dorsi.

SA, summer activity; HIB, hibernation; IBA, interbout arousal. The second, third and fourth tracks were selected for Figure 2A in the article. Secondary antibodies conjugated to alkaline phosphatase (goat anti-rabbit Ig, ab6722) were used. M, molecular weight marker.


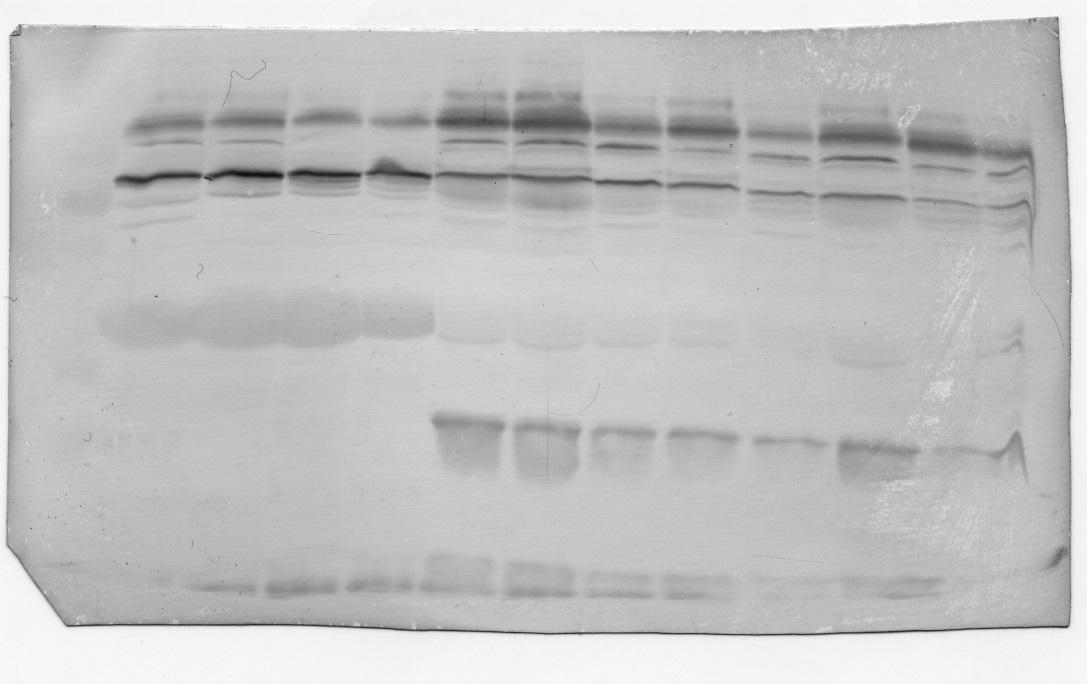


**Supplementary Fig. S6.** Full-length blot for calpain-1 in m. longissimus dorsi.

The original image is here:

<https://drive.google.com/open?id=1XljyCN3sfWktjuHRPACab3eQfOc6i2AC>

**Predominant synthesis of giant myofibrillar proteins in striated muscles of the long-tailed ground squirrel *Urocitellus undulatus* during interbout arousal** *Svetlana Popova1, Anna Ulanova1, Yulia Gritsyna1, Nikolay Salmov1, Vadim Rogachevsky2, Gulnara Mikhailova1, Alexander Bobylev1, Liya Bobyleva1, Yana Yutskevich3, Oleg Morenkov4, Nadezda Zakharova5 & Ivan Vikhlyantsev1,**

1Laboratory of the Structure and Functions of Muscle Proteins, Institute of Theoretical and Experimental Biophysics, Russian Academy of Sciences, Pushchino, Moscow Region, 142290, Russia; 2Laboratory of Signal Perception Mechanisms, Institute of Cell Biophysics, FRC PSCBR, Russian Academy of Sciences, Pushchino, Moscow Region, 142290, Russia; 3Kuban State University, Krasnodar, Krasnodar Krai, 350040, Russia; 4Laboratory of Cell Culture and Tissue Engineering, Institute of Cell Biophysics, FRC PSCBR, Russian Academy of Sciences, Pushchino, Moscow Region, 142290, Russia; 5Laboratory of Natural and Artificial Hypobiosis Mechanisms, Institute of Cell Biophysics, FRC PSCBR, Russian Academy of Sciences, Pushchino, Moscow Region, 142290, Russia


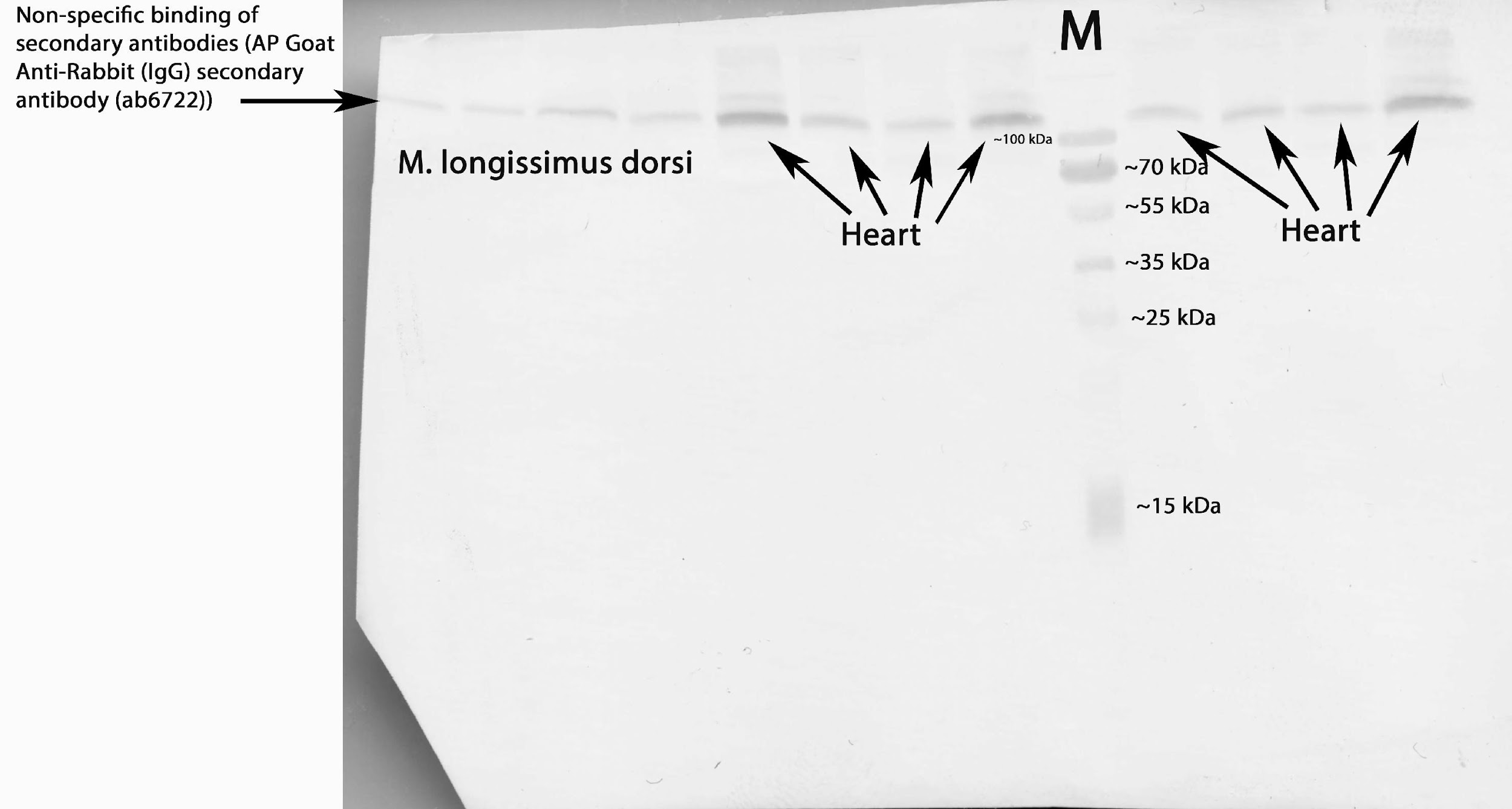


**Supplementary Fig. S7.** Non-specific binding of secondary antibodies (AP goat anti-rabbit (IgG) secondary antibodies ab6722)). M, molecular weight marker. Incubation was conducted with only the secondary antibodies.

**Predominant synthesis of giant myofibrillar proteins in striated muscles of the long-tailed ground squirrel *Urocitellus undulatus* during interbout arousal** *Svetlana Popova1, Anna Ulanova1, Yulia Gritsyna1, Nikolay Salmov1, Vadim Rogachevsky2, Gulnara Mikhailova1, Alexander Bobylev1, Liya Bobyleva1, Yana Yutskevich3, Oleg Morenkov4, Nadezda Zakharova5 & Ivan Vikhlyantsev1,**

1Laboratory of the Structure and Functions of Muscle Proteins, Institute of Theoretical and Experimental Biophysics, Russian Academy of Sciences, Pushchino, Moscow Region, 142290, Russia; 2Laboratory of Signal Perception Mechanisms, Institute of Cell Biophysics, FRC PSCBR, Russian Academy of Sciences, Pushchino, Moscow Region, 142290, Russia; 3Kuban State University, Krasnodar, Krasnodar Krai, 350040, Russia; 4Laboratory of Cell Culture and Tissue Engineering, Institute of Cell Biophysics, FRC PSCBR, Russian Academy of Sciences, Pushchino, Moscow Region, 142290, Russia; 5Laboratory of Natural and Artificial Hypobiosis Mechanisms, Institute of Cell Biophysics, FRC PSCBR, Russian Academy of Sciences, Pushchino, Moscow Region, 142290, Russia


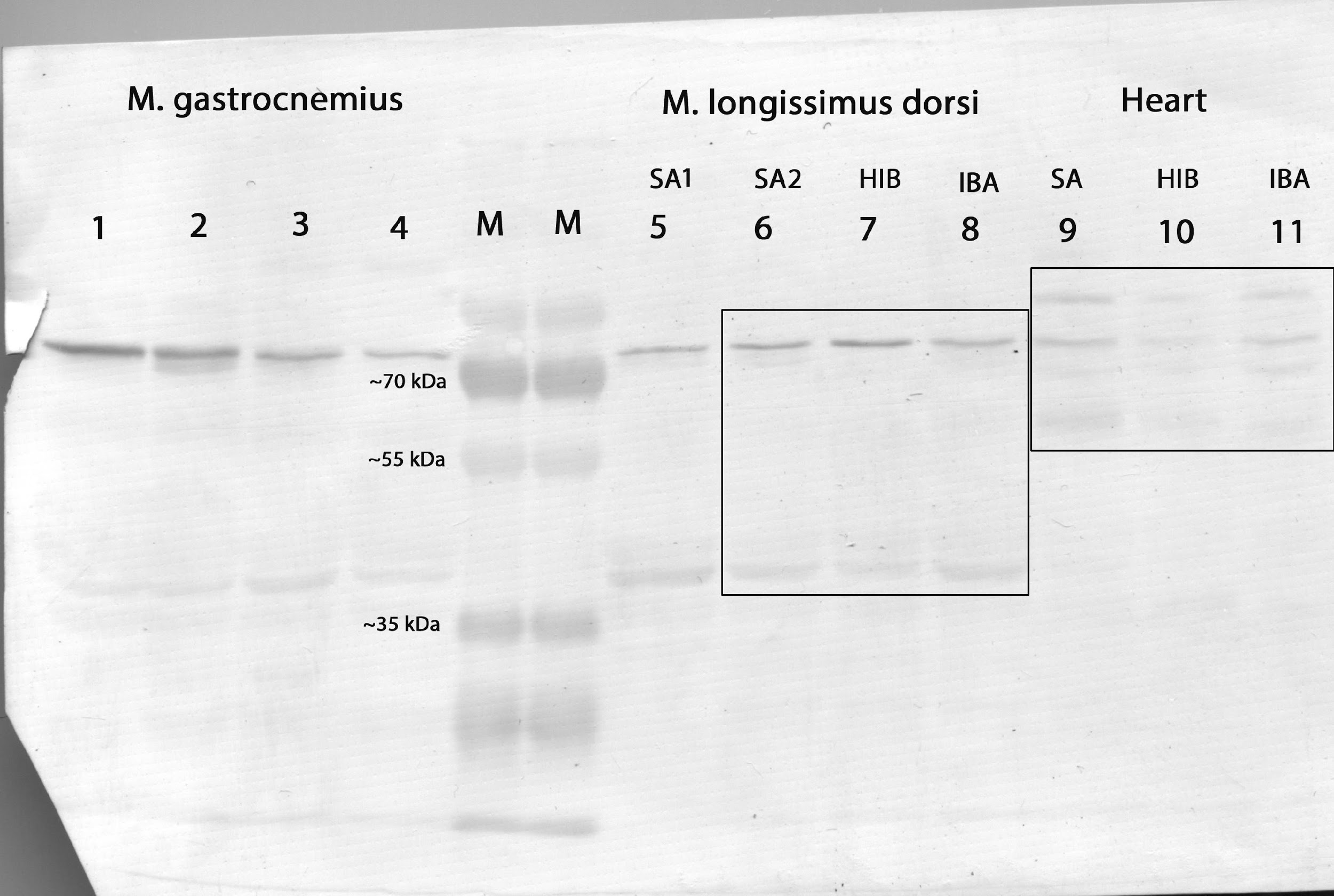


**Supplementary Fig. S8.** Full-length blot for calpastatin in m. longissimus dorsi (tracks 6–8) and in cardiac muscle (tracks 9–11). SA, summer activity; HIB, hibernation; IBA, interbout arousal. Lanes 6 to 11 were taken for Figure 2A in the article. M, molecular weight marker. The original image is here:

<https://drive.google.com/open?id=1XljyCN3sfWktjuHRPACab3eQfOc6i2AC>

**Predominant synthesis of giant myofibrillar proteins in striated muscles of the long-tailed ground squirrel *Urocitellus undulatus* during interbout arousal** *Svetlana Popova1, Anna Ulanova1, Yulia Gritsyna1, Nikolay Salmov1, Vadim Rogachevsky2, Gulnara Mikhailova1, Alexander Bobylev1, Liya Bobyleva1, Yana Yutskevich3, Oleg Morenkov4, Nadezda Zakharova5 & Ivan Vikhlyantsev1,**

1Laboratory of the Structure and Functions of Muscle Proteins, Institute of Theoretical and Experimental Biophysics, Russian Academy of Sciences, Pushchino, Moscow Region, 142290, Russia; 2Laboratory of Signal Perception Mechanisms, Institute of Cell Biophysics, FRC PSCBR, Russian Academy of Sciences, Pushchino, Moscow Region, 142290, Russia; 3Kuban State University, Krasnodar, Krasnodar Krai, 350040, Russia; 4Laboratory of Cell Culture and Tissue Engineering, Institute of Cell Biophysics, FRC PSCBR, Russian Academy of Sciences, Pushchino, Moscow Region, 142290, Russia; 5Laboratory of Natural and Artificial Hypobiosis Mechanisms, Institute of Cell Biophysics, FRC PSCBR, Russian Academy of Sciences, Pushchino, Moscow Region, 142290, Russia


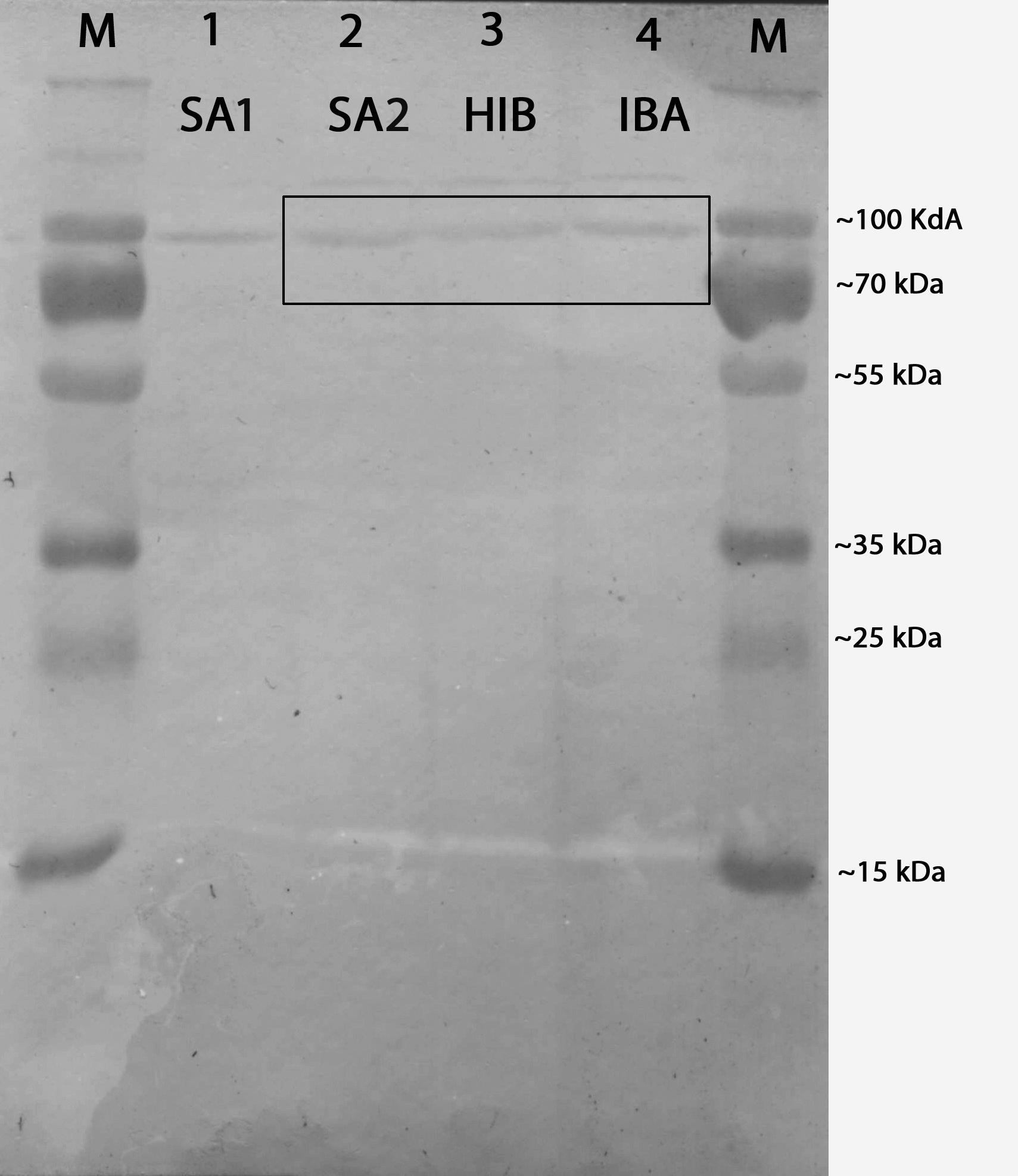


**Supplementary Fig. S9**. Part of the blot for Hsp90 in cardiac muscle. SA, summer activity; HIB, hibernation; IBA, interbout arousal. Lanes 2 to 4 were taken for Figure 2A in the article. M, molecular weight marker.


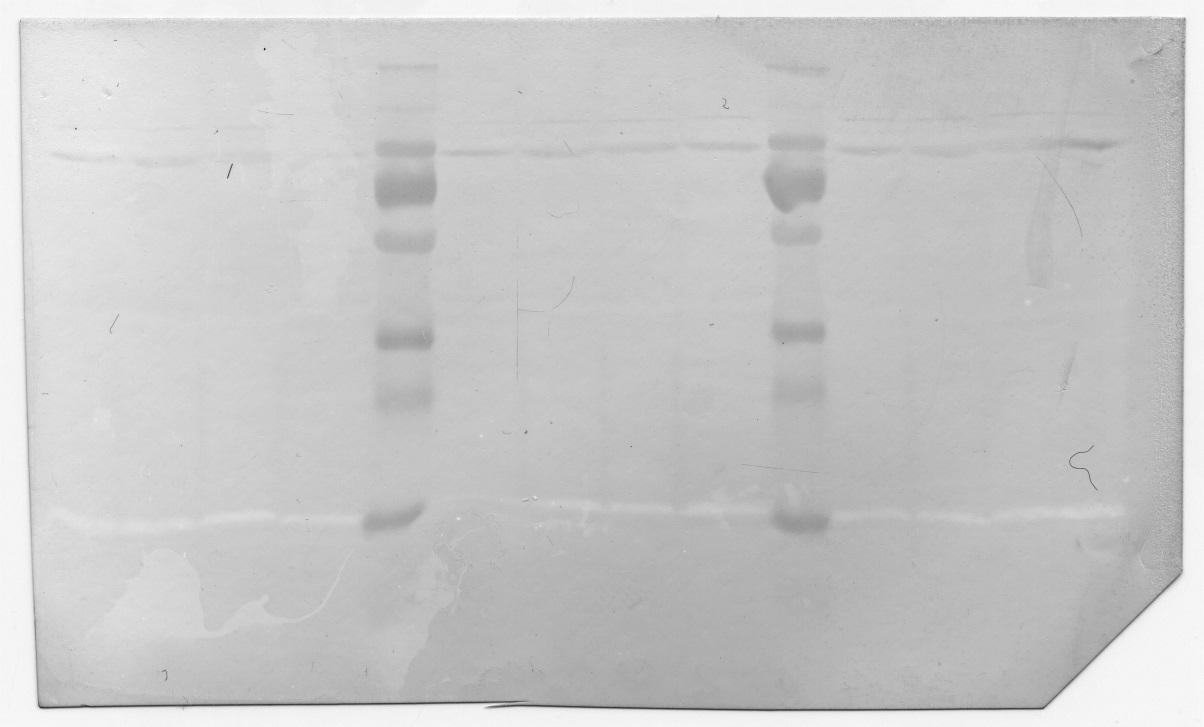


**Supplementary Fig. S10**. Full-length blot for Hsp90 in cardiac muscle.

The original image is here:

<https://drive.google.com/open?id=1XljyCN3sfWktjuHRPACab3eQfOc6i2AC>

**Predominant synthesis of giant myofibrillar proteins in striated muscles of the long-tailed ground squirrel *Urocitellus undulatus* during interbout arousal** *Svetlana Popova1, Anna Ulanova1, Yulia Gritsyna1, Nikolay Salmov1, Vadim Rogachevsky2, Gulnara Mikhailova1, Alexander Bobylev1, Liya Bobyleva1, Yana Yutskevich3, Oleg Morenkov4, Nadezda Zakharova5 & Ivan Vikhlyantsev1,**

1Laboratory of the Structure and Functions of Muscle Proteins, Institute of Theoretical and Experimental Biophysics, Russian Academy of Sciences, Pushchino, Moscow Region, 142290, Russia; 2Laboratory of Signal Perception Mechanisms, Institute of Cell Biophysics, FRC PSCBR, Russian Academy of Sciences, Pushchino, Moscow Region, 142290, Russia; 3Kuban State University, Krasnodar, Krasnodar Krai, 350040, Russia; 4Laboratory of Cell Culture and Tissue Engineering, Institute of Cell Biophysics, FRC PSCBR, Russian Academy of Sciences, Pushchino, Moscow Region, 142290, Russia; 5Laboratory of Natural and Artificial Hypobiosis Mechanisms, Institute of Cell Biophysics, FRC PSCBR, Russian Academy of Sciences, Pushchino, Moscow Region, 142290, Russia


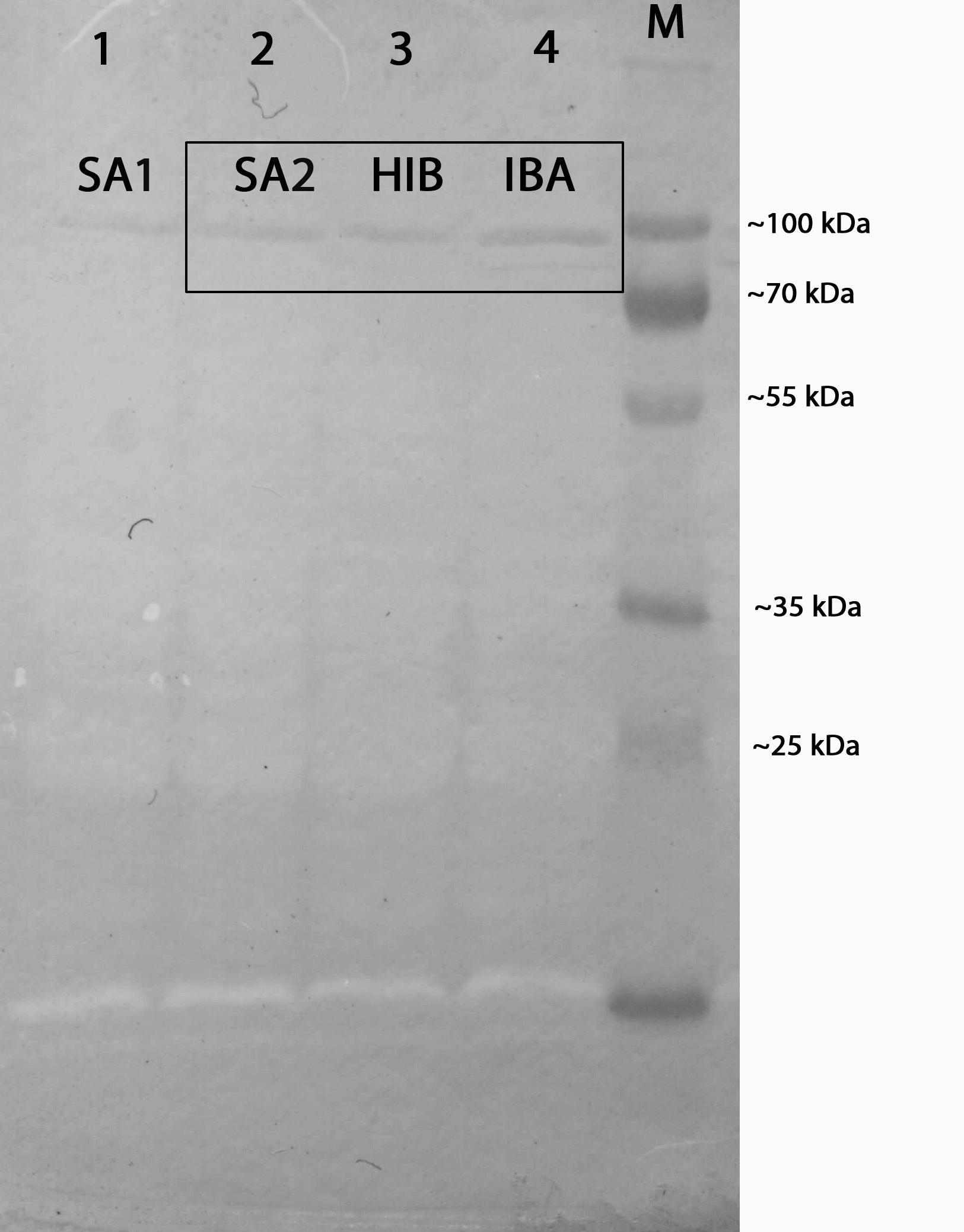


**Supplementary Fig. S11**. Part of the blot for Hsp90 in m. longissimus dorsi. SA, summer activity; HIB, hibernation; IBA, interbout arousal. Lanes 2 to 4 were taken for Figure 2A in the article. M, molecular weight marker.


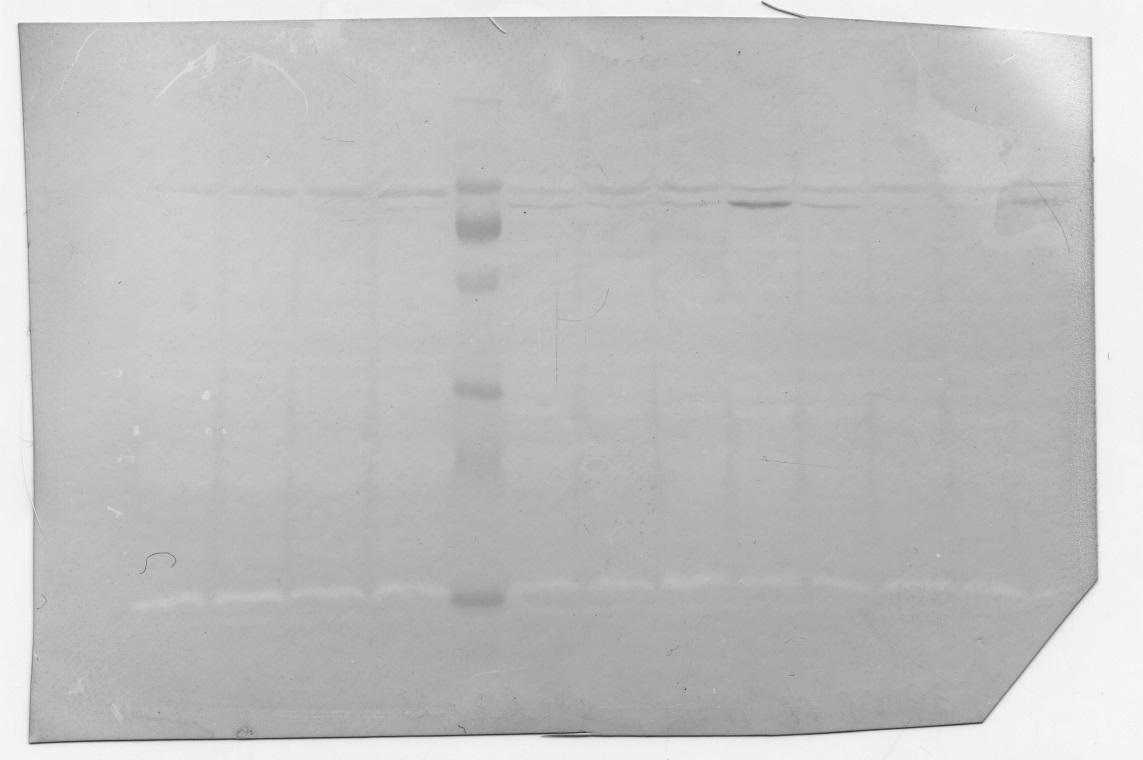


**Supplementary Fig. S12**. Full-length blot for Hsp90 in m. longissimus dorsi.

The original image is here:

<https://drive.google.com/open?id=1XljyCN3sfWktjuHRPACab3eQfOc6i2AC>

**Predominant synthesis of giant myofibrillar proteins in striated muscles of the long-tailed ground squirrel *Urocitellus undulatus* during interbout arousal** *Svetlana Popova1, Anna Ulanova1, Yulia Gritsyna1, Nikolay Salmov1, Vadim Rogachevsky2, Gulnara Mikhailova1, Alexander Bobylev1, Liya Bobyleva1, Yana Yutskevich3, Oleg Morenkov4, Nadezda Zakharova5 & Ivan Vikhlyantsev1,**

1Laboratory of the Structure and Functions of Muscle Proteins, Institute of Theoretical and Experimental Biophysics, Russian Academy of Sciences, Pushchino, Moscow Region, 142290, Russia; 2Laboratory of Signal Perception Mechanisms, Institute of Cell Biophysics, FRC PSCBR, Russian Academy of Sciences, Pushchino, Moscow Region, 142290, Russia; 3Kuban State University, Krasnodar, Krasnodar Krai, 350040, Russia; 4Laboratory of Cell Culture and Tissue Engineering, Institute of Cell Biophysics, FRC PSCBR, Russian Academy of Sciences, Pushchino, Moscow Region, 142290, Russia; 5Laboratory of Natural and Artificial Hypobiosis Mechanisms, Institute of Cell Biophysics, FRC PSCBR, Russian Academy of Sciences, Pushchino, Moscow Region, 142290, Russia


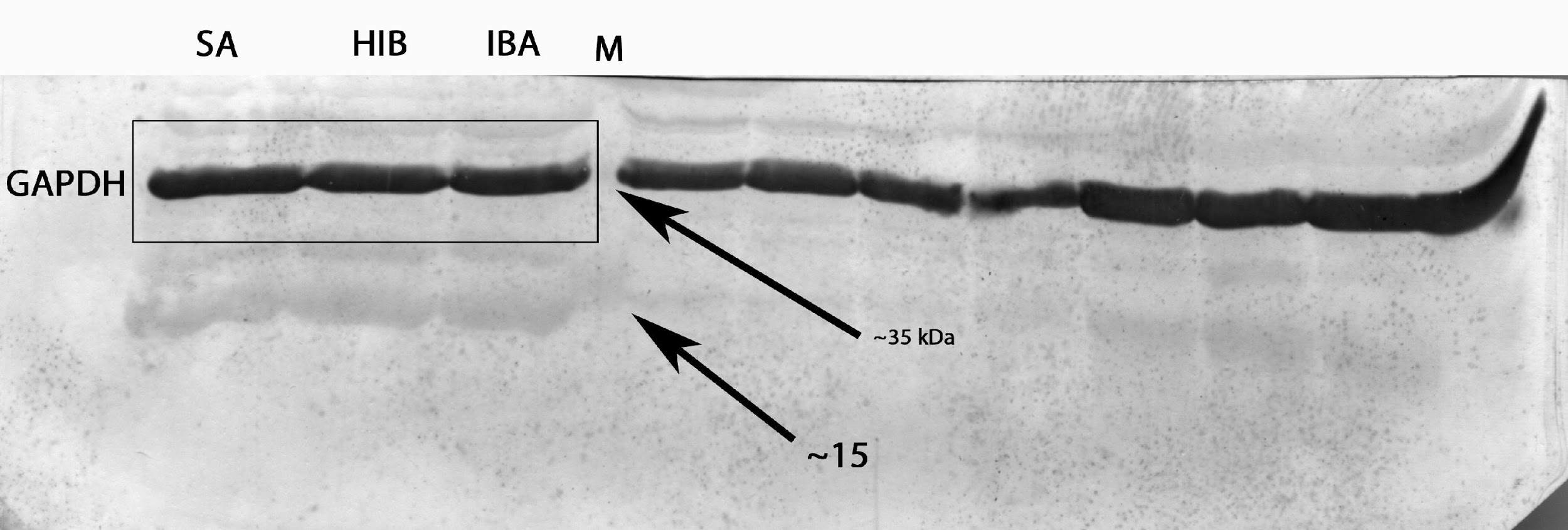
A


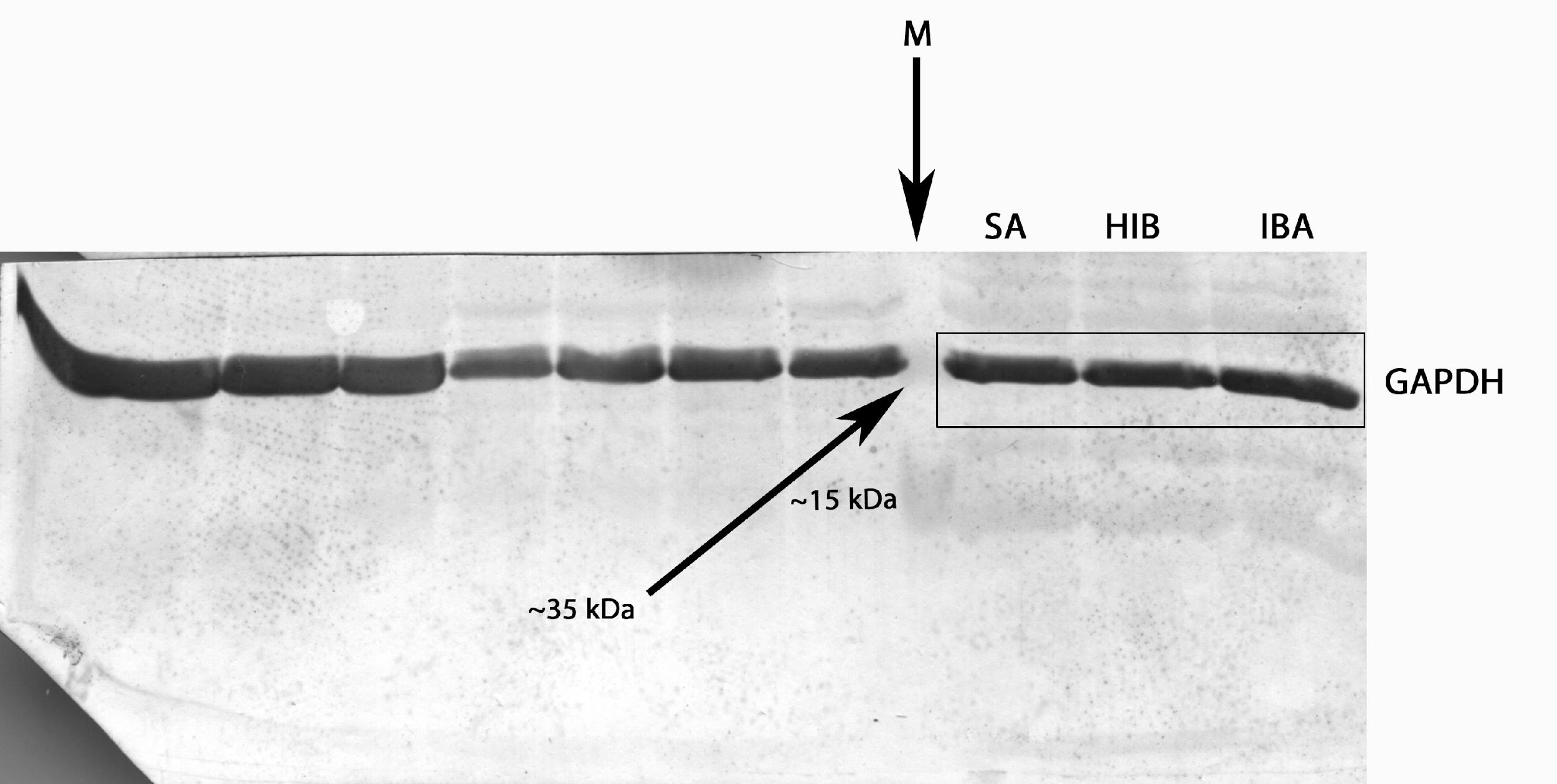
B

**Supplementary Fig. S13**. Full-length blots for GAPDH in cardiac muscle (A) and m. longissimus dorsi (B).

SA, summer activity; HIB, hibernation; IBA, interbout arousal. The boxed areas were taken for Figure 2A in the article. M, molecular weight marker.

The original image is here:

<https://drive.google.com/open?id=1XljyCN3sfWktjuHRPACab3eQfOc6i2AC>

**Predominant synthesis of giant myofibrillar proteins in striated muscles of the long-tailed ground squirrel *Urocitellus undulatus* during interbout arousal** *Svetlana Popova1, Anna Ulanova1, Yulia Gritsyna1, Nikolay Salmov1, Vadim Rogachevsky2, Gulnara Mikhailova1, Alexander Bobylev1, Liya Bobyleva1, Yana Yutskevich3, Oleg Morenkov4, Nadezda Zakharova5 & Ivan Vikhlyantsev1,**

1Laboratory of the Structure and Functions of Muscle Proteins, Institute of Theoretical and Experimental Biophysics, Russian Academy of Sciences, Pushchino, Moscow Region, 142290, Russia; 2Laboratory of Signal Perception Mechanisms, Institute of Cell Biophysics, FRC PSCBR, Russian Academy of Sciences, Pushchino, Moscow Region, 142290, Russia; 3Kuban State University, Krasnodar, Krasnodar Krai, 350040, Russia; 4Laboratory of Cell Culture and Tissue Engineering, Institute of Cell Biophysics, FRC PSCBR, Russian Academy of Sciences, Pushchino, Moscow Region, 142290, Russia; 5Laboratory of Natural and Artificial Hypobiosis Mechanisms, Institute of Cell Biophysics, FRC PSCBR, Russian Academy of Sciences, Pushchino, Moscow Region, 142290, Russia


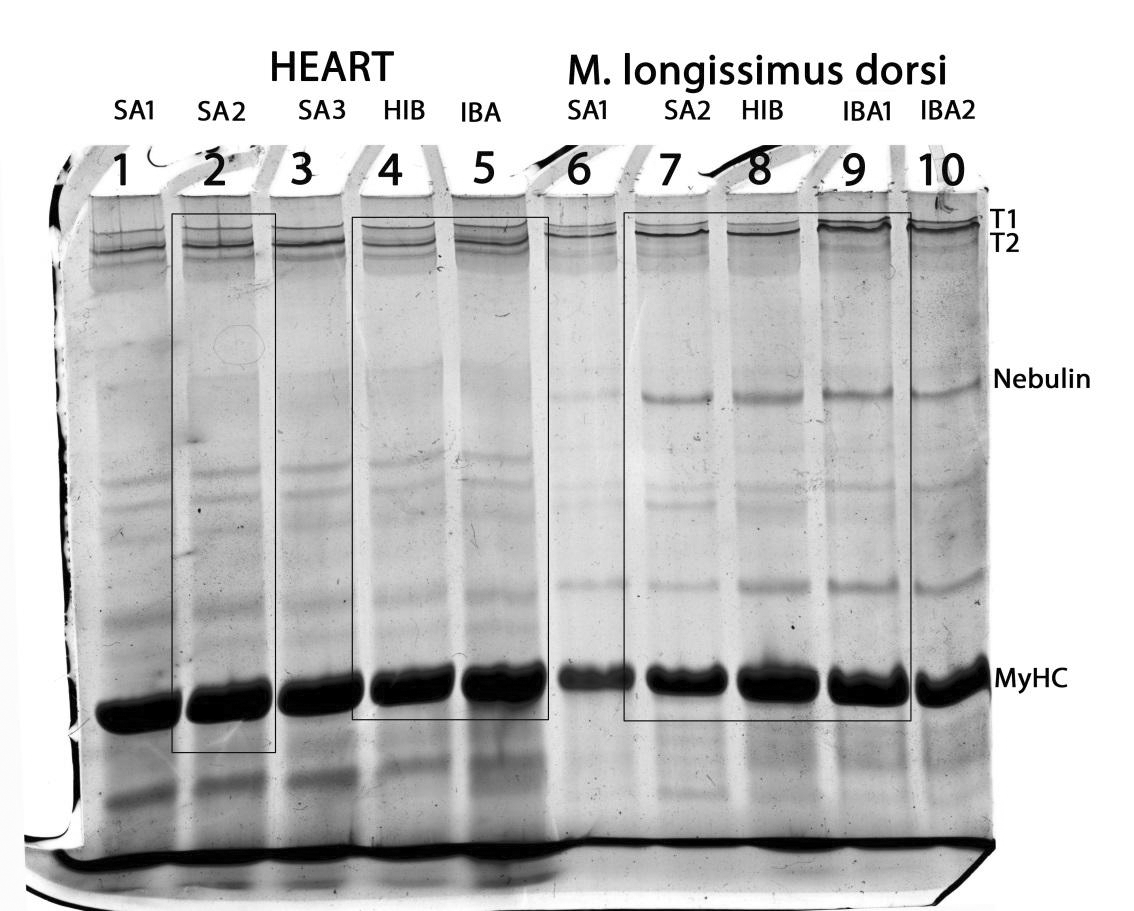


**Supplementary Fig. S14**. SDS-PAGE analysis of titin in the heart (left) and titin and nebulin in the m. longissimus dorsi (LD, right). Full-length gel.

SA, summer activity; HIB, hibernation; IBA, interbout arousal. Lanes 2, 4, 5, 7–9 were selected for Figure 3A in the article. MyHC, myosin heavy chains.

The original image is here:

<https://drive.google.com/open?id=1XljyCN3sfWktjuHRPACab3eQfOc6i2AC>

**Predominant synthesis of giant myofibrillar proteins in striated muscles of the long-tailed ground squirrel *Urocitellus undulatus* during interbout arousal** *Svetlana Popova1, Anna Ulanova1, Yulia Gritsyna1, Nikolay Salmov1, Vadim Rogachevsky2, Gulnara Mikhailova1, Alexander Bobylev1, Liya Bobyleva1, Yana Yutskevich3, Oleg Morenkov4, Nadezda Zakharova5 & Ivan Vikhlyantsev1,**

1Laboratory of the Structure and Functions of Muscle Proteins, Institute of Theoretical and Experimental Biophysics, Russian Academy of Sciences, Pushchino, Moscow Region, 142290, Russia; 2Laboratory of Signal Perception Mechanisms, Institute of Cell Biophysics, FRC PSCBR, Russian Academy of Sciences, Pushchino, Moscow Region, 142290, Russia; 3Kuban State University, Krasnodar, Krasnodar Krai, 350040, Russia; 4Laboratory of Cell Culture and Tissue Engineering, Institute of Cell Biophysics, FRC PSCBR, Russian Academy of Sciences, Pushchino, Moscow Region, 142290, Russia; 5Laboratory of Natural and Artificial Hypobiosis Mechanisms, Institute of Cell Biophysics, FRC PSCBR, Russian Academy of Sciences, Pushchino, Moscow Region, 142290, Russia


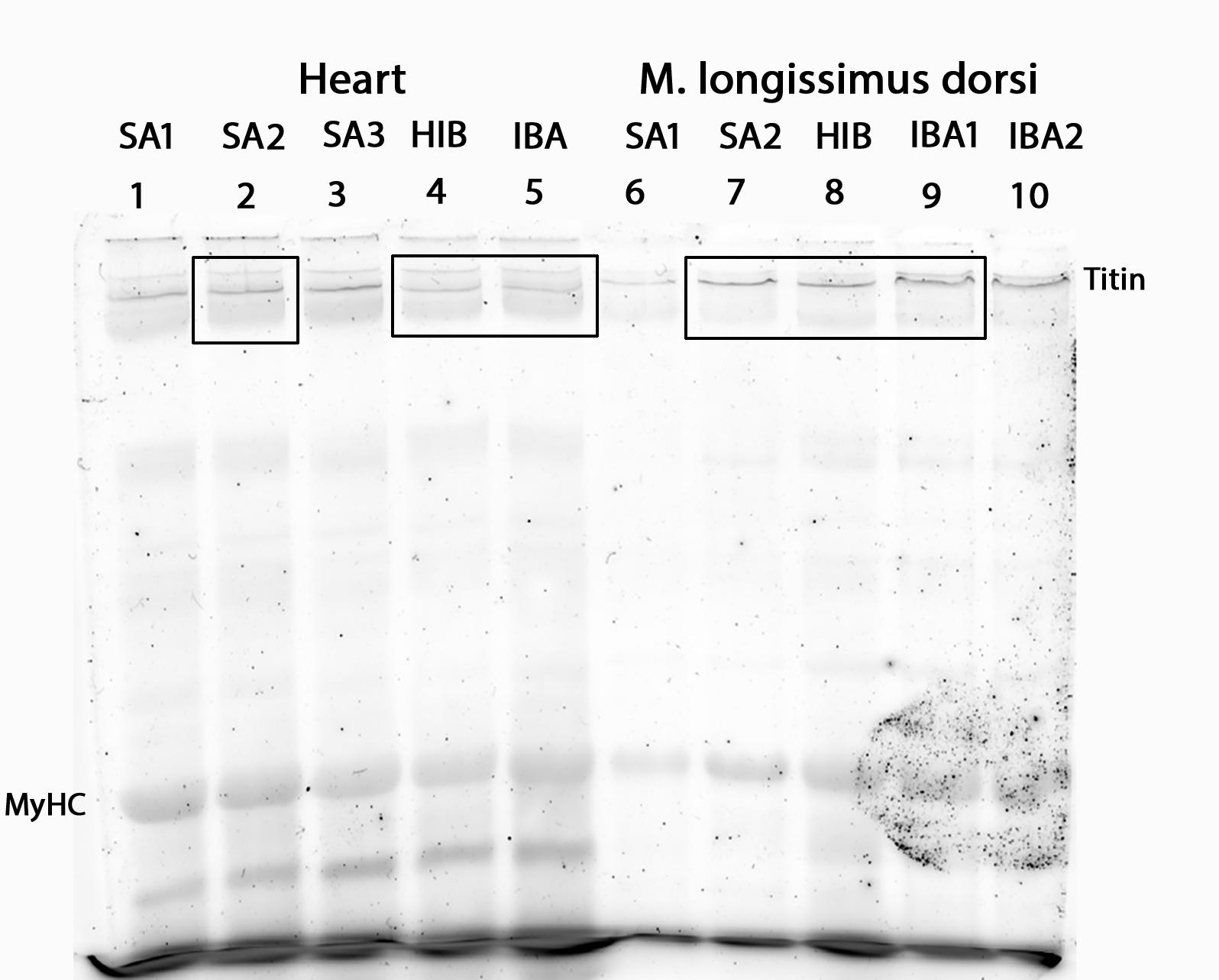


**Supplementary Fig. S15**. Full-length gel with native level of titin phosphorylation (Pro-Q Diamond fluorescent dye, ThermoFisher Scientific).

SA, summer activity; HIB, hibernation; IBA, interbout arousal. Selected areas (lanes 2, 4, 5, 7–9) were taken for Figure 4A in the article. MyHC, myosin heavy chains.

The original image is here:

<https://drive.google.com/open?id=1XljyCN3sfWktjuHRPACab3eQfOc6i2AC>

**Predominant synthesis of giant myofibrillar proteins in striated muscles of the long-tailed ground squirrel *Urocitellus undulatus* during interbout arousal** *Svetlana Popova1, Anna Ulanova1, Yulia Gritsyna1, Nikolay Salmov1, Vadim Rogachevsky2, Gulnara Mikhailova1, Alexander Bobylev1, Liya Bobyleva1, Yana Yutskevich3, Oleg Morenkov4, Nadezda Zakharova5 & Ivan Vikhlyantsev1,**

1Laboratory of the Structure and Functions of Muscle Proteins, Institute of Theoretical and Experimental Biophysics, Russian Academy of Sciences, Pushchino, Moscow Region, 142290, Russia; 2Laboratory of Signal Perception Mechanisms, Institute of Cell Biophysics, FRC PSCBR, Russian Academy of Sciences, Pushchino, Moscow Region, 142290, Russia; 3Kuban State University, Krasnodar, Krasnodar Krai, 350040, Russia; 4Laboratory of Cell Culture and Tissue Engineering, Institute of Cell Biophysics, FRC PSCBR, Russian Academy of Sciences, Pushchino, Moscow Region, 142290, Russia; 5Laboratory of Natural and Artificial Hypobiosis Mechanisms, Institute of Cell Biophysics, FRC PSCBR, Russian Academy of Sciences, Pushchino, Moscow Region, 142290, Russia


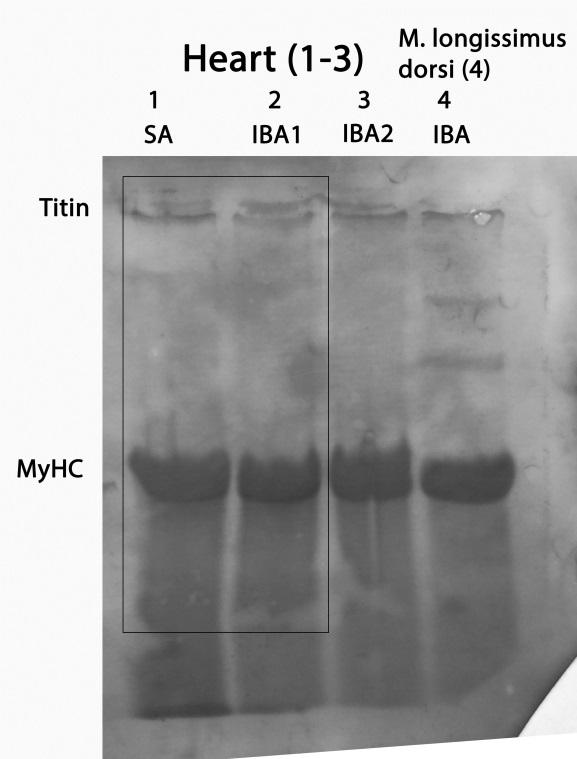


**Supplementary Fig. S16**. Part of the Ponceau S stained membrane (cardiac muscle). Lanes 1 and 2 were taken for Figure 7B in the article. SA, summer activity; IBA, interbout arousal. MyHC, myosin heavy chains.


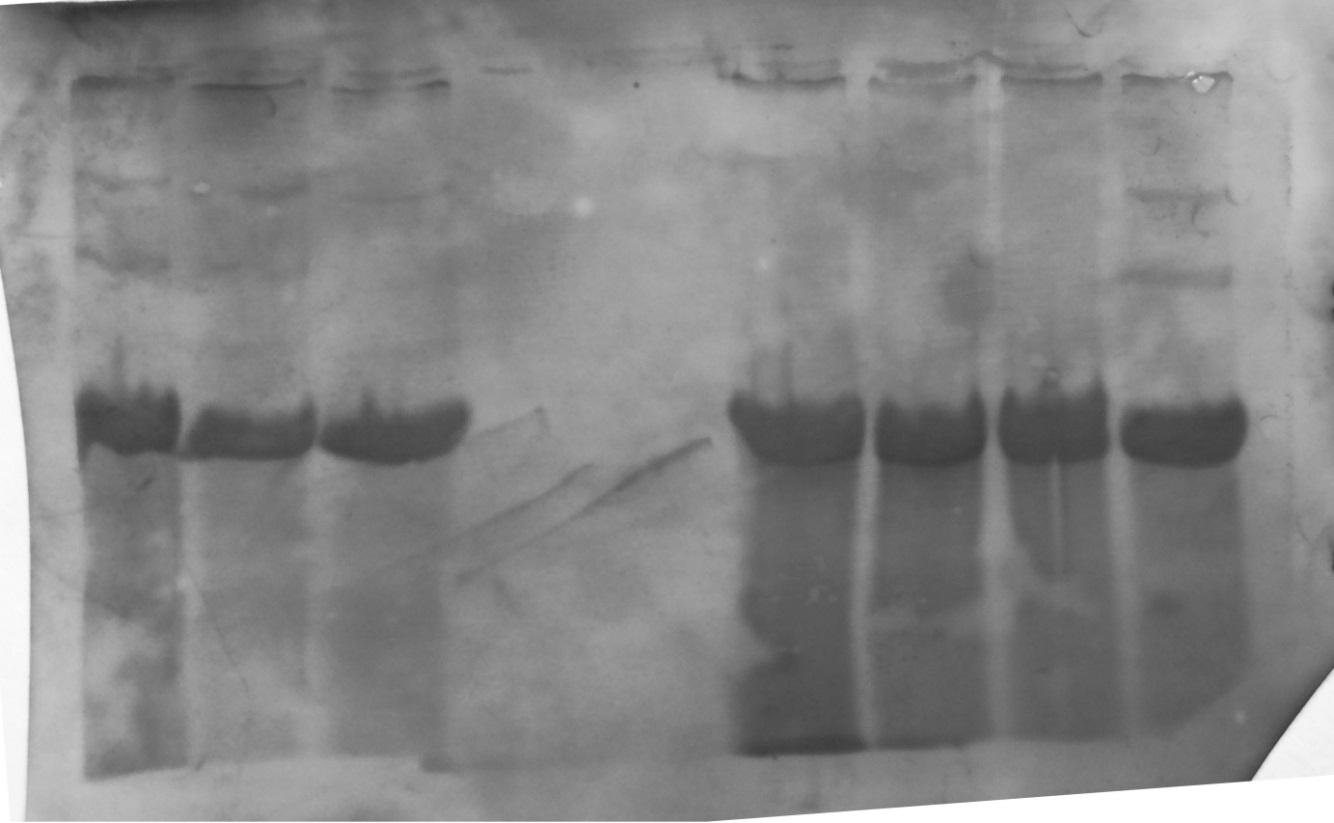


**Supplementary Fig. S17**. Full-length Ponceau S stained membrane (cardiac muscle).

The original image is here:

<https://drive.google.com/open?id=1XljyCN3sfWktjuHRPACab3eQfOc6i2AC>

**Predominant synthesis of giant myofibrillar proteins in striated muscles of the long-tailed ground squirrel *Urocitellus undulatus* during interbout arousal** *Svetlana Popova1, Anna Ulanova1, Yulia Gritsyna1, Nikolay Salmov1, Vadim Rogachevsky2, Gulnara Mikhailova1, Alexander Bobylev1, Liya Bobyleva1, Yana Yutskevich3, Oleg Morenkov4, Nadezda Zakharova5 & Ivan Vikhlyantsev1,**

1Laboratory of the Structure and Functions of Muscle Proteins, Institute of Theoretical and Experimental Biophysics, Russian Academy of Sciences, Pushchino, Moscow Region, 142290, Russia; 2Laboratory of Signal Perception Mechanisms, Institute of Cell Biophysics, FRC PSCBR, Russian Academy of Sciences, Pushchino, Moscow Region, 142290, Russia; 3Kuban State University, Krasnodar, Krasnodar Krai, 350040, Russia; 4Laboratory of Cell Culture and Tissue Engineering, Institute of Cell Biophysics, FRC PSCBR, Russian Academy of Sciences, Pushchino, Moscow Region, 142290, Russia; 5Laboratory of Natural and Artificial Hypobiosis Mechanisms, Institute of Cell Biophysics, FRC PSCBR, Russian Academy of Sciences, Pushchino, Moscow Region, 142290, Russia


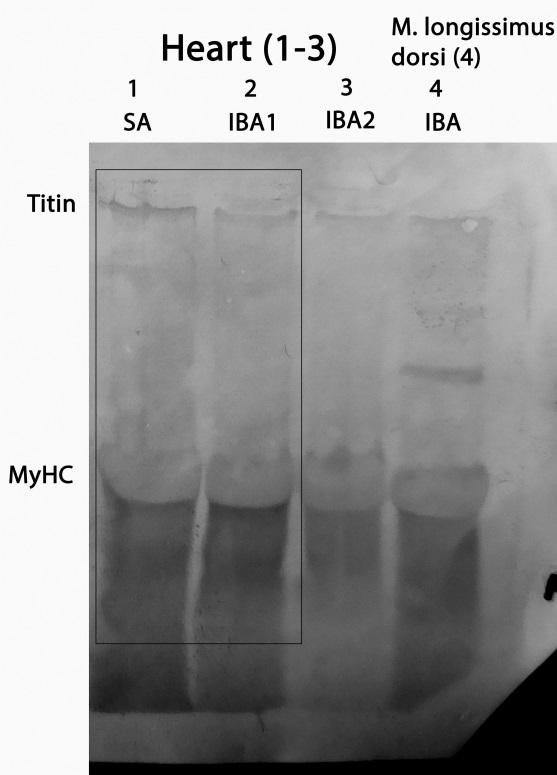


**Supplementary Fig. S18**. Part of the puromycin stained membrane (cardiac muscle). Lanes 1 and 2 were taken for Figure 7B in the article. SA, summer activity; IBA, interbout arousal. MyHC, myosin heavy chains.


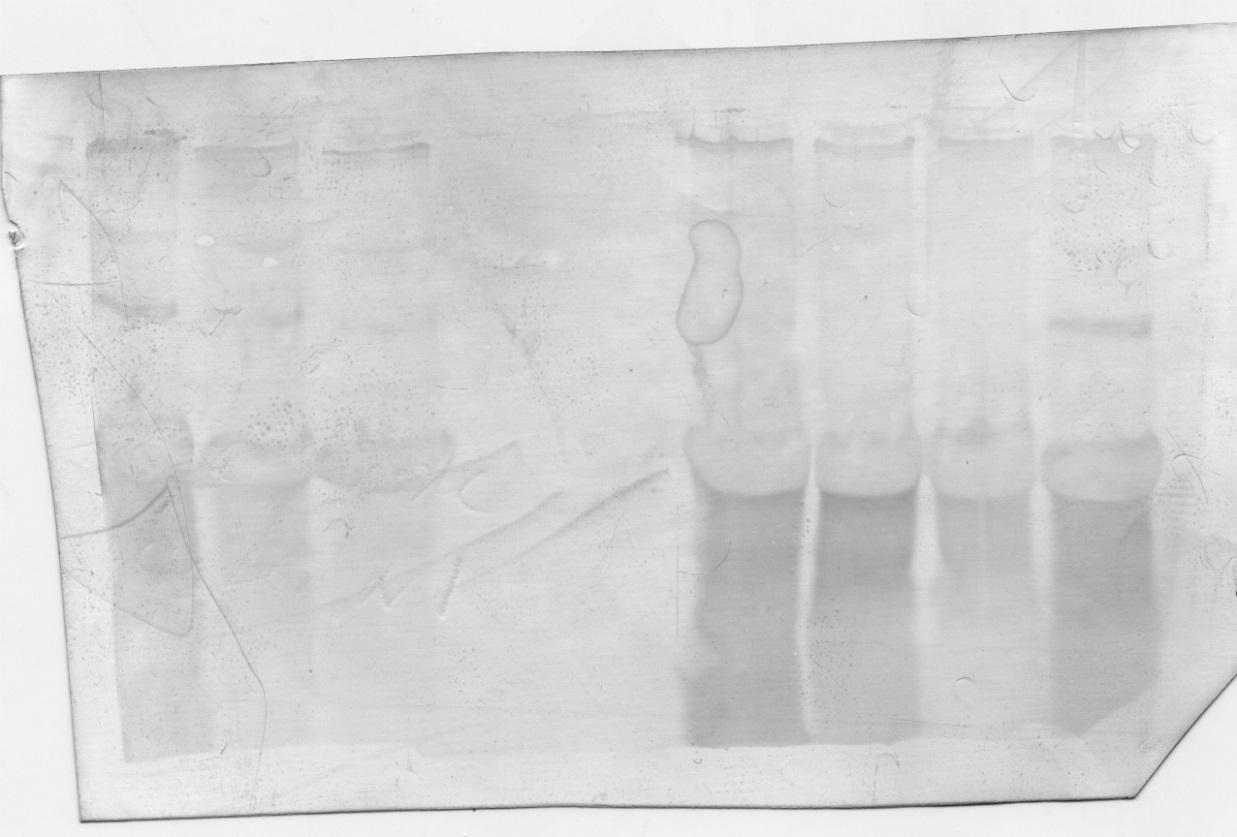


**Supplementary Fig. S19**. Full-length puromycin stained membrane (cardiac muscle).

The original image is here:

<https://drive.google.com/open?id=1XljyCN3sfWktjuHRPACab3eQfOc6i2AC>

**Predominant synthesis of giant myofibrillar proteins in striated muscles of the long-tailed ground squirrel *Urocitellus undulatus* during interbout arousal** *Svetlana Popova1, Anna Ulanova1, Yulia Gritsyna1, Nikolay Salmov1, Vadim Rogachevsky2, Gulnara Mikhailova1, Alexander Bobylev1, Liya Bobyleva1, Yana Yutskevich3, Oleg Morenkov4, Nadezda Zakharova5 & Ivan Vikhlyantsev1,**

1Laboratory of the Structure and Functions of Muscle Proteins, Institute of Theoretical and Experimental Biophysics, Russian Academy of Sciences, Pushchino, Moscow Region, 142290, Russia; 2Laboratory of Signal Perception Mechanisms, Institute of Cell Biophysics, FRC PSCBR, Russian Academy of Sciences, Pushchino, Moscow Region, 142290, Russia; 3Kuban State University, Krasnodar, Krasnodar Krai, 350040, Russia; 4Laboratory of Cell Culture and Tissue Engineering, Institute of Cell Biophysics, FRC PSCBR, Russian Academy of Sciences, Pushchino, Moscow Region, 142290, Russia; 5Laboratory of Natural and Artificial Hypobiosis Mechanisms, Institute of Cell Biophysics, FRC PSCBR, Russian Academy of Sciences, Pushchino, Moscow Region, 142290, Russia


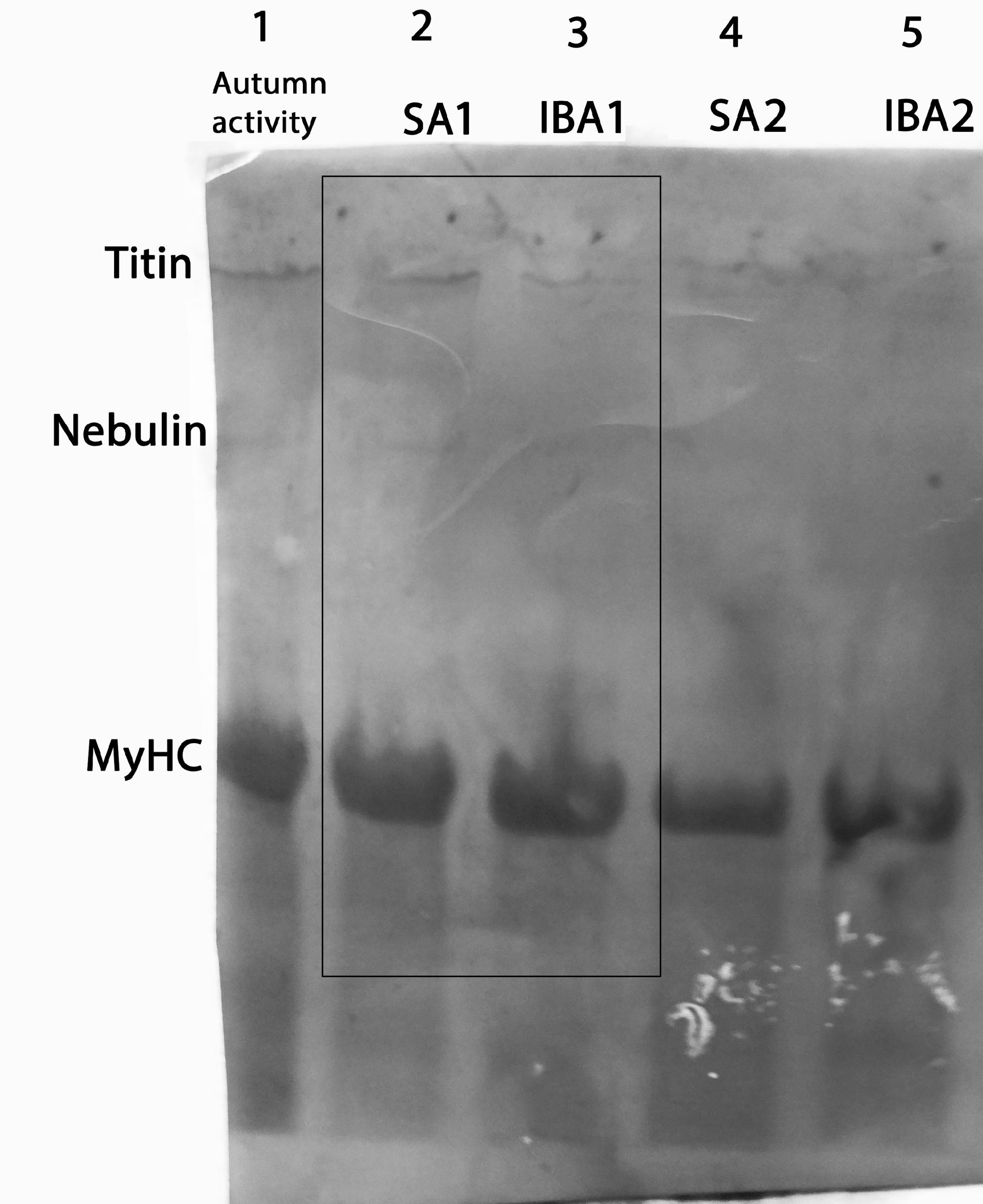


**Supplementary Fig. S20**. Part of the Ponceau S stained membrane (m. longissimus dorsi). Lanes 2 and 3 were taken for Figure 7B in the article. SA, summer activity; IBA, interbout arousal. MyHC, myosin heavy chains.


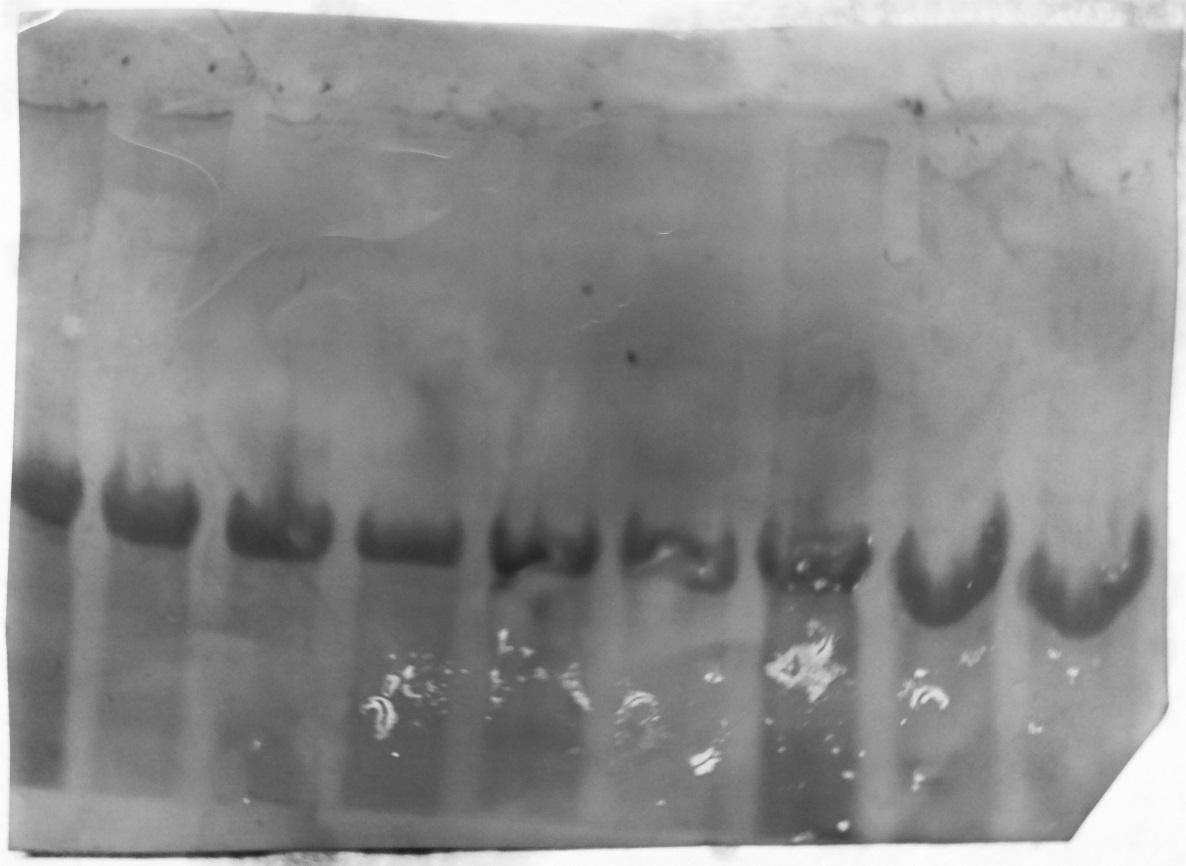


**Supplementary Fig. S21**. Full-length Ponceau S stained membrane (m. longissimus dorsi).

The original image is here:

<https://drive.google.com/open?id=1XljyCN3sfWktjuHRPACab3eQfOc6i2AC>

**Predominant synthesis of giant myofibrillar proteins in striated muscles of the long-tailed ground squirrel *Urocitellus undulatus* during interbout arousal** *Svetlana Popova1, Anna Ulanova1, Yulia Gritsyna1, Nikolay Salmov1, Vadim Rogachevsky2, Gulnara Mikhailova1, Alexander Bobylev1, Liya Bobyleva1, Yana Yutskevich3, Oleg Morenkov4, Nadezda Zakharova5 & Ivan Vikhlyantsev1,**

1Laboratory of the Structure and Functions of Muscle Proteins, Institute of Theoretical and Experimental Biophysics, Russian Academy of Sciences, Pushchino, Moscow Region, 142290, Russia; 2Laboratory of Signal Perception Mechanisms, Institute of Cell Biophysics, FRC PSCBR, Russian Academy of Sciences, Pushchino, Moscow Region, 142290, Russia; 3Kuban State University, Krasnodar, Krasnodar Krai, 350040, Russia; 4Laboratory of Cell Culture and Tissue Engineering, Institute of Cell Biophysics, FRC PSCBR, Russian Academy of Sciences, Pushchino, Moscow Region, 142290, Russia; 5Laboratory of Natural and Artificial Hypobiosis Mechanisms, Institute of Cell Biophysics, FRC PSCBR, Russian Academy of Sciences, Pushchino, Moscow Region, 142290, Russia


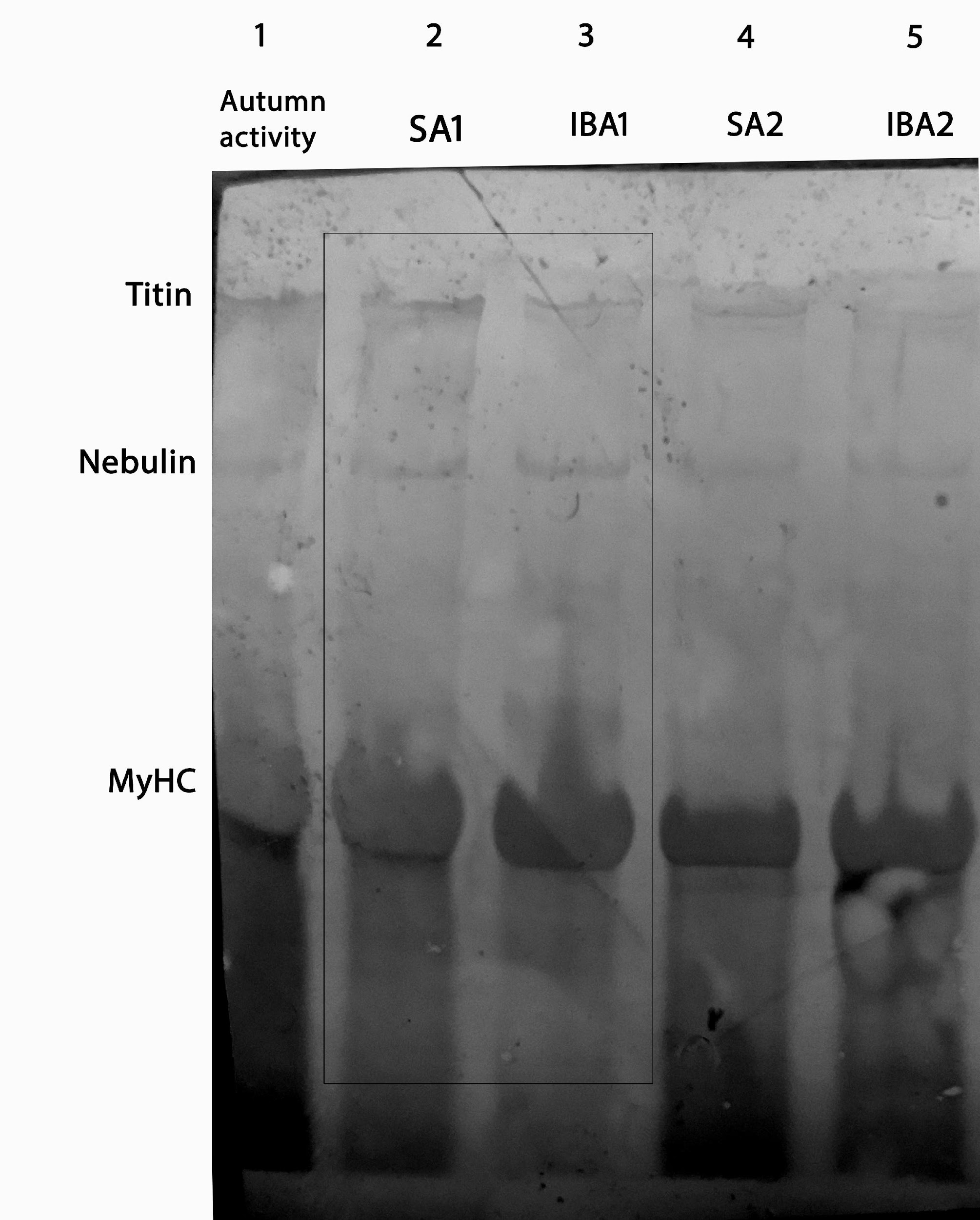


**Supplementary Fig. S22**. Part of the puromycin-stained membrane (m. longissimus dorsi). Lanes 2 and 3 were taken for Figure 7B in the article. SA, summer activity; IBA, interbout arousal. MyHC, myosin heavy chains.


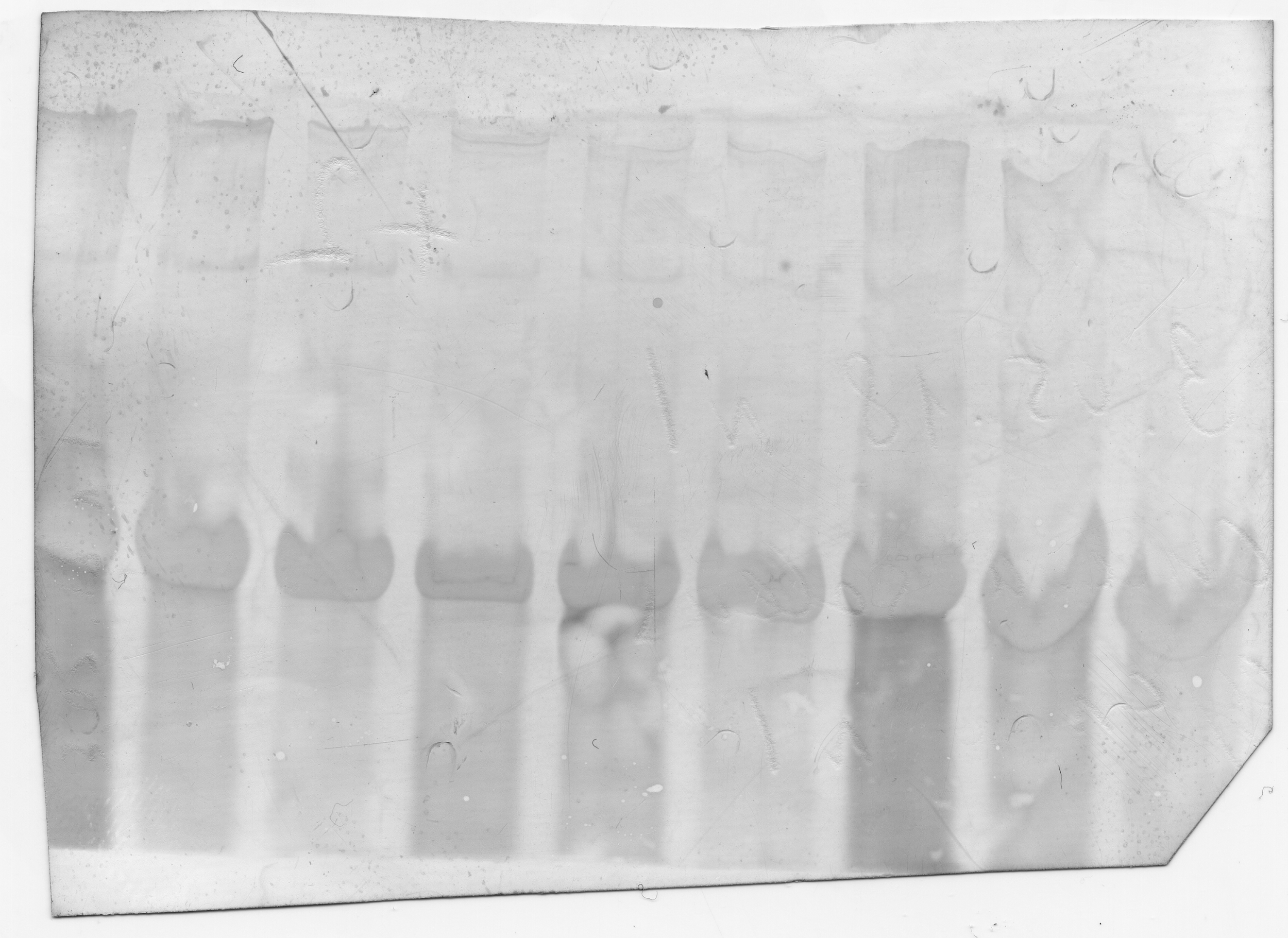


**Supplementary Fig. S23**. Full-length puromycin-stained membrane (m. longissimus dorsi).

The original image is here:

<https://drive.google.com/open?id=1XljyCN3sfWktjuHRPACab3eQfOc6i2AC>

**Predominant synthesis of giant myofibrillar proteins in striated muscles of the long-tailed ground squirrel *Urocitellus undulatus* during interbout arousal** *Svetlana Popova1, Anna Ulanova1, Yulia Gritsyna1, Nikolay Salmov1, Vadim Rogachevsky2, Gulnara Mikhailova1, Alexander Bobylev1, Liya Bobyleva1, Yana Yutskevich3, Oleg Morenkov4, Nadezda Zakharova5 & Ivan Vikhlyantsev1,**

1Laboratory of the Structure and Functions of Muscle Proteins, Institute of Theoretical and Experimental Biophysics, Russian Academy of Sciences, Pushchino, Moscow Region, 142290, Russia; 2Laboratory of Signal Perception Mechanisms, Institute of Cell Biophysics, FRC PSCBR, Russian Academy of Sciences, Pushchino, Moscow Region, 142290, Russia; 3Kuban State University, Krasnodar, Krasnodar Krai, 350040, Russia; 4Laboratory of Cell Culture and Tissue Engineering, Institute of Cell Biophysics, FRC PSCBR, Russian Academy of Sciences, Pushchino, Moscow Region, 142290, Russia; 5Laboratory of Natural and Artificial Hypobiosis Mechanisms, Institute of Cell Biophysics, FRC PSCBR, Russian Academy of Sciences, Pushchino, Moscow Region, 142290, Russia


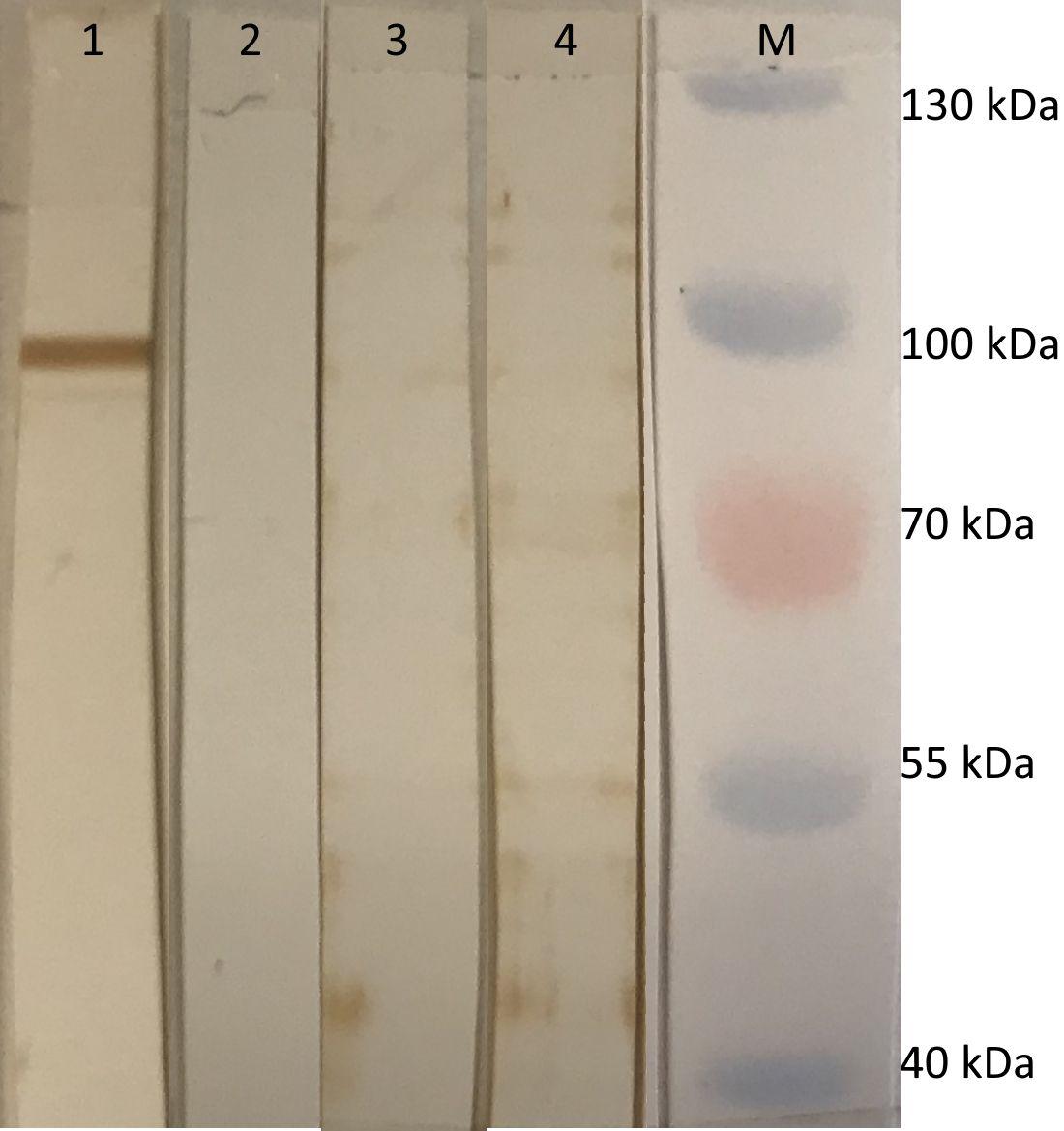


**Supplementary Fig. S24.** Lysate of human fibrosarcoma HT1080 is presented on the Figure (Western blot analysis). Membranes were incubated with various antibodies:

Positive control:

1 – Hsp90 α/β (Clone number 6H1/F8)

Negative controls:

2 – clone B32 anti BrDu

3 – 26/33 – 9B ADV

4 – 101/9 gE ADV

M – Protein Molecular Weight Marker

Description of antibodies Hsp90 α/β:

| Clone number | 6H1/F8 |
| --- | --- |
| Class/subclass | IgG1 |
| Immunogen | Human full-length recombinant Hsp90 |
| Specificity/  cross-reactivity | Antibody recognizes Hsp90 and Hsp90 of human, rat, ground squirrel, mouse, bovine, swine, hamster, and monkey origin |
| Reactivity | ELISA, WB, IF, FC |

The antibodies (Hsp90α/ß, clone number 6H1/F8) were produced at the laboratory headed by Prof. O.S. Morenkov, ICB RAS.

**Predominant synthesis of giant myofibrillar proteins in striated muscles of the long-tailed ground squirrel *Urocitellus undulatus* during interbout arousal** *Svetlana Popova1, Anna Ulanova1, Yulia Gritsyna1, Nikolay Salmov1, Vadim Rogachevsky2, Gulnara Mikhailova1, Alexander Bobylev1, Liya Bobyleva1, Yana Yutskevich3, Oleg Morenkov4, Nadezda Zakharova5 & Ivan Vikhlyantsev1,**

1Laboratory of the Structure and Functions of Muscle Proteins, Institute of Theoretical and Experimental Biophysics, Russian Academy of Sciences, Pushchino, Moscow Region, 142290, Russia; 2Laboratory of Signal Perception Mechanisms, Institute of Cell Biophysics, FRC PSCBR, Russian Academy of Sciences, Pushchino, Moscow Region, 142290, Russia; 3Kuban State University, Krasnodar, Krasnodar Krai, 350040, Russia; 4Laboratory of Cell Culture and Tissue Engineering, Institute of Cell Biophysics, FRC PSCBR, Russian Academy of Sciences, Pushchino, Moscow Region, 142290, Russia; 5Laboratory of Natural and Artificial Hypobiosis Mechanisms, Institute of Cell Biophysics, FRC PSCBR, Russian Academy of Sciences, Pushchino, Moscow Region, 142290, Russia


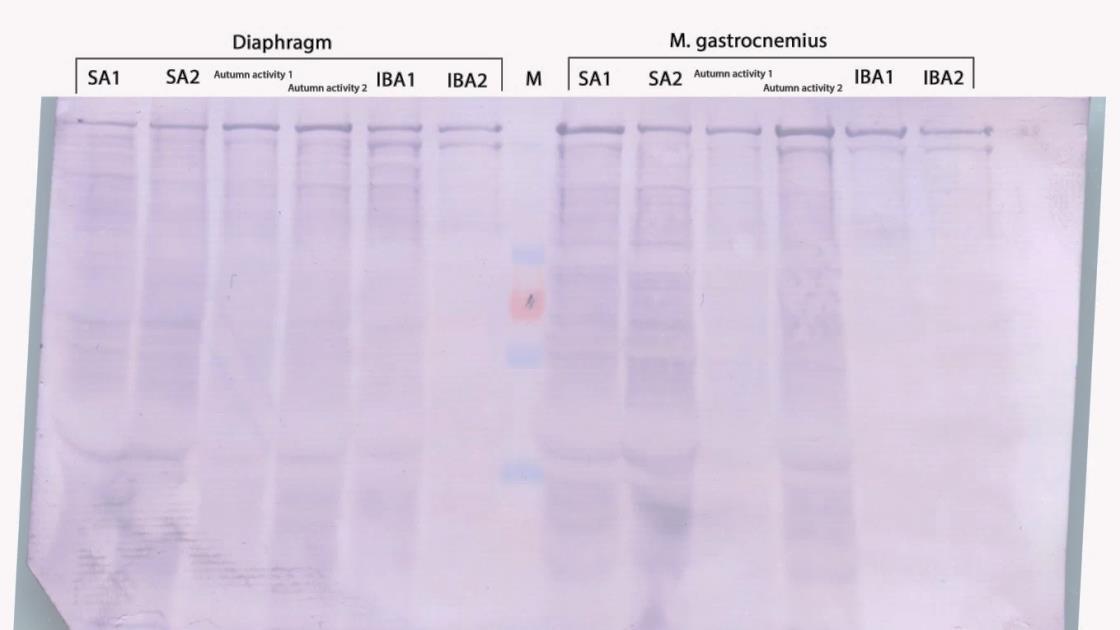


**Supplementary Fig. S25**. Rate of total protein synthesis in diaphragm and **m. gastrocnemius** muscles of ground squirrels. Full-length representative immunoblot for puromycin on PVDF membrane. The levels of proteins synthesised *in vivo* were identified relative to the total protein level. The total protein level in the samples was measured by the Bradford method. Bovine serum albumin was used as a standard. M, molecular weight marker.

SA, summer activity; IBA, interbout arousal.


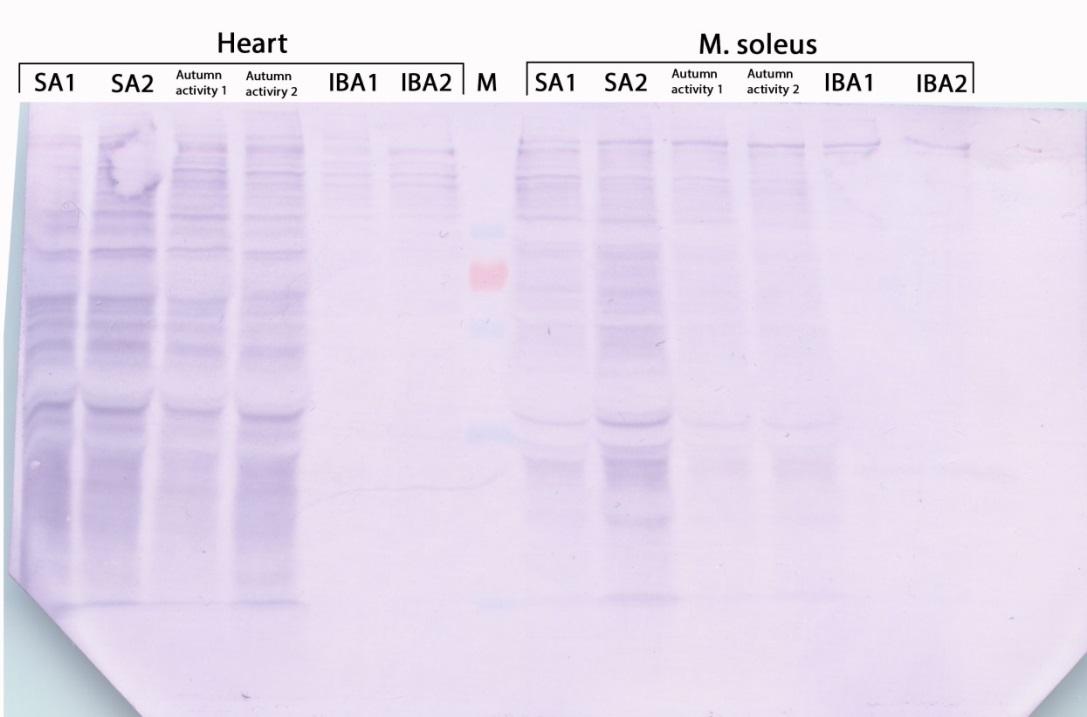


**Supplementary Fig. S26.** Rate of total protein synthesis in heart and **m. soleus** of ground squirrels. Full-length representative immunoblot for puromycin on PVDF membrane. The levels of proteins synthesised *in vivo* were identified relative to the total protein level. The total protein level in the samples was measured by the Bradford method. Bovine serum albumin was used as a standard. M, molecular weight marker.

SA, summer activity; IBA, interbout arousal.
